# Ag_2_O versus Cu_2_O in the Catalytic Isomerization of Coordinated Diaminocarbenes to Formamidines: A Theoretical Study

**DOI:** 10.3390/ma15020491

**Published:** 2022-01-10

**Authors:** Juan F. Van der Maelen, Javier Ruiz

**Affiliations:** 1 Departamento de Química Física y Analítica, Facultad de Química, Universidad de Oviedo, E-33006 Oviedo, Spain; 2 Departamento de Química Orgánica e Inorgánica, Facultad de Química, Universidad de Oviedo, E-33006 Oviedo, Spain

**Keywords:** organometallic compounds, DFT calculations, heterogeneous catalysis, computational simulations

## Abstract

DFT theoretical calculations for the Ag_2_O-induced isomerization process of diaminocarbenes to formamidines, coordinated to Mn(I), have been carried out. The reaction mechanism found involves metalation of an N-H residue of the carbene ligand by the catalyst Ag_2_O and the formation of a key transition state showing a μ-η^2^:η^2^ coordination of the formamidinyl ligand between manganese and silver, which allows a translocation process of Mn(I) and silver(I) ions between the carbene carbon atom and the nitrogen atom, before the formation of the formamidine ligand is completed. Calculations carried out using Cu_2_O as a catalyst instead of Ag_2_O show a similar reaction mechanism that is thermodynamically possible, but highly unfavorable kinetically and very unlikely to be observed, which fully agrees with experimental results.

## 1. Introduction

For many years silver(I) oxide has been widely used as a catalyst in different organic transformations, leading to the synthesis of a variety of organic molecules such as pyrroles, imidazolines, β-keto esthers, or benzothiophenes [1,2,3,4,5], as well as in many stoichiometric reactions, such as the synthesis of N-heterocyclic carbenes (NHC) based on Ag_2_O [6,7,8]. In spite of that, mechanistic studies of reactions involving Ag_2_O are really scarce, with some notable exceptions being those related to the ethylene epoxidation reaction [9,10,11] and the generation of NHC-Ag(I) complexes used in further transmetalation processes [12]. We have recently described an efficient experimental procedure to transform isocyanides into formamidines by reaction with primary amines [13,14], involving a key step tautomerization of diaminocarbenes coordinated to Mn(I), mediated by Ag_2_O, into formamidines. This behavior is apparently exclusive to Ag_2_O, as isostructural Cu_2_O does not show any activity in the same reaction, nor do other metallic oxides of variable nature.

**Scheme 1:**
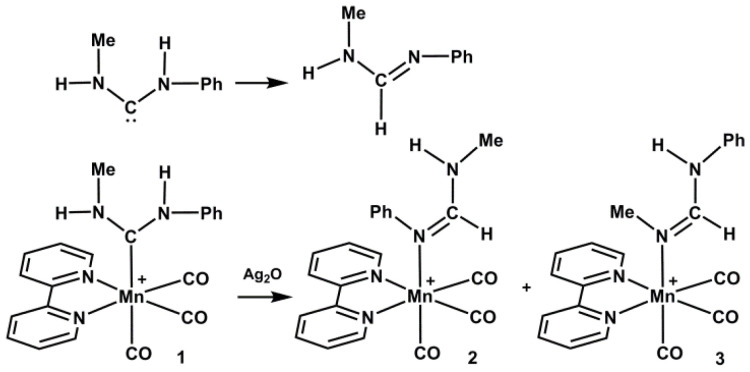
Transformation of free and coordinated diaminocarbenes into formamidines.

Free protic diaminocarbenes are unknown, as their formamidine tautomers are much more stable [15]. On the contrary, protic diaminocarbene complexes are very stable [16,17] and, as shown in the present case, can be transformed into the corresponding formamidine complexes by addition of a catalyst (Scheme 1). Interestingly, we have similarly demonstrated that coordinated NHCs isomerize to imidazoles under the same conditions [13,14]. In order to shed light on the mechanism of this intriguing reaction and to make a contribution to the rather scarcely developed field of mechanistic studies of organic and organometallic transformations assisted by Ag_2_O, we have now carried out DFT theoretical calculations supporting the finding that the exclusive behavior of Ag_2_O in the catalytic isomerization of diaminocarbenes to formamidines is essentially based on an ideal combination of the basic character of the oxide ion and the Lewis acid character of the Ag^+^ ion. Both features promote, respectively, proton transfer from nitrogen to carbon and carbene carbon atom transfer from Mn(I) to Ag(I) preceding the ultimate formation of formamidines.

## 2. Experimental Section

**Figure 1:**
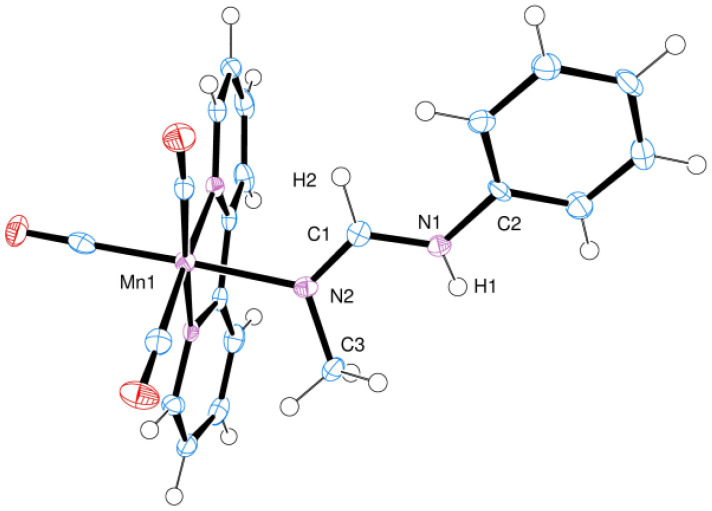
ORTEP plot of the X-ray molecular structure of cation **3** (CCDC deposition number 2044950) showing the atomic numbering scheme used for all compounds (ellipsoids at 50% probability). Selected bond distances (Å): Mn1–N2, 2.084(3); N2–C1, 1.288(4); N2–C3, 1.478(5); C1–N1, 1.356(4); C1–H2, 1.03(3); N1–H1, 0.87(4); N1–C2, 1.420(4). Selected bond angles (°): Mn1–N2–C1: 123.0(2); Mn1–N2–C3: 119.6(2); C3–N2–C1: 117.4(3); N2–C1–N1: 125.6(3); C1–N1–C2: 125.5(3); N2–C1–H2: 119(2).

The detailed experimental procedure for the synthesis and spectroscopic structural characterization (FTIR and NMR) of **1**, **2**, and **3** complexes has been described previously [13,14]. Selected crystallographic data and structure refinement parameters for **3**·${\mathrm{ClO}}_{4}^{-}$ are given here for the first time in Table S1 of the Supplementary Material, while Figure 1 shows the experimental molecular structure together with the atomic numbering scheme and selected bond lengths and angles. Additional details of the X-ray crystal structure determination of **3**·${\mathrm{ClO}}_{4}^{-}$ are given in the CIF file (CCDC deposition number 2044950). The rather disordered floppy ligands could be finally modeled by means of convenient constrained/restrained algorithms. Further details about the refinement protocols used may be found elsewhere [18,19].

## 3. Computational Details

Density functional theory (DFT) computations have been performed with the GAUSSIAN09 and ADF2012 program packages [20,21], starting from our experimental geometry for cation **3**, here included, and from the one of a derivative of cation **1**, obtained from X-ray single-crystal diffraction data and previously published [22], using the B3P86-D3(BJ) Becke´s three-parameter exchange functional with the non-local Perdew correlation functional, as well as the M06-D3 hybrid functional of Truhlar and Zhao [23,24,25,26], which are both well known for obtaining reliable results with transition-metal organometallic compounds [27,28,29,30]. The standard all-electron 6-31+G(d) and 6-311++G(2df,2pd) basis sets were employed for C, H, N, O, Cu, and Mn atoms at different steps of the procedure. As it is usual practice when large compounds involving transition-metal atoms is concerned, for geometry optimizations, the smaller basis set was used, while, for the final single-point electronic structure calculations, the larger basis set was utilized, since it would be impossible for the optimization process to either converge or finish at a reasonable time if the larger basis set were used for the geometry optimization processes [31,32,33,34]. The LanL2DZ effective core potential and the large all-electron AQZP basis set were used for the Ag atom [35,36] (again, the former in the geometry optimization and the latter in the single-point electronic structure calculations).

The usual Berny algorithm with the quasi-Newton rational function optimization (RFO) method and the Pulay´s Direct Inversion procedure for SCF cycles were used for most of the optimizations made, although in some cases (the saddle points of reaction paths), the slower but more reliable quadratically convergent SCF procedure and the Newton–Raphson optimization algorithm were utilized instead [37,38]. Vibrational analysis was performed at every stationary point found, within the B3P86-D3(BJ)/6-31+G(d)/LanL2DZ(Ag) level of theory, characterizing intermediate compounds **I1**, **I2**, **I3**, **I4**, and **I5** as minima (no imaginary frequencies) in mechanism *M1*, intermediate compounds **I6**, **I7**, **I8**, **I9**, and **I10** as minima in mechanism *M2*, and intermediate compounds **I11** and **I12** as minima in (partial) mechanism *M3*. The same level of theory was used to simulate modeling reactants **1** + Ag_2_O in mechanisms *M1* and *M3*, reactants **1** + Cu_2_O in mechanism *M2*, products **2** + Ag_2_O, **2′** + Ag_2_O and **2″** + Ag_2_O in mechanism *M1*, products **2** + Cu_2_O, **2′** + Cu_2_O and **2″** + Cu_2_O in mechanism *M2*, and products **3** + Ag_2_O in mechanism *M3*. Subsequently, using these minima as starting points, transition states (each one of them having one imaginary frequency) were searched between minima in the three mechanisms within the B3P86/LanL2DZ level of theory. Additionally, application of the intrinsic reaction coordinate (IRC) method showed that each transition state found in reaction paths effectively connects with their corresponding minima [39,40]. All reactants, products, intermediates, and transition states have an electric charge of +1 *e*, except for Ag_2_O and Cu_2_O, and also have a singlet state as the ground state.

**Figure 2:**
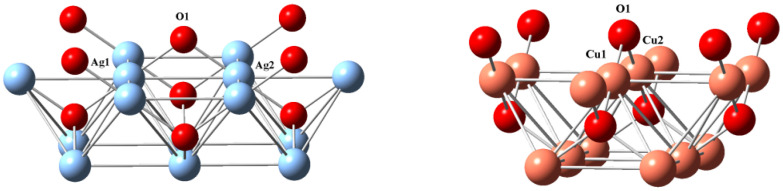
Cluster surface (001) of catalysts, using experimental lattice parameters *a* = 4.7230 Å (Ag_14_O_9_) and 4.2696 Å (Cu_14_O_9_). Color codes: O (red), Ag (light blue), and Cu (coppery). Two oxygen atoms symmetrically located at the upper corners were deleted in the simulation.

Every ground state wavefunction obtained for each compound was analyzed and found to be stable. Solvent effects were taken into account using the polarizable continuum model (PCM) with ε = 8.93 (dichloromethane) [41]. The anion (${\mathrm{ClO}}_{4}^{-}$) contribution to the energy has not been taken into account, since the classical electrostatic interaction between anion and cations is assumed to be approximately constant along the three reactions in solution (this contribution may be estimated to be, using pure Coulomb classical approximation, −12.5 kcal mol^−1^ from the Cl^−^⋯⋯H^+^ experimental distance in compound **3**·${\mathrm{ClO}}_{4}^{-}$: 2.973 Å). The simulation of catalysts Ag_2_O and Cu_2_O started by theoretically optimizing the experimental geometry from their crystalline structures, following a procedure similar to the one made on previous theoretical studies [9,10,11,12]. The additional stabilizing effect of the lattice for the catalytic action has been estimated by the simulation of Ag_14_O_7_ and Cu_14_O_7_ (001) solid surfaces (Figure 2), using the model M06-D3/6-311++G(3df,3pd)/QZPDKH(Ag) [42,43], to be −124.5 kcal mol^−1^ and –156.8 kcal mol^−1^, respectively.

Scaled zero-point energies (ZPE) and thermal components (translational, rotational, and vibrational) were added to the final electronic energies in order to obtain reliable enthalpies and Gibbs free energies at room temperature and normal pressure (scale factor used for ZPE: 0.9804) [44]. Gibbs energies were determined, as it is usual practice, by adding the translational, rotational, and vibrational components of the total energy (plus ZPE and the entropy term), calculated at every stationary point found using the smaller basis set, to the electronic part of the total energy, which has been calculated using the larger basis set, as explained above. In order to check the reliability of the results, two different DFT functionals have been used, the B3P86-D3(BJ) functional and the M06-D3 functional, giving results really close to each other, showing differences not higher than 0.1 kcal mol^−1^. The B3P86-D3(BJ)/6-311++G(2df,2pd)/AQZP(Ag) is the final model adopted for further discussion in the main text of this paper, while in the Supplementary Material, results using the M06-D3 functional are included (see Tables S9–S11).

## 4. Results and Discussion

### 4.1. Ag_2_O Mechanism M1

**Scheme 2:**
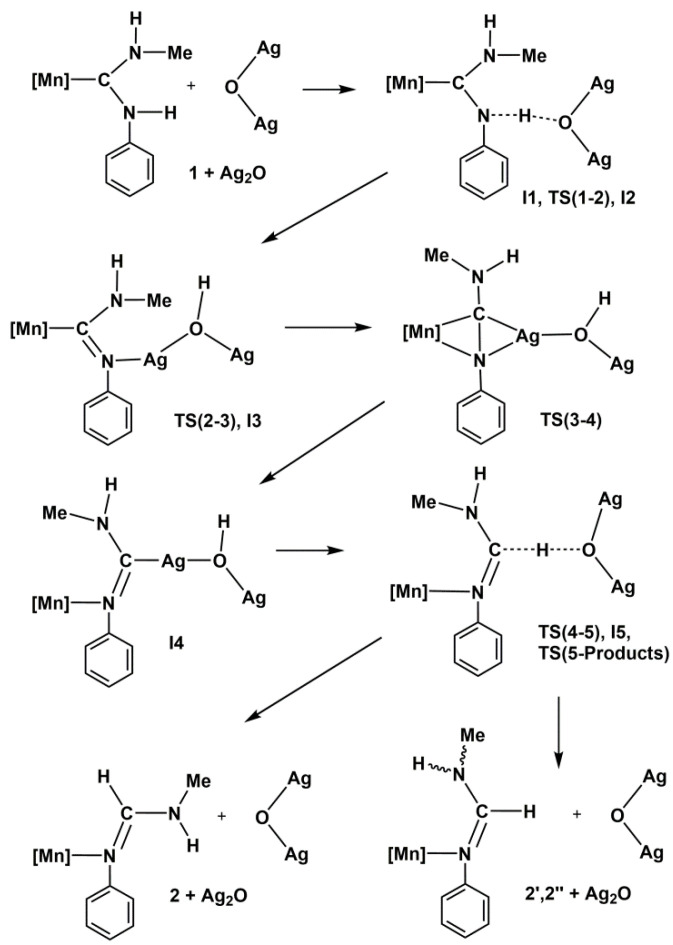
Proposed mechanism for the isomerization of the diaminocarbene ligand in complex **1** to formamidine, catalyzed by Ag_2_O. [Mn] = [Mn(CO)_3_(bipy)]^+^.

**Figure 3:**
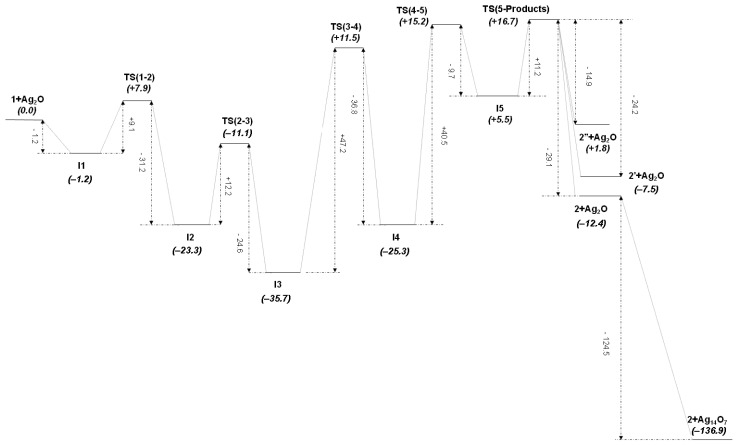
Graphic representation of the *M1* mechanism at the B3P86-D3(BJ)/6-311++G(2df,2pd), AQZP(Ag) level of theory ($\Delta G_{298}^{0}$ values in kcal mol^−1^).

**Figure 4:**
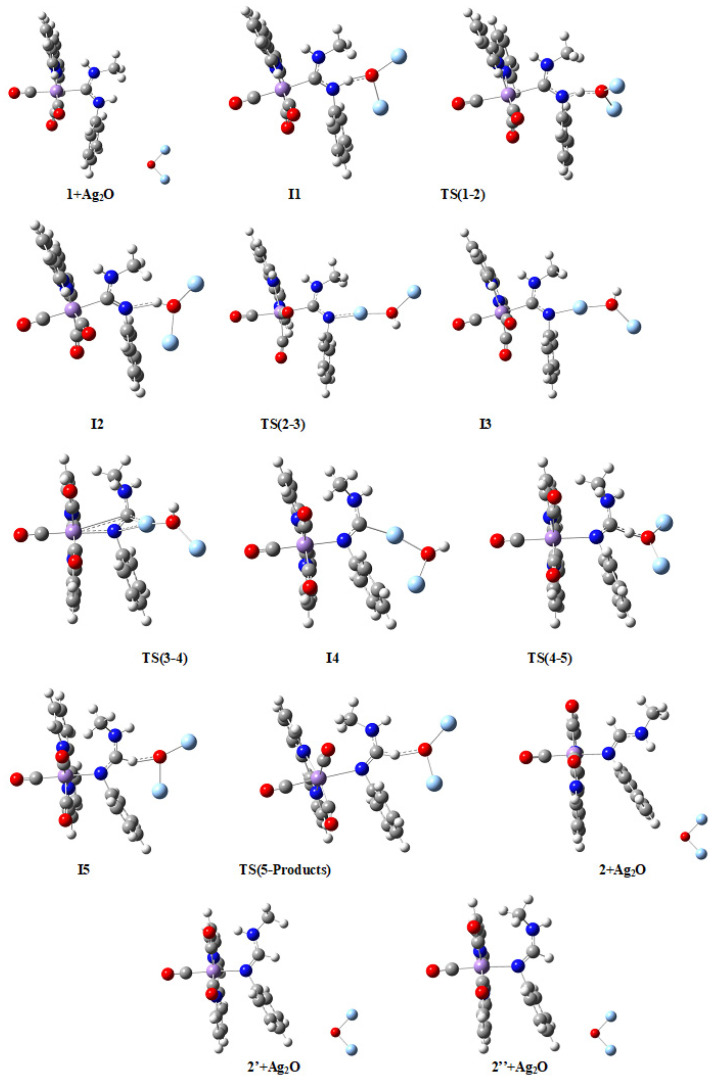
Molecular structures of compounds in the *M1* mechanism. Larger figures are given in the Supplementary Material (Figure S1). Color codes: Mn (magenta), C (grey), O (red), N (dark blue), H (white), and Ag (light blue).

The overall transformation of diaminocarbene complex **1** into formamidine derivative **2** mediated by Ag_2_O is summarized in Scheme 2. The graphic representation of this *M1* mechanism may be seen in Figure 3, while theoretically optimized geometries for all molecules involved are depicted in Figure 4, and selected bond distances and angles are collected in Table 1 and Table 2, respectively (See Figure 1 for the atomic numbering scheme used in all tables and the forthcoming discussion). Bearing in mind that the reaction path follows the sequence (**1 + Ag_2_O)**—**I1**—**TS(1-2)**—**I2**—**TS(2-3)**—**I3**—**TS(3-4)**—**I4**—**TS(4-5)**—**I5**—**TS(5-Products)**–{**2 + Ag_2_O, 2′ + Ag_2_O, 2″ + Ag_2_O}**—{**2 + Ag_14_O_7_, 2′+Ag_14_O_7,_ 2″ + Ag_14_O_7_}**, the most relevant features from Figure 4 and Table 1 are the following. The tautomerization process from cation **1** to cation **2** may be divided into two main sequences. The first sequence goes from cation complex **1**, where the Mn atom of the [Mn]^⊕^ group ([Mn]^⊕^ = [Mn(CO)_3_(bipy)]^⊕^) is bonded to the C1 atom of the acyclic diaminocarbene (ADC) ligand, onto intermediate **I4**, where the Mn atom is bonded to the N1 atom of the same ligand in its deprotonated form and the Ag atom is bonded to the carbene carbon atom C1, with the reaction path passing through another three intermediates and three transition states (see Table 1). In this sequence there is first a relatively easy and fast proton transfer from the reactant (**1**) to the catalyst (**Ag_2_O**) (see Figure 3, where low energetic barriers are shown to reach intermediate **I3**) through a significant increase of the N1-H1 bond distance, from the typical covalent bond value of 1.015 Å in **1** to the hydrogen-bond value of 2.192 Å in **I2**. At the same time, H1-O1 distance decreases from 1.582 Å in **I1** to 0.983 Å in **I2**. The proton transfer is totally completed in intermediate **I3** (H1-O1 0.967 Å) with parallel coordination of N1 to the silver atom (N1-Ag1 2.134 Å). However, the critical step in the first sequence of the mechanism is given in its second part by the formation of the transition state **TS(3-4)**, for which both Mn1 and Ag1 atoms are attached to both N1 and C1 atoms, a structure where Mn1-C1 and Ag1-N1 bonds are simultaneously breaking, at the same time as Mn1-N1 and Ag1-C1 bonds are forming. On the other hand, it is noteworthy that both C1-N1 and C1-N2 bond distances, as well as N1-C1-N2 bond angle, do not change significantly along this whole first sequence of the mechanism, not even in the transition states (see Tables S3 and S4 of the Supplementary Material).

The second sequence of the *M1* mechanism starts from the intermediate **I4** with a rotation of the protonated catalyst in its own plane until it reaches an orientation useful to create the hydrogen bond O1-H1…C1 in the transition state **TS(4-5)**, followed by the gradual transformation of this interaction into a C1-H1 pure covalent bond, and the forthcoming separation of product and catalyst. Along this process, the C1-H1 bond distance decreases from 1.856 Å in **TS(4-5)** to 1.087 Å in product **2** (see Table 1), while H1-O1 bond distance consequently increases from 0.968 Å in **I4** to 1.807 Å in **TS(5-Products)**.

Rather interestingly, two alternative products (**2′** and **2″**) are in principle possible (see Figure 4), although they are less stable than product **2** (Figure 3). As may be seen in Table 1, bond distances and angles are quite similar for the three possible products. Nevertheless, the main structural difference between these products is the relative orientation of the NHMe group with respect to the metallic fragment, which is located *anti* in **2** and *syn* in **2′** and **2″**, thus minimizing the steric hindrance, and so the repulsion energy, in the more stable product **2**. For their part, compounds **2′** and **2″** differ in the relative orientation of C1-H1 and N2-H2 groups in the formamidine ligand, which are *trans* in **2′** and *cis* in **2″** (H1-C1-N2-H2 torsion angle is −171.1° in **2′** and 2.6° in **2″**; see Figure 4), which in turn leads to methyl and phenyl groups in the same ligand to also have different relative orientations in both products (*syn* in **2′** and *anti* in **2″**). Therefore, the methyl group is farther from the bipy ligand of the [Mn]^⊕^ group in **2′** than in **2″**, thus minimizing the repulsion energy between methyl and bipy ligands in the former.

From the foregoing discussion, it is not surprising that the thermodynamically preferred path in mechanism *M1* leads to product **2**, with the most negative value of $\Delta G_{298}^{0}\left(1\to 2\right)=-12.4{\;\mathrm{kcal}\;\mathrm{mol}}^{-1}$ found for this exergonic process, instead to product **2′**, with a value of $\Delta G_{298}^{0}\left(1\to 2^{\prime }\right)=-7.5{\;\mathrm{kcal}\;\mathrm{mol}}^{-1}$, or to product **2″** with a value of $\Delta G_{298}^{0}\left(1\to 2^{\prime \prime }\right)=+1.8{\;\mathrm{kcal}\;\mathrm{mol}}^{-1}$ found for this unfavorable path (see Figure 3), a result in complete agreement with experimental data, where only a product is observed compatible with the current mechanism; i.e., product **2** (but see below, mechanism *M3*, for completely different products, starting from the same reactants) [13,14]. Calculated standard Gibbs free energies at room temperature for all compounds along the favorable path of the *M1* mechanism are collected in Figure 3 and in Table S9, showing $\Delta G_{298}^{0}(Intermediate\to TS)$ values of the same order of magnitude than those found in similar reactions involving the bulky [Mn(CO)_3_(bipy)]^⊕^ group, although slightly higher [45,46]. Since the estimated lattice stabilization energy for the catalyst is of the order of one hundred kcal mol^−1^, as already mentioned in the Computational Details section, it is hardly surprising that transition barriers are of the order of several tenths of kcal mol^−1^. As already mentioned in the Introduction and Computational Details sections, the experimentally observed process studied in previous papers is in fact heterogeneously catalyzed by a solid Ag_2_O surface [13,14], while the simulation performed currently is made in a simplified homogeneous media instead; therefore, the energy barriers are consequently much higher here than in the real physical process, which is computationally unaffordable using ab initio methods. Very likely, dispersion and other non-covalent long-range interactions between the solid surfaces and the reactants (and possibly with the solvent and the perchlorate anion as well), which could not be properly modeled by the state-of-the-art ab initio methods, reduce the high barriers in the experimentally observed process, but they scarcely affect the proposed mechanism and, more importantly, the energy differences calculated between reactants and products. Therefore, this is clearly a kinetically-controlled process that could not be experimentally observed in a reasonable time at room temperature in the absence of the surface provided by the solid catalyst. Two rate-determining steps with similar energy barriers are found in this mechanism: the fourth step (**I3** → **I4**), which proceeds through transition state **TS(3-4)** with the simultaneous breaking of Mn1-C1 and Ag1-N1 bonds, and the fifth step (**I4** → **I5**), which proceeds through transition state **TS(4-5)** with the additional breaking of the Ag1-C1 bond. On the other hand, the product-determining step is the sixth one (**I5** → **2+Ag_2_O**) since all previous steps are common to the three possible outcomes **2**, **2′**, and **2″**. In addition, due to the steric restrictions involving the rotation of Ag_2_O between intermediates I2 and I3, the reaction is very likely to proceed at the edges and defects of the catalyst surface.

### 4.2. Cu_2_O Mechanism M2

**Scheme 3:**
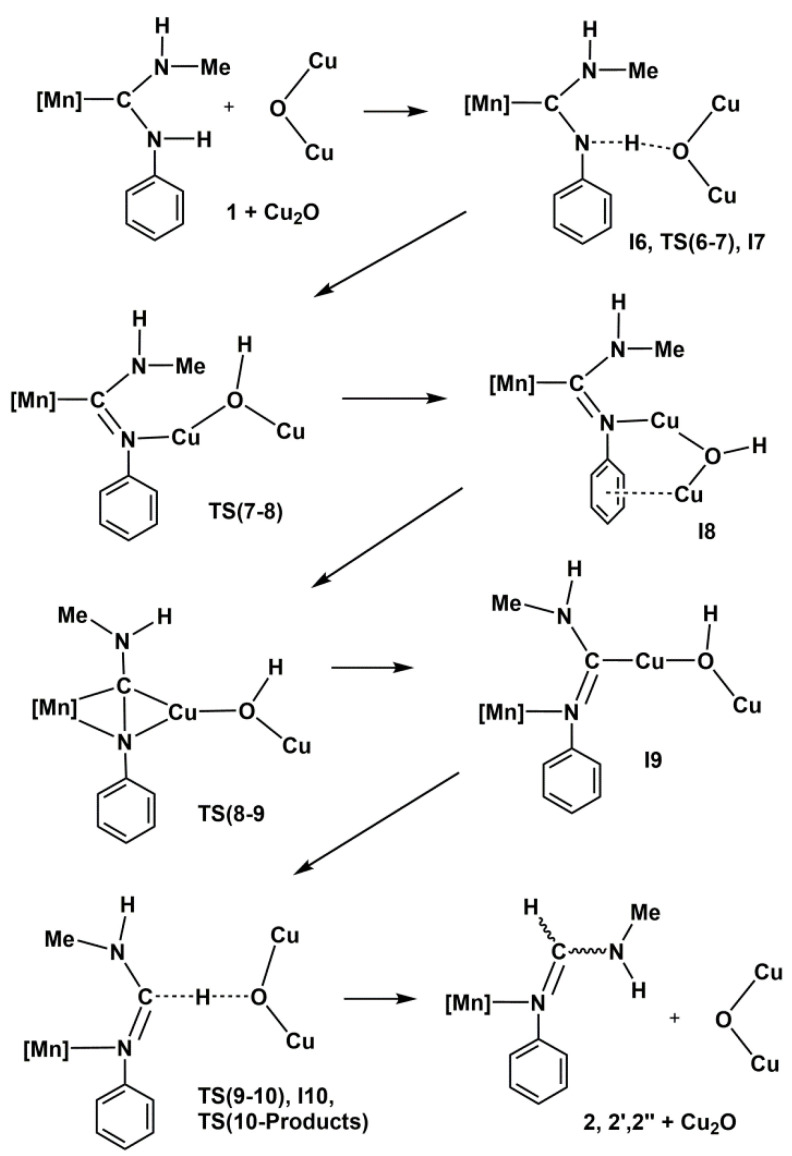
Proposed mechanism for the isomerization of the diaminocarbene ligand in complex **1** to formamidine catalyzed by Cu_2_O. [Mn] = [Mn(CO)_3_(bipy)]^+^.

**Figure 5:**
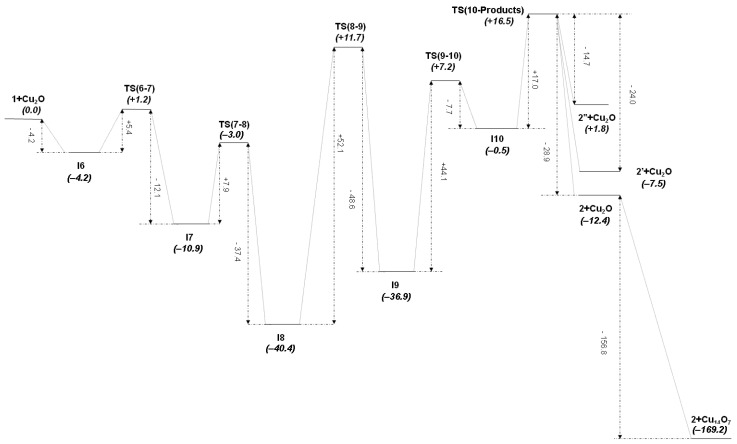
Graphic representation of the *M2* mechanism at the B3P86-D3(BJ)/6-311++G(2df,2pd) level of theory ($\Delta G_{298}^{0}$ values in kcal mol^−1^).

**Figure 6:**
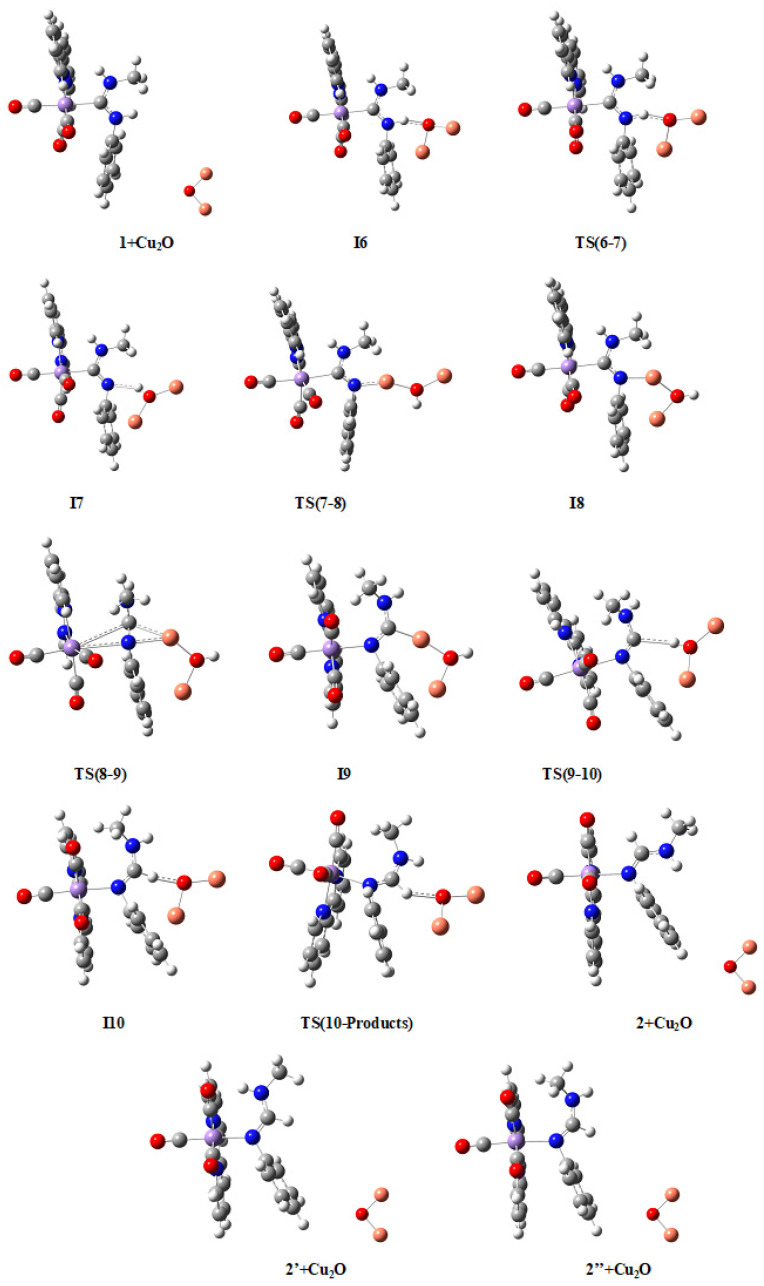
Molecular structures of compounds in the *M2* mechanism. Larger figures are given in the Supplementary Material (Figure S2). Color codes: Mn (magenta), C (grey), O (red), N (dark blue), H (white), and Cu (coppery).

The overall transformation of diaminocarbene complex **1** into formamidine derivative **2** mediated by Cu_2_O is summarized in Scheme 3. The graphic representation of the *M2* mechanism may be seen in Figure 5, while theoretically optimized geometries for all molecules involved are depicted in Figure 6, and selected bond distances and angles are collected in Table 3 and Table 4, respectively.

Bearing in mind that the reaction path follows the sequence (**1** + Cu_2_O)—**I6**—**TS(6-7)**—**I7**—**TS(7-8)**—**I8**—**TS(8-9)**—**I9**—**TS(9-10)**—**I10**—**TS(10**-Products)-{**2** + Cu_2_O, **2**’ + Cu_2_O, **2**’’ + Cu_2_O}-{**2** + Cu_14_O_7_, **2**’ + Cu_14_O_7,_ **2**’’ + Cu_14_O_7_}, the most relevant features from Figure 6 and Table 3 are the following. The tautomerization process from cation **1** to cation **2** using Cu_2_O as catalyst instead of Ag_2_O follows qualitatively, at least from a geometrical point of view, a mechanism closely related to the previously detailed *M1* mechanism, taking place also here through five intermediate complexes, **I6** to **I10**, which may be divided again into two main sequences. The first sequence goes from cation complex **1**, where the Mn atom of the [Mn]^⊕^ group is bonded to the C1 atom of the ADC ligand, onto intermediate **I9**, where the Mn atom is bonded to the N1 atom of the same ligand, with the reaction path passing through another three intermediates and three transition states (see Table 3). Within this sequence there is first a proton transfer from the reactant (**1**) to the catalyst (Cu_2_O) through a significant increase of the N1-H1 bond distance, from the typical covalent bond value of 1.015 Å in **1** to the hydrogen-bond value of 2.067 Å in **I7**. At the same time, H1-O1 distance decreases from 1.678 Å in **I6** to 0.966 Å in **I9**. In the first part of this sequence, ending in intermediate **I7**, H1 atom is completely transferred from the N1 atom of the ADC ligand to the O1 atom of the Cu_2_O catalyst (see Figure 6). However, here again the critical step in the first sequence of the mechanism is given in its second part by the formation of the transition state **TS(8-9)**, for which both Mn1 and Cu1 atoms are attached to both N1 and C1 atoms, a structure where Mn1-C1 and Cu1-N1 bonds are simultaneously breaking, at the same time as Mn1-N1 and Cu1-C1 bonds are forming (see Figure 5 for a view of the energy barrier involved in this step). On the other hand, it is noteworthy that both C1-N1 and C1-N2 bond distances, as well as N1-C1-N2 bond angle, not only do not change significantly along this first sequence of mechanism *M2*, but also are nearly equal to the ones of mechanism *M1* (compare their values in Tables S5 and S6 with those in Tables S3 and S4 of the Supplementary Material). For instance, the N1-C1-N2 angle is 123.3° in **I9** (mechanism *M2*) and 121.9° in **I4** (mechanism *M1*).

The second sequence of the *M2* mechanism starts, similarly to mechanism *M1*, from the intermediate **I9** with a rotation of the protonated catalyst in its own plane until it reaches an orientation useful to create the hydrogen bond O1-H1⋯C1 in the transition state **TS(9-10)**, followed by the gradual transformation of this interaction into a C1-H1 pure covalent bond, and the forthcoming separation of product and catalyst. Along this process, the C1-H1 distance decreases from 2.297 Å in **TS(9-10)** to 1.092 Å in product **2** (see Table 3), while H1-O1 distance consequently increases from 0.966 Å in **I9** to 2.096 Å in **TS(10-Products)**. Note that exactly the same main products (**2**, **2′** and **2″**) are obtained through both mechanisms *M2* and *M1*. Similarly to mechanism *M1*, due to the steric restrictions, involving in the current case the rotation of Cu_2_O between intermediates I7 and I8, the reaction is very likely to proceed at the edges and defects of the catalyst surface.

Calculated standard Gibbs free energies at room temperature for all compounds along the thermodynamically favorable path of the *M2* mechanism are collected in Figure 5 and in Table S10, showing $\Delta G_{298}^{0}(Intermediate\to TS)$ values clearly higher than the ones obtained in mechanism *M1* (compare Figure 3 and Figure 5). Considering the two rate-determining steps (fourth and fifth steps), the quotient between rate constants in both mechanisms is [47]:

$$\begin{array}{cc} \frac{k_{M2}\left(I8\to TS(10-Products)\right)}{k_{M1}\left(I3\to TS(5-Products)\right)} & =\exp[-(\Delta G_{298}^{0}(I8\to TS(10-Products))-\Delta G_{298}^{0}(I3\to TS(5-Products)))/RT]\\ & \approx 5\times 10^{-4}. \end{array}$$

**Figure 7:**
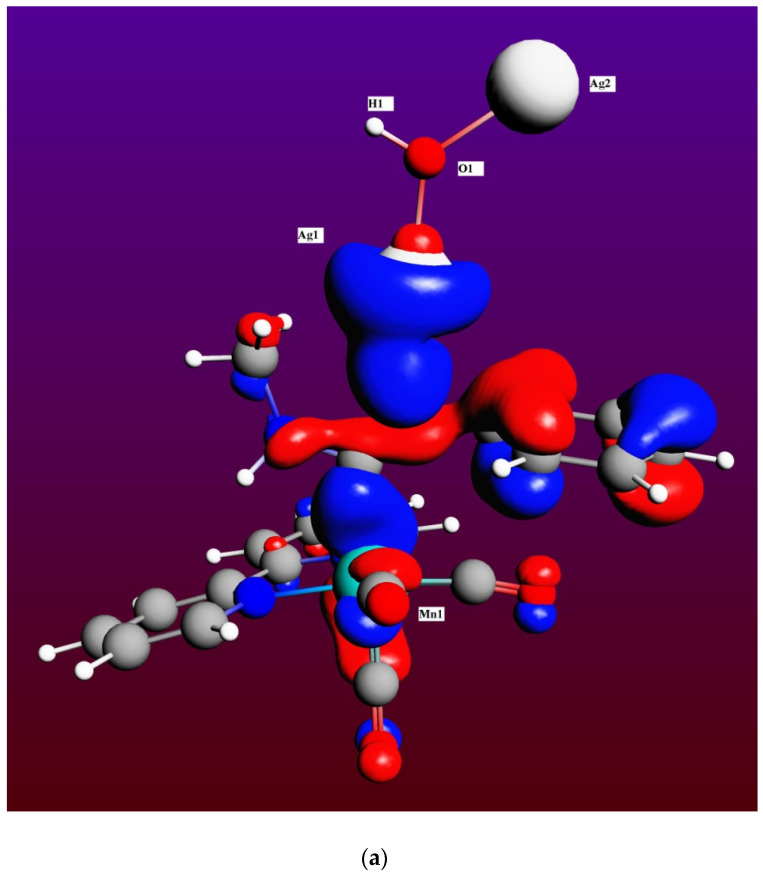

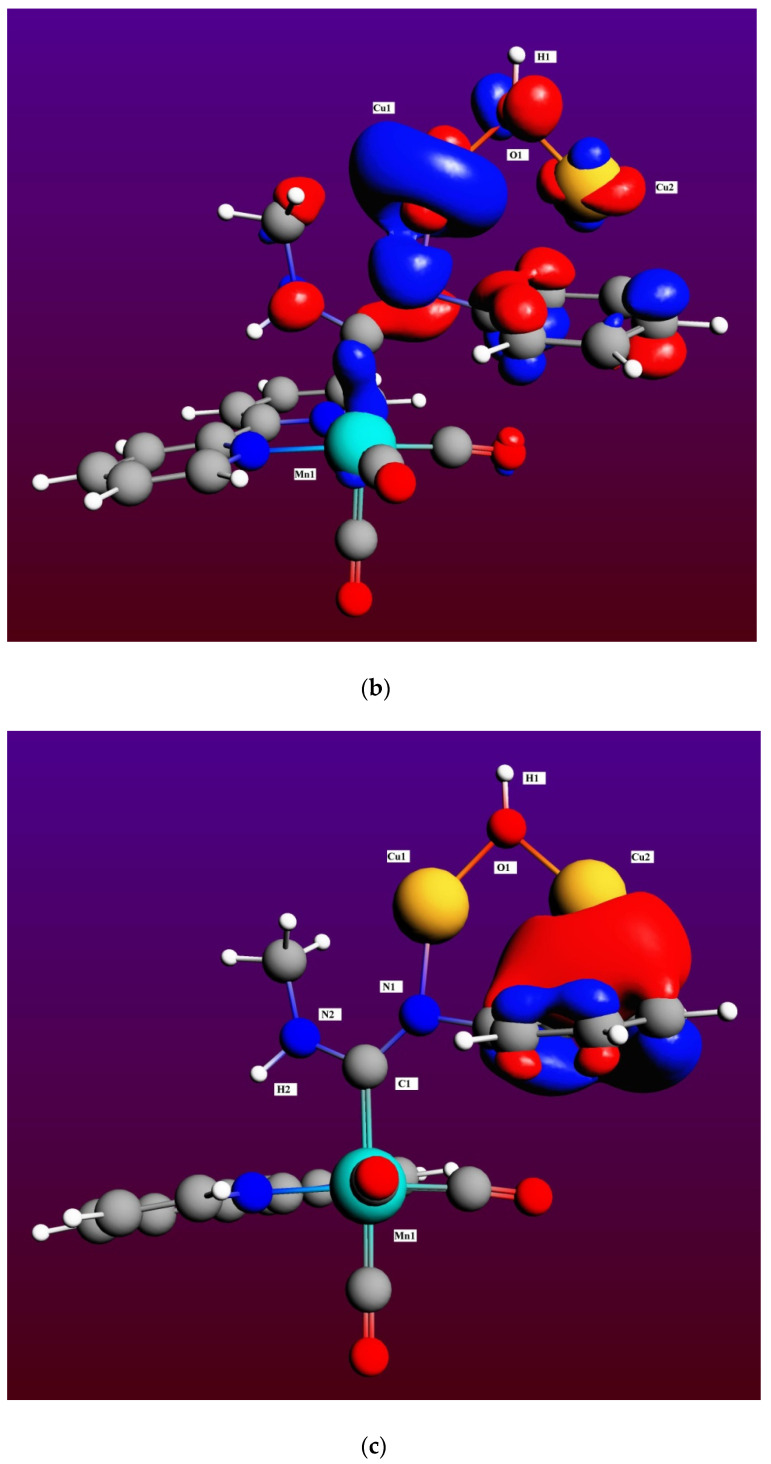
Representation of the canonical HOMO molecular orbital of intermediates **I3** (**a**) and **I8** (**b**), showing the contributions from different atoms, and the Boys-Foster localized molecular orbital accounting for the Cu2-phenyl group interaction in **I8** (**c**). Color codes: Mn (cyan), C (dark grey), O (red), N (dark blue), H (white), Ag (light grey), and Cu (orange).

Therefore, the *M2* mechanism, although thermodynamically possible, is highly unfavorable kinetically and very unlikely to be observed experimentally at room temperature, even in the presence of a catalyst’s solid surface [13,14]. By comparing equivalent intermediates in both mechanisms for the fourth step, **I3** and **I8** (see Figure 4 and Figure 6, respectively), it is clearly seen an attractive interaction that may be present in **I8** between the Cu2 atom and the phenyl group of the ADC ligand (Cu2-C* distance is 2.503 Å, with C* the centroid of the phenyl group), which will reasonably be absent in **I3** between the Ag2 atom and the phenyl group, much stabilizing then the Cu intermediate with respect to its transition state **TS(8-9)**, and thus explaining the larger difference in rate constants for this step between both mechanisms. In fact, as may be clearly seen in Figure 7a,b, Cu2′s atomic orbitals (AOs) contribute appreciably to the HOMO molecular orbital in **I8**, while Ag2′s AOs do not contribute at all in **I3**. Moreover, the Boys–Foster localized MO depicted in Figure 7c shows rather neatly this interaction, which is present in the Cu intermediate but absent in the Ag one. An additional contribution for the stabilization of intermediate **I8** respect to **I3** may be the dissociation energy of the Cu(I)-N bond, which is higher than that of the Ag(I)-N bond for a variety of nitrogen ligands [48,49,50].

### 4.3. Ag_2_O Mechanism M3

**Figure 8:**
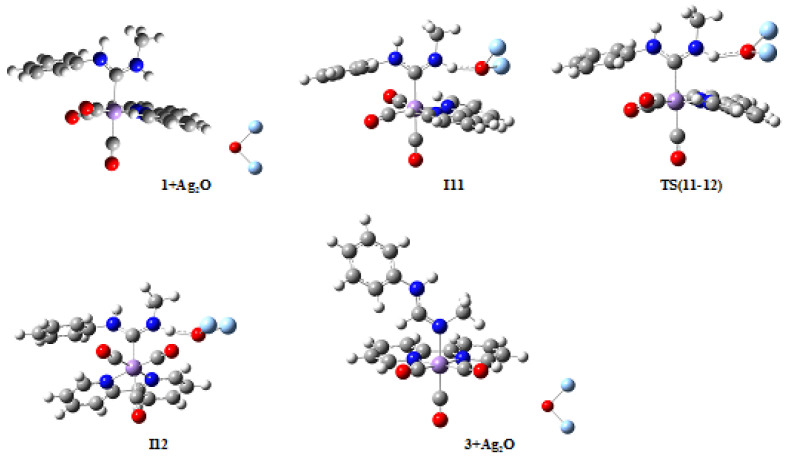
Molecular structures of compounds in the *M3* mechanism. Larger figures are given in the Supplementary Material (Figure S3). Color codes: Mn (magenta), C (grey), O (red), N (dark blue), H (white), and Ag (light blue).

As mentioned above, another product is observed experimentally for the tautomerization of cation **1** catalyzed by Ag_2_O; namely, compound **3** (Scheme 1, Figure 1), which must be obtained through a different mechanism. Since the main difference between products **2** and **3** is just the proton which is transferred between nitrogen and carbon atoms (H1 and H2, respectively), as well as the nitrogen atom which is finally bonded to Mn1 (N1 and N2, respectively), it was assumed that the reaction path in mechanism *M3* leading to product **3** should sensibly be very similar to the one leading to product **2** in mechanism *M1*. Therefore, it seemed unnecessary to develop again the whole mechanism, and then only the most significant features for mechanism *M3* will be detailed here. Theoretically optimized geometries for the molecules involved in the partial mechanism *M3* are depicted in Figure 8, and selected bond distances and angles are collected in Table 5 and Table 6, respectively. Bearing in mind that the reaction path involved in the current mechanism is (**1 + Ag_2_O)**—**I11**—**TS(11-12)**—**I12**—**…**—(**3 + Ag_2_O)**—(**3 + Ag_14_O_7_**), the most relevant features from Figure 8 and Table 5 are the following. A comparison between intermediate **I11** (Figure 8) and intermediate **I1** (Figure 4) shows that the first step in both mechanisms is basically the same, with the formation of the N2-H2…O1 hydrogen bond in mechanism *M3* instead of the almost equivalent N1-H1…O1 hydrogen bond in mechanism *M1*. The stabilizing effect through a Ag1-phenyl attractive interaction in **I1** (Ag1-C* distance is 3.108 Å, with C* the centroid of the phenyl group) is likely to be substituted in **I11** by a similar Ag1-bipy stabilizing interaction (Ag1-C* distance is 3.068 Å, with C* the centroid of the closest pyridine group). Moreover, not only N1-H1 and H1-O1 bond distances in **I1** (1.080 Å and 1.582 Å, respectively; see Table 1) are not far from their equivalents N2-H2 and H2-O1 bond distances in **I11** (1.039 Å and 1.753 Å, respectively; see Table 5) but also N1-H1-O1 and N2-H2-O1 bond angles are quite similar (163.6° and 156.2°, respectively; see Tables S4 and S8 in Supplementary Material), not to mention the Ag1-O1-Ag2 bond angle in both intermediates (105.2° in **I1** and 104.9° in **I11**), among many other similarities. Thus, intermediates **I11** and **I12**, as well as the transition state **TS(11-12)**, are almost equivalent to **I2**, **I3**, and **TS(2-3)**, respectively, in the sense that the bipyridine group in the latter plays the same role as the phenyl group in the former. From **I11** to **I12** there is just a rotation of approximately 90° of the [Mn]^⊕^ group about the C1-Mn1-(CO)_ax_ axis, passing through the transition state **TS(11-12)**. Consequently, through this step there is only a slight increase in N2-H2 bond distances (from 1.039 Å in **I11** to 1.043 Å in **I12**) and a slight decrease in H2-O1 distances (from 1.753 Å in **I11** to 1.702 Å in **I12**), coming with an increase in N2-H1-O1 angles (from 156.2° in **I11** to 163.1° in **I12**) and a decrease in Ag1-O1-Ag2 bond angles (from 104.9° in **I11** to 100.2° in **I12**).

Calculated standard Gibbs free energies at room temperature for the above compounds along the path of the *M3* mechanism are collected in Table S11, from which the value $\Delta G_{298}^{0}\left(1\to 3\right)=-11.9{\;\mathrm{kcal}\;\mathrm{mol}}^{-1}$ is obtained. Consequently, *M3* is a thermodynamically possible mechanism, which may be also kinetically favorable and very likely to be observed experimentally. Considering all the results and data explained above, the experimentally observed fact that both products **2** and **3** are obtained within very similar amounts [13,14] is fully justified by our current calculations.

## 5. Conclusions

We have carried out DFT theoretical calculations that support a plausible mechanism for the experimentally observed Ag_2_O-catalyzed isomerization of diaminocarbenes to formamidines, coordinated to Mn(I). This mechanism involves metalation of an N-H residue of the carbene ligand by the catalyst Ag_2_O followed by a translocation process of the Mn(I) and Ag(I) ions between the carbene carbon atom and the nitrogen atom, which proceeds through a key transition state showing a μ-η^2^:η^2^ coordination of the formamidinyl bridging ligand between manganese and silver, and leading to two different products **2** and **3**, as it has been experimentally observed. Additional products **2′** and **2″** are also theoretically possible, although less stable thermodynamically. Calculations using Cu_2_O as a catalyst instead of Ag_2_O show a similar reaction mechanism thermodynamically possible, leading to the same **2** and **2′** products, but highly unfavorable kinetically to be observed, which fully agree with our experimental results.

## Figures and Tables

**Table 1:** Selected bond lengths (Å) of theoretically optimized structures (B3P86/6-31+G(d), LanL2DZ(Ag) level of theory) in mechanism *M1*. See Figure 1 for the atomic numbering scheme.

Complex	Mn1-C1	N1-H1	C1-H1	H1-O1	N1-Ag1	C1-Ag1	Mn1-N1
**1** + Ag_2_O	2.083	1.015	----	----	----	----	----
**I1**	2.102	1.080	----	1.582	----	----	----
**TS(1-2)**	2.138	1.259	----	1.260	----	----	----
**I2**	2.116	2.192	----	0.983	----	----	----
**TS(2-3)**	2.125	----	----	0.968	2.137	----	----
**I3**	2.129	----	----	0.967	2.134	----	----
**TS(3-4)**	3.341	----	----	0.978	2.440	2.603	2.330
**I4**	----	----	----	0.968	----	2.120	2.215
**TS(4-5)**	----	----	1.856	1.024	----	----	2.278
**I5**	----	----	1.106	1.806	----	----	2.164
**TS**(**5**-Products)	----	----	1.095	1.807	----	----	2.629
**2** + Ag_2_O			1.087	----	----	----	2.111
**2′** + Ag_2_O	----	----	1.092	----	----	----	2.127
**2″** + Ag_2_O	----	----	1.093	----	----	----	2.195

**Table 2:** Selected bond angles (°) of theoretically optimized structures (B3P86/6-31+G(d), LanL2DZ(Ag) level of theory) in mechanism *M1*.

Complex	Mn1-C1-N1	Mn1-C1-N2	Mn1-N1-C1	Mn1-N1-C2	Ag1-O1-Ag2
**1** + Ag_2_O	128.2	118.3	----	----	94.4
**I1**	128.8	116.9	----	----	105.2
**TS(1-2)**	130.0	114.6	----	----	115.4
**I2**	130.3	115.8	----	----	143.1
**TS(2-3)**	129.4	114.0	----	----	141.3
**I3**	129.4	113.9	----	----	128.0
**TS(3-4)**	34.0	98.7	126.7	108.4	128.0
**I4**	----	----	137.7	109.4	88.1
**TS(4-5)**	----	----	140.8	107.6	141.4
**I5**	----	----	136.9	113.2	112.0
**TS**(**5**-Products)	----	----	143.1	104.2	122.4
**2** + Ag_2_O	----	----	123.3	121.0	94.4
**2′** + Ag_2_O	----	----	129.7	118.3	94.4
**2″** + Ag_2_O	----	----	135.8	112.7	94.4

**Table 3:** Selected bond lengths (Å) of theoretically optimized structures (B3P86/6-31+G(d) level of theory) in mechanism *M2*.

Complex	Mn1-C1	N1-H1	C1-H1	H1-O1	N1-Cu1	C1-Cu1	Mn1-N1
**1** + Cu_2_O	2.083	1.015	----	----	----	----	----
**I6**	2.087	1.070	----	1.678	----	----	----
**TS(6-7)**	2.084	1.080	----	1.602	----	----	----
**I7**	2.100	2.067	----	0.996	----	----	----
**TS(7-8)**	2.143	----	----	0.976	1.912	----	----
**I8**	2.101	----	----	0.966	1.944	----	----
**TS(8-9)**	3.274	----	----	0.971	2.169	2.435	2.393
**I9**	----	----	----	0.966	----	1.932	2.182
**TS(9-10)**	----	----	2.297	0.969	----	----	2.436
**I10**	----	----	1.108	1.822	----	----	2.160
**TS**(**10**-Products)	----	----	1.089	2.096	----	----	2.199
**2** + Cu_2_O			1.087				2.111
**2′** + Cu_2_O	----	----	1.092	----	----	----	2.127
**2″** + Cu_2_O	----	----	1.093	----	----	----	2.195

**Table 4:** Selected bond angles (°) of theoretically optimized structures (B3P86/6-31+G(d) level of theory) in mechanism *M2*.

Complex	Mn1-C1-N1	Mn1-C1-N2	Mn1-N1-C1	Mn1-N1-C2	Cu1-O1-Cu2
**1** + Cu_2_O	128.2	118.3	----	----	93.7
**I6**	128.3	118.1	----	----	100.9
**TS(6-7)**	128.6	117.7	----	----	110.4
**I7**	129.9	116.1	----	----	136.9
**TS(7-8)**	130.4	113.4	----	----	128.3
**I8**	129.8	115.7	----	----	92.9
**TS(8-9)**	40.7	97.4	116.8	107.4	100.9
**I9**	----	----	139.8	109.1	96.1
**TS(9-10)**	----	----	131.3	107.3	135.7
**I10**	----	----	136.3	113.0	111.0
**TS**(**10**-Products)	----	----	135.9	114.2	102.8
**2** + Cu_2_O					
**2′** + Cu_2_O	----	----	129.7	118.3	93.7
**2″** + Cu_2_O	----	----	135.8	112.7	93.7

**Table 5:** Selected bond lengths (Å) of theoretically optimized structures (B3P86/6-31+G(d), LanL2DZ(Ag) level of theory) and X-ray experimental structure in (partial) mechanism *M3*.

Complex	Mn1-C1	N2-H2	C1-H2	H2-O1	Mn1-N1
**1** + Ag_2_O	2.083	1.006	----	----	----
**I11**	2.088	1.039	----	1.753	----
**TS(11-12)**	2.101	1.031	----	1.845	----
**I12**	2.099	1.043	----	1.702	----
**3** + Ag_2_O	----	----	1.086	----	2.098
**3** (*exp*)	----	----	1.03 (3)	----	2.084 (3)

**Table 6:** Selected bond angles (°) of theoretically optimized structures (B3P86/6-31+G(d), LanL2DZ(Ag) level of theory) and X-ray experimental structure in (partial) mechanism *M3*.

Complex	Mn1-C1-N1	Mn1-C1-N2	Mn1-N2-C1	Mn1-N2-C3	Ag1-O1-Ag2
**1** + Ag_2_O	128.2	118.3	----	----	94.4
**I11**	126.0	120.6	----	----	104.9
**TS(11-12)**	126.6	120.5	----	----	107.3
**I12**	128.1	118.6	----	----	100.2
**3** + Ag_2_O	----	----	121.7	122.3	94.4
**3** (*exp*)	----	----	123.0 (2)	119.6 (2)	----

## Data Availability

CIF file of compound **3**·${\mathrm{ClO}}_{4}^{-}$ can be downloaded at: https://www.ccdc.cam.ac.uk/structures/ (CCDC deposition number 2044950).

## Supplementary Materials

The following supporting information can be downloaded at: https://www.mdpi.com/article/10.3390/ma15020491/s1, Figure S1: Graphic representation of compounds in mechanism M1; Figure S2: Graphic representation of compounds in mechanism M2; Figure S3: Graphic representation of compounds in mechanism M3; Table S1: Crystallographic data of complex **3**; Table S2: Cartesian coordinates of all reactants, products, intermediates, and transition states for mechanisms M1, M2, and M3; Table S3: Bond lengths of theoretically optimized structures in mechanism M1; Table S4: Bond angles of theoretically optimized structures in mechanism M1; Table S5: Bond lengths of theoretically optimized structures in mechanism M2; Table S6: Bond angles of theoretically optimized structures in mechanism M2; Table S7: Bond lengths of theoretically optimized structures in mechanism M3; Table S8: Bond angles of theoretically optimized structures in mechanism M3; Table S9: Standard Gibbs energies at room temperature for mechanism M1; Table S10: Standard Gibbs energies at room temperature for mechanism M2; Table S11: Standard Gibbs energies at room temperature for mechanism M3. Additionally, checkpoint files for each compound (those of geometry optimization procedures, frequency and thermodynamic properties calculations, and electronic energy calculations, both in gas phase and with solvent) are available from the authors upon request.
