# Supplementary material for: Ag2O versus Cu2O in the Catalytic Isomerization of Coordinated Diaminocarbenes to Formamidines: A Theoretical Study
Source: Materials (Basel). 2022 Jan 10;15(2):491. doi: 10.3390/ma15020491 (PMC8778719; doi:10.3390/ma15020491)
Supplement: Supplementary file 1 [file materials-15-00491-s001.zip › materials-1513187-supplementary.pdf]

Supplementary Material

for

Ag_2O versus Cu_2O in the Catalytic Isomerization of Coordinated Diaminocarbenes to Formamidines: A Theoretical Study.

Juan F. Van der Maelen[1,*] and Javier Ruiz[2,*]

[1] Departamento de Química Física y Analítica, Facultad de Química, Universidad de Oviedo, E-33006 Oviedo, Spain.

[2] Departamento de Química Orgánica e Inorgánica, Facultad de Química, Universidad de Oviedo, E-33006 Oviedo, Spain.

* Correspondence: fvu@uniovi.es (J.F.V.M.); jruiz@uniovi.es (J. R.)

Table S1. Crystallographic data of complex **3**· ClO_4^- .

Crystal data	
Chemical formula	C_{21} H_{18} Mn N_4 O_3, Cl O_4
CCDC No.	2044950
M_r	528.78
Crystal system, space group	Monoclinic, *P 2₁/c*
Temperature (K)	100(2)
a, *b*, *c* (Å)	8.3541(4), 25.7610(16), 10.6548(5)
β (°)	109.177(2)
V (Å^3)	2165.8(2)
Z	4
Radiation type	Mo *Kα*
μ (mm^{-1})	0.786
Crystal size (mm)	0.12 × 0.08 × 0.04
Data collection	
Diffractometer	Bruker APEX-II CCD
Absorption correction	Multi-scan
T_{min}, T_{max}	0.891, 0.969
No. of measured, independent and	19035, 4435
R_{int}	0.0872
θ values (°)	θ_{max} = 26.42, θ_{min} = 1.58
$(\sin \theta/\lambda)_{max}$ (Å^{-1})	0.626
Range of *h*, *k*, *l*	*h* = −10→9, *k* = 0→32, *l* = 0→13
Refinement	
$R[F^2 > 2\sigma(F^2)]$, $wR(F^2)$, S	0.049, 0.088, 1.00
No. of reflections	4435
No. of parameters	316
No. of restraints	0
H-atom treatment	H-atom parameters constrained
$(\Delta/\sigma)_{max}$	0.000
$\Delta\rho_{max}$, $\Delta\rho_{min}$ (e Å^{-3})	0.45, −0.44

Table S2. Cartesian coordinates of all reactants, products, intermediates, and transition states for mechanisms M1, M2, and M3.

Reactant 1 (B3P86-D3/6-31+G(d) model)

Mn	-0.12237300	-0.41726500	-0.64178700
N	-2.01022500	-0.96596400	-0.00995100
C	-2.45559800	-2.22946400	0.10889300
H	-1.71531200	-3.01237400	-0.00741400
C	-3.78450100	-2.54031500	0.35964300
H	-4.08586900	-3.57827200	0.44221200
C	-4.70180500	-1.50163500	0.48927300
H	-5.75089100	-1.70591600	0.67495500
C	-4.25180400	-0.19310300	0.37210700
H	-4.94983700	0.63035800	0.46125300
C	-2.90005300	0.05013000	0.12449600
C	-2.31970900	1.39313300	-0.01351400
C	-3.04113900	2.57064800	0.18548100
H	-4.08999200	2.53418900	0.45370100
C	-2.40358200	3.79527700	0.03936200
H	-2.95072100	4.72030000	0.18632300
C	-1.05417500	3.80852100	-0.29705700
H	-0.50970400	4.73726400	-0.42362300
C	-0.39822500	2.59870300	-0.47996000
H	0.65091400	2.57334500	-0.74905100
N	-1.00726900	1.41074400	-0.34688200
C	0.55195100	-0.31558300	1.32686300
N	-0.34768500	-0.42986300	2.31487500
H	-1.29467200	-0.61080800	2.02760800
C	-0.09377900	-0.37918700	3.74587300
H	0.60310600	-1.16537400	4.05890000
H	-1.03429100	-0.53769500	4.27297800
H	0.29999200	0.59704400	4.05219700
N	1.80279200	-0.11343800	1.76650400
H	1.95452000	-0.04017000	2.76782100
C	3.01151700	-0.02713800	1.00429500
C	3.66637000	1.20084100	0.91290500
H	3.22521900	2.08173800	1.37046100
C	4.87529900	1.28602400	0.22784700
H	5.38392600	2.24139600	0.14931000
C	5.42859000	0.14799600	-0.35708100
H	6.36888700	0.21723900	-0.89421300
C	4.78080200	-1.08054700	-0.24232100
H	5.21792100	-1.97109400	-0.68210500
C	3.57638600	-1.17537000	0.45060100
H	3.07993600	-2.13260800	0.56765600
C	-0.78021200	-0.54134900	-2.34491300
O	-1.17563900	-0.64449200	-3.41969000
C	0.53656200	-2.08869300	-0.72317200
O	0.94211000	-3.16986400	-0.75151400
C	1.37121400	0.26700100	-1.40556800
O	2.22206300	0.72277700	-2.03634900

Reactant Ag2O (B3P86-D3/6-31+G(d),LanL2DZ(Ag) model)

Ag	0.00000000	1.48649800	-0.10802800
Ag	0.00000000	-1.48649800	-0.10802800
O	0.00000000	0.00000000	1.26933400

Reactant Cu2O (B3P86-D3/6-31+G(d) model)

Cu	0.00000000	1.29685000	-0.14742800
Cu	0.00000000	-1.29685000	-0.14742800
O	0.00000000	0.00000000	1.06885500

Product 2 (B3P86-D3/6-31+G(d) model)

Mn	-0.78449900	0.26362700	1.13965000
N	-1.90812000	-0.03715400	-0.54516100
C	-2.88845900	0.76552300	-0.98541200
H	-3.11371600	1.63132600	-0.37282300
C	-3.60351900	0.51839000	-2.14875500
H	-4.38942300	1.20097000	-2.45217700
C	-3.29269300	-0.61304000	-2.89363700
H	-3.82939900	-0.84277200	-3.80837700
C	-2.28690500	-1.45560600	-2.44046200
H	-2.03633400	-2.34868100	-3.00045700
C	-1.61206900	-1.14820700	-1.25994700
C	-0.55886500	-1.99034800	-0.67656600
C	-0.13588400	-3.19663600	-1.23094400
H	-0.57735600	-3.56531600	-2.14910200
C	0.85368100	-3.93170600	-0.59214100
H	1.19081300	-4.87501300	-1.00943300
C	1.39462600	-3.44006800	0.58923600
H	2.16652300	-3.97748200	1.12828300
C	0.92480400	-2.23224000	1.08541900
H	1.32339400	-1.82122700	2.00501000
N	-0.02941100	-1.51551500	0.47486600
C	0.44241400	2.39390600	-0.63040100
N	1.30454200	3.09256800	-1.37252100
H	2.21861800	2.68616400	-1.53501600
C	1.04491200	4.41904000	-1.89787500
H	0.03083900	4.72294200	-1.62964300
H	1.13370000	4.42807300	-2.98818800
H	1.74490000	5.14707300	-1.47724300
N	0.63661000	1.22255600	-0.09170900
H	-0.51403700	2.88538800	-0.46832000
C	1.92354900	0.63835700	-0.32559400
C	2.93333200	0.75147000	0.63415300
H	2.74135500	1.29602700	1.55324100
C	4.17843700	0.16965300	0.40637200
H	4.95736400	0.26385300	1.15710000
C	4.42739000	-0.52082100	-0.77739500
H	5.39996000	-0.97025100	-0.95235300
C	3.42556600	-0.62379800	-1.74007800
H	3.61521700	-1.15247100	-2.66969800
C	2.17803700	-0.04545200	-1.51907000
H	1.39429500	-0.11799300	-2.26826700
C	-1.99648400	-0.61735100	2.12425200
O	-2.77675400	-1.19634000	2.74003300
C	-1.53817300	1.82298800	1.56283000
O	-2.03680200	2.83777800	1.80080300
C	0.30149800	0.40856500	2.55769800
O	0.99346300	0.48929100	3.47638900

Product 2' (B3P86-D3/6-31+G(d) model)

Mn	0.65342000	-0.28583600	1.10907100
N	1.65057600	0.88716400	-0.24861600
C	2.93033700	0.74186000	-0.62307200
H	3.48774600	-0.05017000	-0.13548300
C	3.54432500	1.56040800	-1.55938900
H	4.58441800	1.39655600	-1.81807400
C	2.80074900	2.58201900	-2.13869100
H	3.24236200	3.24257800	-2.87773800
C	1.48312400	2.75726200	-1.73957300
H	0.89278500	3.56175300	-2.16136200

C	0.93346000	1.90268500	-0.78443800
C	-0.42459700	2.04968900	-0.24355000
C	-1.30525400	3.05898900	-0.62704800
H	-1.01944700	3.78411300	-1.37956300
C	-2.55631900	3.13543900	-0.02995500
H	-3.25249600	3.91741800	-0.31530000
C	-2.89361400	2.19990500	0.93990700
H	-3.85566400	2.21975100	1.43903200
C	-1.96794300	1.22091500	1.27204400
H	-2.19911700	0.47336600	2.02112200
N	-0.75988500	1.13812800	0.69781500
C	0.44462300	-2.08348400	-1.43770100
N	1.74345400	-2.38874200	-1.49941700
H	2.33074300	-2.15700900	-0.71115400
C	2.29235100	-3.32380400	-2.46656400
H	1.55971300	-3.49772600	-3.25708600
H	2.53592200	-4.28279700	-1.99816700
H	3.19586000	-2.90975500	-2.92152900
N	-0.15880400	-1.35868800	-0.53791700
H	-0.14540000	-2.50054200	-2.25601300
C	-1.57167000	-1.25444900	-0.78849800
C	-2.48121500	-1.94344500	0.01610500
H	-2.11933300	-2.57229200	0.82254500
C	-3.84712200	-1.84686800	-0.23711200
H	-4.54536300	-2.39728600	0.38644000
C	-4.31582400	-1.06835600	-1.29301200
H	-5.38114500	-1.00073900	-1.49051000
C	-3.40856900	-0.39084400	-2.10328800
H	-3.76275100	0.20635200	-2.93852600
C	-2.04121900	-0.48257900	-1.85357400
H	-1.32982400	0.04295100	-2.48544700
C	1.26903500	0.75074200	2.43726800
O	1.66314600	1.43581200	3.27216100
C	1.99485700	-1.41529100	1.38458200
O	2.90493300	-2.11096700	1.55991100
C	-0.34197500	-1.21994300	2.27464300
O	-0.95647100	-1.80744100	3.05198800

Product 2'' (B3P86-D3/6-31+G(d) model)

Mn	0.61269700	-0.61750100	0.90210700
N	1.76836500	0.74479500	-0.10463400
C	3.05545400	0.58314500	-0.44389300
H	3.52479200	-0.33700200	-0.11551800
C	3.77890100	1.53321000	-1.14979800
H	4.81913200	1.34705100	-1.39241000
C	3.14238000	2.71131300	-1.52290600
H	3.67003600	3.47679100	-2.08257600
C	1.82213700	2.90623900	-1.14124900
H	1.31689900	3.83095800	-1.39332700
C	1.16165900	1.91442500	-0.41716900
C	-0.19256800	2.06904000	0.13170000
C	-0.95167300	3.23083700	0.00523000
H	-0.57629900	4.07607900	-0.55925600
C	-2.19122000	3.30652500	0.62644900
H	-2.79103900	4.20705400	0.54320100
C	-2.63975900	2.21597100	1.36111200
H	-3.59613100	2.22806900	1.87121300
C	-1.83643600	1.08693500	1.43521900
H	-2.16129900	0.21290600	1.98660000
N	-0.64105900	1.00436300	0.83487500
C	0.14655300	-1.41268500	-2.22512700

N	1.33726500	-1.85804900	-2.65060100
H	1.40139100	-1.92486900	-3.65542700
C	2.33676100	-2.60791800	-1.91523200
H	1.86123400	-3.26601600	-1.18688500
H	3.05262600	-1.96104100	-1.40571600
H	2.88779400	-3.22491900	-2.62710100
N	-0.29156000	-1.17393600	-1.01933300
H	-0.54607700	-1.24940200	-3.05423200
C	-1.71694700	-0.93969600	-1.05333700
C	-2.58096400	-1.85207100	-0.44698300
H	-2.17746300	-2.73458900	0.03750800
C	-3.95785900	-1.64580900	-0.49125400
H	-4.61957800	-2.36698200	-0.02096000
C	-4.48514300	-0.53915500	-1.15203500
H	-5.55912300	-0.38612200	-1.19080800
C	-3.62503200	0.36002300	-1.77757400
H	-4.02469500	1.21943700	-2.30806800
C	-2.24773800	0.16217300	-1.72952000
H	-1.57700900	0.86758700	-2.21373200
C	1.25231700	0.02729400	2.43848500
O	1.66756400	0.47358700	3.41408000
C	1.83544300	-1.91451700	1.01170000
O	2.64513200	-2.71644300	1.19954700
C	-0.50653100	-1.68126900	1.81642300
O	-1.19170800	-2.34236400	2.46527500

Product 3 (B3P86-D3/6-31+G(d) model)

Mn	-1.26915200	-1.02434500	0.47170200
N	-2.42629800	0.17912400	-0.70972300
C	-3.31819500	-0.24017200	-1.62048000
H	-3.45029900	-1.31305600	-1.70651300
C	-4.04946000	0.62859200	-2.41800800
H	-4.75954100	0.23125800	-3.13457400
C	-3.84944600	1.99619000	-2.27048700
H	-4.39943000	2.70818500	-2.87721700
C	-2.94077900	2.44012300	-1.31918400
H	-2.78343600	3.50252600	-1.17637500
C	-2.24780300	1.51087200	-0.54482600
C	-1.32170400	1.87533500	0.53567000
C	-1.02481600	3.18991000	0.89207000
H	-1.44777900	4.01898300	0.33747600
C	-0.18777800	3.43388000	1.97242100
H	0.04968800	4.45209400	2.26284000
C	0.32542000	2.35212300	2.67825800
H	0.97025500	2.48892600	3.53907700
C	-0.00560200	1.06988900	2.26355600
H	0.37189200	0.20308300	2.79479700
N	-0.80026500	0.82385000	1.21069200
N	0.26091300	-0.73932000	-0.93504600
C	0.04998100	-0.92577300	-2.36521000
H	0.22695900	0.00358200	-2.92504200
H	-0.97763500	-1.22906700	-2.55780000
H	0.70698400	-1.70900800	-2.76780300
C	1.47691700	-0.46858500	-0.57296500
H	1.69603800	-0.35015400	0.48382200
N	2.54270000	-0.30394900	-1.37694900
H	2.40253500	-0.29861900	-2.37869500
C	3.87114700	-0.10662700	-0.91903500
C	4.72392700	0.70741400	-1.66515400
H	4.36267200	1.18954900	-2.57000800
C	6.03163600	0.90736000	-1.23790500

H	6.69402000	1.53729000	-1.82345200
C	6.48653000	0.31325000	-0.06279900
H	7.50698900	0.47417600	0.26954300
C	5.63103600	-0.50077200	0.67459700
H	5.98506900	-0.98718200	1.57827500
C	4.32691600	-0.72756800	0.24478900
H	3.68577800	-1.41334800	0.79116600
C	-1.72918000	-2.55681700	-0.33586100
O	-2.03187200	-3.52751000	-0.87853300
C	-0.14938300	-1.92160900	1.53518500
O	0.60758900	-2.46788500	2.21421300
C	-2.60489700	-1.19793500	1.65801400
O	-3.46548000	-1.29123100	2.41547700

Intermediate I1 (B3P86-D3/6-31+G(d),LanL2DZ(Ag) model)

Mn	-2.43757700	0.90719500	-0.26990000
N	-3.57762900	-0.72898600	-0.88351000
C	-4.09180100	-0.89612500	-2.12403900
H	-3.75549000	-0.18818200	-2.87844900
C	-5.00401900	-1.90539200	-2.43436500
H	-5.38724200	-1.98981700	-3.45072900
C	-5.41264800	-2.78121700	-1.41915100
H	-6.13489000	-3.57267300	-1.62068700
C	-4.88195700	-2.62109700	-0.13724500
H	-5.19266100	-3.28371900	0.66818000
C	-3.95973100	-1.59107100	0.10858300
C	-3.33709100	-1.33571300	1.41625700
C	-3.48474600	-2.18021500	2.52844300
H	-4.09361900	-3.07946800	2.45900700
C	-2.83841400	-1.86260200	3.72462000
H	-2.94619100	-2.50727800	4.59724900
C	-2.05014500	-0.70593300	3.77646300
H	-1.52304000	-0.41457000	4.68440500
C	-1.94506000	0.08930800	2.63415800
H	-1.34394400	0.99525100	2.63412500
N	-2.57554400	-0.20401600	1.47689500
C	-0.64513900	-0.18058500	-0.56240500
N	-0.74687700	-1.47317500	-0.92595900
H	-1.68094900	-1.81168100	-1.10432200
C	0.35327500	-2.42562800	-1.13321100
H	1.08410700	-2.01921000	-1.84622500
H	-0.07423100	-3.35806500	-1.52263400
H	0.88734900	-2.62008400	-0.19140200
N	0.59838300	0.24546600	-0.31373800
H	1.46239300	-0.40955000	-0.30298000
C	0.99180600	1.59666200	-0.10693600
C	1.61141300	1.93850400	1.11272300
H	1.65635200	1.19386500	1.90834200
C	2.13537600	3.22921900	1.29454500
H	2.59514100	3.49698700	2.24729300
C	2.04080000	4.17779700	0.26183100
H	2.43934000	5.18270400	0.40553900
C	1.42547400	3.83033900	-0.94931000
H	1.35650000	4.56131400	-1.75611900
C	0.91589700	2.53854600	-1.14197100
H	0.47550200	2.25039700	-2.09482700
C	-4.03537800	1.75286300	-0.02384900
O	-5.05116100	2.29955900	0.10865100
C	-2.21351300	1.62132700	-1.89445100
O	-2.05936800	2.06316200	-2.96305300
C	-1.71492300	2.35341200	0.51975800

O	-1.41288900	3.32697000	1.08520800
Ag	4.25476400	-2.54727400	-0.25806700
O	2.92406500	-0.98577000	-0.21524600
Ag	3.97639800	0.81393600	0.07953000

Intermediate I2 (B3P86-D3/6-31+G(d),LanL2DZ(Ag) model)

Mn	-2.33960500	0.87179900	-0.35739300
N	-3.49740600	-0.68774100	-1.03271700
C	-3.82408900	-0.91359100	-2.31515600
H	-3.33680800	-0.28284900	-3.04969800
C	-4.72856100	-1.88858600	-2.70158500
H	-4.95480000	-2.02349500	-3.75352300
C	-5.33217100	-2.66635600	-1.71967500
H	-6.05276400	-3.43425700	-1.98242200
C	-5.00287600	-2.43946800	-0.39282000
H	-5.47046600	-3.02578700	0.38931300
C	-4.07964600	-1.44518000	-0.07388800
C	-3.67293100	-1.11329900	1.29375600
C	-4.08266000	-1.83052500	2.41594400
H	-4.73041900	-2.69300300	2.31150400
C	-3.65281600	-1.43508300	3.67207800
H	-3.96606400	-1.97955400	4.55713500
C	-2.81620800	-0.33016600	3.77073500
H	-2.45340800	0.02375100	4.72952600
C	-2.44375500	0.33190200	2.61242900
H	-1.79130900	1.19562000	2.65305600
N	-2.85910800	-0.03869300	1.39387300
C	-0.61757600	-0.35557000	-0.29304000
N	-0.78689900	-1.63494700	-0.71941400
H	-1.67597200	-1.88025800	-1.11728700
C	0.25305300	-2.63661800	-0.74124000
H	1.07950600	-2.34739900	-1.40208600
H	-0.16953400	-3.57705200	-1.10280100
H	0.65857100	-2.79622700	0.26476800
N	0.58070600	-0.10839200	0.16770600
H	2.52598000	-1.01847000	-0.27326400
C	1.07574900	1.12218700	0.52082000
C	1.45316400	1.39364900	1.84761400
H	1.23461700	0.64418100	2.60270700
C	2.09321500	2.57819700	2.19216000
H	2.35294600	2.76003800	3.23139600
C	2.41643700	3.53077600	1.22726400
H	2.91720300	4.45246900	1.50381400
C	2.05779600	3.29517800	-0.10216800
H	2.22724400	4.05732700	-0.85853300
C	1.38366700	2.10962100	-0.46081100
H	1.00282900	1.97875000	-1.47109100
C	-3.84223500	1.88206600	-0.47201200
O	-4.78126900	2.53763500	-0.58060100
C	-1.73496600	1.39019100	-1.95335000
O	-1.30665500	1.70440300	-2.97973500
C	-1.59411200	2.30785800	0.42406300
O	-1.26855700	3.29458200	0.92684800
Ag	4.48447700	-2.57386100	0.28191000
O	3.46646300	-0.96783600	-0.55452100
Ag	3.52192600	1.20293000	-0.79183500

Intermediate I3 (B3P86-D3/6-31+G(d),LanL2DZ(Ag) model)

Mn	-2.49325100	0.79274300	-0.51219500
N	-3.62193900	-0.89888400	-0.85121900
C	-4.02127400	-1.33725200	-2.05612000

H	-3.58780100	-0.83521200	-2.91351800
C	-4.93356400	-2.36661700	-2.21868300
H	-5.22041300	-2.67627300	-3.21759700
C	-5.46704400	-2.97096800	-1.08592300
H	-6.19264700	-3.77343400	-1.17264900
C	-5.06090200	-2.52605600	0.16242300
H	-5.47411600	-2.97427700	1.05827800
C	-4.13290700	-1.49065700	0.25471500
C	-3.64689300	-0.93597000	1.52147000
C	-3.96133500	-1.47049800	2.76909500
H	-4.59095600	-2.34898500	2.84895800
C	-3.45806600	-0.87181400	3.91289200
H	-3.69596000	-1.27254400	4.89304600
C	-2.64599500	0.24705000	3.77583100
H	-2.22812700	0.75464500	4.63827800
C	-2.37060000	0.72165200	2.50356400
H	-1.74103100	1.59149300	2.35887400
N	-2.85806000	0.15397200	1.39264900
C	-0.69170500	-0.33655600	-0.39939600
N	-0.86689400	-1.67063300	-0.56955300
H	-1.80088000	-1.95261100	-0.80900100
C	0.11808600	-2.72691700	-0.55680400
H	0.88292900	-2.58835300	-1.33287000
H	-0.39160700	-3.67261700	-0.75604100
H	0.61403700	-2.81457400	0.41824700
N	0.53534600	0.07032900	-0.15467400
H	4.48035700	-2.57058300	-0.00930600
C	0.87390700	1.44144700	-0.02728400
C	1.16669500	1.97240900	1.23308900
H	1.06791300	1.33263500	2.10659300
C	1.58114500	3.29247900	1.36100000
H	1.79176800	3.69773400	2.34682500
C	1.72330400	4.09600300	0.23237600
H	2.04212800	5.12880000	0.33332200
C	1.45575000	3.56468900	-1.02584000
H	1.56843200	4.18267000	-1.91216000
C	1.03965800	2.24456900	-1.15912800
H	0.84340400	1.82290500	-2.14025400
C	-4.05935600	1.68809600	-0.67478700
O	-5.04550300	2.26364700	-0.81377600
C	-2.04413000	1.09374300	-2.21706400
O	-1.73424700	1.26275400	-3.31567400
C	-1.82233700	2.39171300	-0.00764100
O	-1.60647700	3.47297300	0.32585400
Ag	6.20245700	-0.62458000	-0.05454600
O	4.40117300	-1.64130300	0.24657600
Ag	2.42882900	-0.89927800	0.01230900

Intermediate I4 (B3P86-D3/6-31+G(d),LanL2DZ(Ag) model)

Mn	2.30575800	-1.00164100	-0.64006100
N	3.11879800	0.75215700	0.10330600
C	4.01144100	1.53792500	-0.52852900
H	4.35628200	1.18194300	-1.49675600
C	4.46478100	2.74610700	0.00338500
H	5.19087800	3.34041400	-0.55029300
C	3.96320500	3.16350000	1.24196100
H	4.27667600	4.11064900	1.68170500
C	3.08004800	2.32600900	1.92850600
H	2.71554000	2.60697700	2.91445800
C	2.69617200	1.10802100	1.35054600
C	1.93542400	0.06784000	2.05906000

C	1.45911200	0.19597900	3.37303800
H	1.53525400	1.14690600	3.89655400
C	0.90798900	-0.91618000	4.01600900
H	0.54347100	-0.83252800	5.04021100
C	0.85607000	-2.13676500	3.33234300
H	0.46498400	-3.03972400	3.79968100
C	1.30400000	-2.18179000	2.01023900
H	1.24866300	-3.09946600	1.42940300
N	1.81036200	-1.10583700	1.37565200
C	0.02307000	1.38230100	-0.74738300
N	0.85181200	2.38761800	-1.16682500
H	0.41288900	3.29497000	-1.05717200
C	1.71508900	2.33825700	-2.35696700
H	2.19358500	1.36570000	-2.42205700
H	2.48611300	3.11583000	-2.27970900
H	1.12290000	2.50068700	-3.27317100
N	0.39199300	0.10594400	-0.79031400
H	-4.67002800	2.33005400	-0.30659600
C	-0.69286400	-0.79580100	-0.54545000
C	-1.29021300	-1.45873500	-1.63617900
H	-0.87816600	-1.30086400	-2.63279300
C	-2.40365800	-2.28058200	-1.44974900
H	-2.86016400	-2.78308800	-2.30295800
C	-2.94371900	-2.46781800	-0.15121500
H	-3.74501800	-3.19065300	0.01301500
C	-2.31443300	-1.83344400	0.95269700
H	-2.68112900	-2.01122900	1.96484800
C	-1.20809500	-0.99245900	0.74797800
H	-0.76987800	-0.44844000	1.58060300
C	3.86465300	-1.84606100	-0.35559700
O	4.87837900	-2.38429200	-0.17554700
C	2.72286200	-0.82246000	-2.37790100
O	3.01198100	-0.78249100	-3.50565400
C	1.56375700	-2.58224400	-1.07178800
O	1.15200000	-3.63863800	-1.33588300
Ag	-4.26322800	-0.33572000	0.02486600
O	-4.11311700	1.83212600	0.31976400
Ag	-1.98187100	1.91551700	-0.19171000

Intermediate I5 (B3P86-D3/6-31+G(d),LanL2DZ(Ag) model)

Mn	2.75161500	-0.30240500	-0.79203200
N	2.85933500	1.57148100	0.03069200
C	3.26818500	2.70428100	-0.61308900
H	3.58092800	2.58451900	-1.64667400
C	3.28260900	3.96456400	0.01531100
H	3.62302500	4.83839800	-0.53869100
C	2.85179700	4.06287200	1.35888400
H	2.83291900	5.02644800	1.86831300
C	2.48058100	2.88720700	2.04393900
H	2.19121100	2.93238200	3.09209200
C	2.51985500	1.64599600	1.36855100
C	2.34521100	0.33905800	2.02756500
C	2.05838700	0.17192800	3.40199100
H	1.85250500	1.03685700	4.02970600
C	2.06364400	-1.12455300	3.95934500
H	1.85346500	-1.26790900	5.01937400
C	2.35962600	-2.22686500	3.12615200
H	2.39117800	-3.24335200	3.51538100
C	2.60136500	-2.00025700	1.75639200
H	2.80297800	-2.82635500	1.08068300
N	2.58833100	-0.74855900	1.21135800

C	-0.28605400	0.85738400	-0.59965700
N	-0.24381900	2.12592200	-1.08450700
H	-1.11916700	2.61568600	-0.89817300
C	0.58466000	2.70641400	-2.15734400
H	1.38512400	2.01677900	-2.41876700
H	1.01861700	3.66565900	-1.83834100
H	-0.03709400	2.87441200	-3.05275800
N	0.60776900	-0.13614100	-0.65287600
H	-1.28275800	0.67660100	-0.12918000
C	-0.02658900	-1.39527800	-0.26207500
C	-0.37895100	-2.32121100	-1.27519400
H	-0.10062400	-2.11220000	-2.30836700
C	-1.10233700	-3.49311400	-0.94487900
H	-1.36516100	-4.20439600	-1.72935000
C	-1.47881100	-3.74393600	0.40334700
H	-2.03097800	-4.64940100	0.65904000
C	-1.11703700	-2.81615400	1.41414600
H	-1.40760800	-2.99608300	2.45043300
C	-0.39795200	-1.64492700	1.08139100
H	-0.16497300	-0.90520400	1.84641400
C	4.54062400	-0.39887900	-0.71510800
O	5.72786700	-0.43736300	-0.65047600
C	2.81605300	0.16258700	-2.51895100
O	2.88562900	0.42434800	-3.68137400
C	2.69992800	-2.01361100	-1.33906900
O	2.74318100	-3.14628100	-1.70535500
Ag	-4.82725000	1.72720200	0.27883100
O	-3.01859000	0.79950300	-0.03089200
Ag	-3.30697800	-1.24325200	-0.41453700

Intermediate I6 (B3P86-D3/6-31+G(d) model)

Mn	-1.73298400	0.96203900	-0.30628400
N	-3.03530000	-0.44459900	-1.06665000
C	-3.28032500	-0.65607600	-2.36750100
H	-2.65923100	-0.11158100	-3.06870700
C	-4.27037500	-1.51408900	-2.81865400
H	-4.42381000	-1.64223000	-3.88436600
C	-5.04942200	-2.18439300	-1.88366100
H	-5.84096400	-2.85697100	-2.19777200
C	-4.79929900	-1.97859100	-0.53580700
H	-5.39725400	-2.48934100	0.20938700
C	-3.78308900	-1.10551700	-0.15149300
C	-3.43028800	-0.82671200	1.24632100
C	-4.03486300	-1.45609000	2.33226900
H	-4.81672900	-2.19024200	2.17887700
C	-3.62894200	-1.13650300	3.61842600
H	-4.09190800	-1.61503600	4.47519000
C	-2.62151200	-0.19535800	3.78389000
H	-2.26615100	0.09071600	4.76755100
C	-2.06597300	0.39142800	2.65778100
H	-1.28483800	1.13557600	2.75366000
N	-2.45293300	0.09226200	1.41203200
C	-0.18112300	-0.43369900	-0.28366500
N	-0.49981700	-1.73232600	-0.31280400
H	-1.47695600	-1.95147500	-0.41433500
C	0.42308600	-2.85835500	-0.29166400
H	1.07486800	-2.84872100	-1.17097400
H	-0.16012900	-3.77990300	-0.29150200
H	1.06494500	-2.83921300	0.59400300
N	1.13023500	-0.21105100	-0.18869700
H	1.84841800	-0.99477500	-0.06946900

C	1.83965700	1.01687300	-0.18646600
C	2.50804300	1.37145000	1.01128600
H	2.17311000	0.93688000	1.95041800
C	3.51709400	2.34564800	0.98503400
H	4.00109300	2.64950100	1.90884100
C	3.79974500	3.02195500	-0.21386200
H	4.56821800	3.78793900	-0.23193900
C	3.07237700	2.73259200	-1.35691900
H	3.27530200	3.26787900	-2.27909400
C	2.11460000	1.71066400	-1.35290900
H	1.62887500	1.41729300	-2.27681400
C	-3.11120000	2.14258900	-0.33241400
O	-3.96925700	2.90312600	-0.36662900
C	-1.16664200	1.52944500	-1.89889700
O	-0.82425100	1.87505400	-2.94371200
C	-0.77016800	2.20604200	0.55013800
O	-0.24661700	3.05062400	1.13218900
O	3.32743700	-1.68233200	0.32647400
Cu	4.04401800	-0.01901200	0.69556400
Cu	4.69134900	-2.45888000	-0.50442600

Intermediate I7 (B3P86-D3/6-31+G(d) model)

Mn	1.75093600	-0.76839300	-0.62737900
N	3.04030300	0.79286300	-0.90604700
C	3.28517700	1.40565800	-2.08914900
H	2.65326900	1.11934900	-2.91784900
C	4.29280600	2.34819800	-2.24541400
H	4.45133200	2.80674100	-3.21246100
C	5.08871700	2.67560000	-1.14660200
H	5.88902600	3.39882600	-1.23984400
C	4.84325900	2.05298400	0.07252400
H	5.45738100	2.28663600	0.93184900
C	3.81171600	1.11596900	0.17494600
C	3.47191200	0.39147900	1.39801600
C	4.07085800	0.62792500	2.63758700
H	4.83293400	1.38875600	2.74205800
C	3.67973100	-0.11929900	3.74269200
H	4.13755400	0.04999900	4.70906000
C	2.68980700	-1.08835200	3.58092600
H	2.35513200	-1.69457700	4.41233700
C	2.12813800	-1.27733900	2.32484700
H	1.35961800	-2.01926400	2.16537600
N	2.50660900	-0.56035400	1.24350700
C	0.21146200	0.51690200	-0.00445500
N	0.53187700	1.84347200	0.07645200
H	1.44565200	2.12372400	-0.22774000
C	-0.38436700	2.87980900	0.52415500
H	-1.23627300	2.98311000	-0.15925700
H	0.14847800	3.83142800	0.56215800
H	-0.77326900	2.64697300	1.52019800
N	-1.01933600	0.23928600	0.37320900
H	-2.80210400	1.28491400	0.32637600
C	-1.70933500	-0.95920000	0.30469500
C	-2.14374700	-1.59783800	1.48326200
H	-1.75182100	-1.24215700	2.42909300
C	-3.10388400	-2.60881600	1.44885400
H	-3.42454600	-3.07438900	2.37370200
C	-3.67526200	-3.01470700	0.23767300
H	-4.43258700	-3.78807900	0.21470700
C	-3.21540300	-2.44212500	-0.96932400
H	-3.53677200	-2.85279100	-1.92054500

C	-2.20553200	-1.44678000	-0.94128200
H	-1.79128300	-1.05639700	-1.86440800
C	3.09856600	-1.82014500	-1.20630600
O	3.96247600	-2.49066700	-1.61605700
C	0.98314000	-0.65540300	-2.22255800
O	0.44162600	-0.55005600	-3.25904900
C	0.85542000	-2.26915600	-0.25498900
O	0.39161600	-3.32324600	-0.03679900
O	-3.77076300	1.29834400	0.09431400
Cu	-4.06673600	-0.44788700	-0.63724400
Cu	-4.78878200	2.75971400	0.52586300

Intermediate I8 (B3P86-D3/6-31+G(d) model)

Mn	-1.49533700	1.03847300	0.05438700
N	-3.02289400	0.16849200	-0.99854600
C	-3.36345300	0.46006500	-2.27553300
H	-2.69398800	1.11274700	-2.81701000
C	-4.51314600	-0.04023100	-2.87347100
H	-4.74299100	0.22651800	-3.89657800
C	-5.35344900	-0.87227300	-2.13364400
H	-6.26242300	-1.26804500	-2.56886000
C	-5.00750400	-1.18543900	-0.82312100
H	-5.65063400	-1.82489600	-0.23337500
C	-3.83570300	-0.65941900	-0.27393900
C	-3.37160900	-0.92519100	1.08861400
C	-4.00708800	-1.79959300	1.97288200
H	-4.90286800	-2.32730800	1.67405700
C	-3.48181200	-1.99088500	3.24632100
H	-3.96622600	-2.66274500	3.94362200
C	-2.32425900	-1.30187300	3.60545200
H	-1.88107400	-1.41866400	4.58569900
C	-1.73631400	-0.44436900	2.68397200
H	-0.84388800	0.10985500	2.93428700
N	-2.24088000	-0.24973000	1.44593500
C	-0.15365500	-0.47253700	-0.51978600
N	-0.71942600	-1.62203500	-0.97704400
H	-1.71957000	-1.62777200	-1.06413200
C	-0.02419200	-2.83005100	-1.39674300
H	0.65642100	-2.63616500	-2.23648200
H	-0.76285600	-3.56341900	-1.72420000
H	0.55091300	-3.27360400	-0.57345100
N	1.17641300	-0.50396000	-0.44951800
H	5.07505500	-2.68693700	-0.92207800
C	2.03906700	0.57685300	-0.11365400
C	2.78284300	0.50128400	1.09521500
H	2.45438400	-0.18768500	1.86620900
C	3.87874400	1.36875900	1.31933200
H	4.36379600	1.38554200	2.29067900
C	4.19521300	2.35575200	0.35528400
H	5.02405600	3.03088300	0.52801300
C	3.40801600	2.47534000	-0.78740400
H	3.63039500	3.24510900	-1.51675100
C	2.34474800	1.59372400	-1.02402500
H	1.79043000	1.65582600	-1.95185000
C	-2.68203600	2.31535700	0.53116600
O	-3.44146400	3.15162400	0.82026400
C	-0.91211000	2.00699800	-1.31674100
O	-0.55542200	2.62968600	-2.24334500
C	-0.32394600	1.78902600	1.17174900
O	0.35399700	2.32963000	1.95971500
O	4.44750800	-2.01842600	-0.61861600

Cu	2.58327900	-1.79233300	-0.82546900
Cu	4.62792500	-0.41692000	0.36481500

Intermediate I9 (B3P86-D3/6-31+G(d) model)

Mn	1.70125300	-0.76803900	-0.90370300
N	2.75902200	0.63268500	0.14038600
C	3.68041100	1.47552700	-0.37108500
H	3.87303100	1.39248900	-1.43128600
C	4.36496700	2.39899400	0.40998100
H	5.10384000	3.04427400	-0.04656700
C	4.07829800	2.47095100	1.77182700
H	4.57931300	3.19171300	2.40577900
C	3.16217100	1.57456700	2.31435600
H	2.96251300	1.58603800	3.37737200
C	2.53492200	0.64446100	1.48482600
C	1.72780300	-0.47377600	1.97594300
C	1.45497500	-0.70967300	3.32511200
H	1.73435600	0.01613800	4.07674700
C	0.84230700	-1.90082900	3.70416700
H	0.63749900	-2.10109500	4.74839100
C	0.52298600	-2.83771000	2.72264600
H	0.07622200	-3.79021700	2.97492800
C	0.78341700	-2.53022600	1.39221700
H	0.53016500	-3.22373700	0.60316500
N	1.35858500	-1.36795800	1.01614400
C	-0.47973100	1.69235400	-0.39301800
N	0.30682100	2.77547900	-0.63514000
H	-0.09686200	3.63634200	-0.29792900
C	1.32176800	2.97300500	-1.66763300
H	1.66837000	2.01539000	-2.03102100
H	2.17047900	3.53712700	-1.27556100
H	0.89704100	3.52363000	-2.51369600
N	-0.10969200	0.42858900	-0.68227600
H	-4.81191800	2.56277300	0.65876200
C	-1.24654000	-0.45106000	-0.58397400
C	-1.94797500	-0.80237600	-1.75428400
H	-1.55907300	-0.47163200	-2.71025300
C	-3.14624600	-1.50343400	-1.68733400
H	-3.68278800	-1.74700700	-2.59641900
C	-3.67316700	-1.90967000	-0.43689800
H	-4.53722000	-2.56384200	-0.39741000
C	-2.93647100	-1.61935000	0.73975600
H	-3.25257100	-2.02351500	1.69537900
C	-1.73927100	-0.87869400	0.65495600
H	-1.21940400	-0.59360100	1.56055900
C	3.19691600	-1.74883200	-0.95802000
O	4.18361600	-2.36953100	-0.97828800
C	1.99452400	-0.15674900	-2.56386500
O	2.18023400	0.18255400	-3.66669500
C	0.80815600	-2.13880800	-1.64279400
O	0.31192600	-3.07914700	-2.12859800
O	-4.13142200	1.88201600	0.57557600
Cu	-2.28971400	2.01699400	0.20112800
Cu	-4.26158300	0.03779900	0.27751400

Intermediate I10 (B3P86-D3/6-31+G(d) model)

Mn	2.18055300	-0.22930600	-0.84359600
N	2.43794800	1.26750200	0.52214300
C	2.98792700	2.47633400	0.28111600
H	3.36905800	2.64065600	-0.71681900
C	3.09238500	3.45993300	1.25716400

H	3.54419200	4.41234700	1.01344200
C	2.61126100	3.18981200	2.53705300
H	2.66074200	3.93932500	3.31678000
C	2.09596500	1.92610000	2.80860200
H	1.75668000	1.68633000	3.80728000
C	2.04086100	0.97112800	1.79198200
C	1.68175900	-0.43254500	2.00520800
C	1.36432600	-0.97639600	3.25148900
H	1.28249800	-0.34171700	4.12350100
C	1.17419700	-2.35026500	3.37305400
H	0.93922400	-2.78549100	4.33633500
C	1.31123500	-3.15366200	2.24264100
H	1.19198300	-4.22767000	2.29514400
C	1.60600800	-2.55294400	1.02417400
H	1.70416300	-3.14287300	0.12408900
N	1.78259900	-1.22067600	0.89788500
C	-0.70324600	1.27078400	-0.76875600
N	-0.44649800	2.54652700	-1.08870900
H	-1.22019300	3.15719900	-0.86175000
C	0.54100100	3.09497900	-2.00897500
H	0.60612800	2.48637600	-2.91255100
H	1.53055000	3.17959800	-1.56412200
H	0.21644700	4.09561300	-2.29492300
N	0.06277100	0.19252200	-0.87744600
H	-1.74260500	1.16775800	-0.39958500
C	-0.75686700	-1.00022700	-0.74453500
C	-1.11547500	-1.71799600	-1.88302500
H	-0.70821100	-1.42074000	-2.84214200
C	-2.03316100	-2.77540800	-1.80844700
H	-2.30669200	-3.30758200	-2.71135300
C	-2.60951900	-3.12708800	-0.59069300
H	-3.32145800	-3.94162400	-0.53529500
C	-2.24299400	-2.43540100	0.58428900
H	-2.56586800	-2.80279800	1.55346700
C	-1.30335500	-1.37431400	0.50521800
H	-0.94668200	-0.89026400	1.40715600
C	3.91475800	-0.63330600	-0.64916900
O	5.04286400	-0.88537000	-0.50175700
C	2.56164100	0.72695400	-2.30944400
O	2.86753900	1.29962700	-3.28106500
C	1.97087600	-1.66074400	-1.90900500
O	1.90984800	-2.59852300	-2.60179800
O	-3.49908300	1.23062300	0.08235900
Cu	-3.28200500	-0.56433300	0.38558800
Cu	-5.23759400	1.68163900	0.10588000

Intermediate I11 (B3P86-D3/6-31+G(d),LanL2DZ(Ag) model)

Mn	1.73670200	1.06806700	0.21055000
N	-0.15726600	1.73967200	-0.25382200
C	-0.49723700	2.40057900	-1.36782600
H	0.22088100	2.37865500	-2.17898800
C	-1.68096600	3.11335400	-1.48994000
H	-1.88790900	3.65730700	-2.40544200
C	-2.54319400	3.16435700	-0.39528000
H	-3.44369100	3.77107600	-0.42361800
C	-2.21627200	2.44533300	0.74515200
H	-2.87053900	2.46384100	1.60831000
C	-1.03276000	1.71005800	0.77420100
C	-0.60620400	0.89594100	1.91545200
C	-1.43018700	0.59253100	2.99530800
H	-2.45193700	0.95077800	3.01927200

C	-0.93790500	-0.19024900	4.02609600
H	-1.56225200	-0.42995700	4.88099200
C	0.36527800	-0.66592300	3.93999800
H	0.79782100	-1.28417700	4.71895300
C	1.12436700	-0.33206900	2.83057700
H	2.14537800	-0.68070500	2.73074200
N	0.66070900	0.43757400	1.83762400
C	1.40155800	-0.77542400	-0.71019500
N	0.16462900	-1.17757700	-0.99301200
H	-0.67815900	-0.71985100	-0.59237200
C	-0.17391900	-2.38425100	-1.72380800
H	0.27633800	-2.39224500	-2.72400200
H	-1.25823900	-2.39829800	-1.83769400
H	0.12587000	-3.29917800	-1.19279900
N	2.35287200	-1.62754700	-1.14815800
H	2.04251900	-2.50060800	-1.56441500
C	3.77010600	-1.58252700	-0.96916900
C	4.34778400	-2.39556900	0.00426600
H	3.70912800	-2.99173200	0.65029000
C	5.72938800	-2.43069400	0.14727700
H	6.17743300	-3.05997300	0.91028300
C	6.53539700	-1.65880800	-0.68486400
H	7.61473600	-1.68308600	-0.57083100
C	5.95694800	-0.86530900	-1.67049400
H	6.58330500	-0.27713400	-2.33427000
C	4.57470600	-0.83615400	-1.82522000
H	4.12114600	-0.24718200	-2.61451100
C	2.00545900	2.70542600	0.95396900
O	2.20033100	3.74556600	1.40042200
C	2.47679900	1.63038400	-1.31754500
O	2.91288700	2.01851600	-2.31220600
C	3.33846600	0.62590900	0.91186500
O	4.34024400	0.47180100	1.45670800
Ag	-3.54443400	0.44727800	-1.33045700
O	-2.33973200	-0.61808000	-0.04297700
Ag	-3.35304500	-2.35170100	0.30925300

Intermediate I12 (B3P86-D3/6-31+G(d),LanL2DZ(Ag) model)

Mn	1.07671300	-0.18473900	1.11830100
N	2.83211400	0.78577900	0.63767100
C	4.06367500	0.29897300	0.83561800
H	4.12891700	-0.72646200	1.17616500
C	5.21308700	1.04065400	0.61997000
H	6.18129000	0.59019100	0.80574200
C	5.08793200	2.34596700	0.16444900
H	5.96401300	2.95925900	-0.02057500
C	3.81756000	2.86106900	-0.03731000
H	3.69611000	3.88380500	-0.37301900
C	2.70412000	2.06394200	0.22131900
C	1.32241000	2.54626500	0.12757300
C	0.98773200	3.83421400	-0.28015600
H	1.75717900	4.53288100	-0.58560000
C	-0.34298800	4.22415100	-0.28238100
H	-0.61879500	5.22891400	-0.58661100
C	-1.30338500	3.30497800	0.11163000
H	-2.35784400	3.55686300	0.13119800
C	-0.90627800	2.03012300	0.49293900
H	-1.65310300	1.27792600	0.74304800
N	0.37925900	1.65429500	0.50717100
C	0.74740100	-0.76409200	-0.87257800
N	-0.49067300	-0.71506400	-1.35381900

H	-1.30678800	-0.42718300	-0.77143400
C	-0.88387300	-1.01511200	-2.71809300
H	-0.47162100	-0.29750300	-3.44082100
H	-1.97112100	-0.94822800	-2.76099500
H	-0.58707300	-2.02866600	-3.01461800
N	1.66734200	-1.08273700	-1.81114000
H	1.34521100	-1.14475500	-2.77299300
C	3.04737600	-1.40168800	-1.68507300
C	3.46059500	-2.54212200	-1.00185100
H	2.72521400	-3.17833900	-0.52317200
C	4.81073300	-2.87818000	-0.96943600
H	5.12728900	-3.76814500	-0.43392500
C	5.74338700	-2.10379400	-1.65356200
H	6.79264300	-2.38253400	-1.64610900
C	5.32127000	-0.98762800	-2.37191500
H	6.03998800	-0.39197200	-2.92663700
C	3.97729100	-0.63430400	-2.38569400
H	3.64093400	0.23827900	-2.93899700
C	1.25643200	0.46041800	2.80978200
O	1.35135500	0.85819400	3.88218600
C	1.85190000	-1.72320900	1.60923200
O	2.30921400	-2.70839400	1.99643100
C	-0.44676900	-0.96750000	1.62089900
O	-1.34971400	-1.55658500	2.04241900
Ag	-4.51439200	0.80619300	-1.14085400
O	-2.87832500	-0.02521200	-0.25527300
Ag	-3.75591600	-1.64352500	0.68690400

Transition state TS(1-2) (B3LYP/LanL2DZ model)

Mn	-2.27176900	0.75811700	-0.70322700
N	-3.51601400	-0.90173400	-0.59987400
C	-4.02100400	-1.57402100	-1.67784500
H	-3.62726700	-1.28456100	-2.64646700
C	-4.99416900	-2.57159800	-1.54917300
H	-5.36731900	-3.07533500	-2.43708300
C	-5.47660300	-2.89431700	-0.26638600
H	-6.24084700	-3.65709100	-0.13682800
C	-4.96314100	-2.21186500	0.84552900
H	-5.33274400	-2.43741800	1.84187600
C	-3.97932600	-1.22079900	0.66334600
C	-3.37618700	-0.43886100	1.74858300
C	-3.59735700	-0.68740100	3.11717000
H	-4.24592700	-1.50204600	3.42738200
C	-2.96829700	0.11520500	4.07837400
H	-3.13163000	-0.06571000	5.13817900
C	-2.12474300	1.15506200	3.64698300
H	-1.61897300	1.80340200	4.35794500
C	-1.93769900	1.35609400	2.27455200
H	-1.29502500	2.14608500	1.90266400
N	-2.54860700	0.58313900	1.33142400
C	-0.56005700	-0.44843700	-0.35460900
N	-0.78502400	-1.78024700	-0.17978800
H	-1.73439300	-2.09130300	-0.31128700
C	0.22486500	-2.82662200	0.08311400
H	0.92458300	-2.90600800	-0.76438600
H	-0.29902600	-3.78095100	0.21288900
H	0.81274200	-2.59538500	0.98139400
N	0.70899900	-0.04210500	-0.20569300
H	1.61296400	-0.69807400	0.18107400
C	1.17865100	1.28017700	-0.47007300
C	1.60624500	2.08568800	0.61767500

H	1.38047800	1.75732300	1.63321600
C	2.21538800	3.33672400	0.37380600
H	2.50615400	3.97006300	1.21111700
C	2.40429600	3.78080700	-0.95138700
H	2.86571400	4.74784100	-1.14115900
C	1.97195000	2.98080000	-2.02519800
H	2.10709600	3.32669900	-3.04863700
C	1.37261400	1.72906000	-1.78860900
H	1.05661600	1.09670300	-2.61564500
C	-3.80598500	1.68066100	-1.00234900
O	-4.81255000	2.25649100	-1.22589900
C	-1.86600700	0.60727200	-2.43226500
O	-1.56207600	0.47534200	-3.57177200
C	-1.47225600	2.35904400	-0.60642900
O	-1.11744900	3.48849100	-0.52926900
Ag	3.84580500	-2.42859000	-0.56251400
O	2.76096800	-1.20322200	0.71663900
Ag	3.86201100	0.63256000	0.95199300

Transition state TS(2-3) (B3LYP/LanL2DZ model)

Mn	-2.59734000	0.51703700	-0.49216200
N	-3.28428700	-1.36931700	-0.84865900
C	-3.55123100	-1.89213400	-2.07572700
H	-3.23274400	-1.30833600	-2.92972300
C	-4.21136900	-3.11137900	-2.24167300
H	-4.40704500	-3.48224100	-3.24072200
C	-4.61564800	-3.82669300	-1.10324900
H	-5.14049500	-4.77059200	-1.20049000
C	-4.33926400	-3.29773400	0.16151500
H	-4.65361400	-3.82785700	1.05232000
C	-3.66876900	-2.06925400	0.26627100
C	-3.34053800	-1.40785700	1.53295800
C	-3.53991300	-1.98667300	2.79547900
H	-3.95558400	-2.98325900	2.88288600
C	-3.19582400	-1.26724600	3.94337600
H	-3.34777400	-1.69590100	4.92777900
C	-2.65264000	0.01757200	3.79531500
H	-2.37346100	0.61436600	4.65559900
C	-2.47602900	0.53780500	2.51114600
H	-2.06047200	1.52558100	2.36449400
N	-2.81450600	-0.15221700	1.39384000
C	-0.59591600	-0.12529200	-0.35950600
N	-0.43841700	-1.48200800	-0.49431900
H	-1.27904800	-1.99011800	-0.71241000
C	0.78718500	-2.26735700	-0.40546600
H	1.49974200	-2.01275300	-1.20361700
H	0.53112500	-3.32577200	-0.51066300
H	1.28257200	-2.13324200	0.56530800
N	0.50757100	0.58973900	-0.13921900
H	5.10107200	0.91005200	-0.39049700
C	0.50240000	2.01985500	-0.01581600
C	0.66311000	2.60935300	1.25375600
H	0.72687100	1.96648100	2.12813400
C	0.75300800	4.00204700	1.37816600
H	0.86733100	4.45005000	2.36102800
C	0.69557100	4.81962000	0.23736900
H	0.76135800	5.89854300	0.33515500
C	0.55782000	4.23272200	-1.03031000
H	0.51846400	4.85762600	-1.91747400
C	0.46649000	2.83999800	-1.16081600
H	0.37730700	2.38206800	-2.14129300

C	-4.32493300	0.99889900	-0.68349200
O	-5.44890600	1.30825600	-0.84289400
C	-2.17373500	0.88601800	-2.18022200
O	-1.84553300	1.10094800	-3.29375700
C	-2.33537800	2.22804400	0.00318800
O	-2.39144100	3.35638700	0.33668400
Ag	6.12434700	-1.45264200	0.11063100
O	4.70497700	0.04135700	-0.18806900
Ag	2.58514600	0.15048300	-0.15174900

Transition state TS(3-4) (B3LYP/LanL2DZ model)

Mn	1.97269300	-0.47686100	1.17893400
N	3.19338500	-1.09448700	-0.36420500
C	3.52926900	-2.37891300	-0.64813500
H	3.15064500	-3.14068200	0.02011200
C	4.34554800	-2.72664700	-1.73272000
H	4.58501400	-3.76912800	-1.91281600
C	4.83817800	-1.70533200	-2.56381000
H	5.46244700	-1.93855200	-3.42088400
C	4.53123500	-0.37285000	-2.25027200
H	4.92729000	0.43067700	-2.85992600
C	3.72256100	-0.08971800	-1.13398800
C	3.43832200	1.27403700	-0.65075700
C	3.97358100	2.43803200	-1.23252300
H	4.59250000	2.37594000	-2.11992100
C	3.71526200	3.68529500	-0.64558500
H	4.12555800	4.59197100	-1.07953700
C	2.92650700	3.73767500	0.51692100
H	2.70615200	4.67758800	1.01069400
C	2.41198900	2.54432900	1.04006200
H	1.79332400	2.55539500	1.92735500
N	2.65412300	1.33409500	0.47166200
C	0.14104800	-0.89734700	-1.58370700
N	0.31517000	-2.24183200	-1.74455400
H	0.20675900	-2.51268400	-2.71454400
C	0.21045700	-3.37243200	-0.79019900
H	1.17728000	-3.71392500	-0.41500800
H	-0.26788700	-4.21559800	-1.30104600
H	-0.41162000	-3.09468900	0.06790400
N	0.23033100	-0.27993700	-0.35500900
H	-4.52384400	-2.13136100	0.14138200
C	-0.14365700	1.14736700	-0.45666000
C	-0.91403500	1.74976100	0.56299700
H	-1.23305400	1.16457300	1.42114500
C	-1.29434900	3.10337800	0.47621800
H	-1.88744100	3.54217800	1.27534900
C	-0.91676500	3.87927900	-0.63523400
H	-1.21444700	4.92202300	-0.70742900
C	-0.15944700	3.27825500	-1.66131400
H	0.12437700	3.85779300	-2.53693400
C	0.22542600	1.93016200	-1.57436900
H	0.77275800	1.45605900	-2.38186300
C	3.39908700	-0.52954400	2.28981600
O	4.34144100	-0.55761800	2.99425500
C	1.45759800	-2.11383100	1.76111900
O	1.14515700	-3.14869600	2.23537500
C	0.93848000	0.20954700	2.50220800
O	0.30473200	0.61209200	3.41207400
Ag	-5.79561600	0.20567500	-0.06482800
O	-4.24305300	-1.21303200	-0.04429100
Ag	-2.13626000	-0.87473800	-0.32373300

Transition state TS(4-5) (B3LYP/LanL2DZ model)

Mn	2.64379400	-0.60678600	-0.38891100
N	2.80301300	1.45576400	-0.39393300
C	3.32663400	2.20554200	-1.38325900
H	3.71675800	1.65733100	-2.23790000
C	3.36187100	3.59989900	-1.33996000
H	3.80038200	4.15445400	-2.16884600
C	2.82129000	4.24940000	-0.22311900
H	2.81113300	5.33768600	-0.15973100
C	2.32755100	3.47739900	0.83127100
H	1.94842800	3.96072200	1.72939500
C	2.35814600	2.07803300	0.73685600
C	2.05606600	1.17392500	1.85540200
C	1.64484900	1.60501300	3.12712100
H	1.42232100	2.65484600	3.30710400
C	1.55079700	0.68022600	4.17025500
H	1.24307700	1.00384800	5.16490100
C	1.88087300	-0.65757500	3.92012500
H	1.84960100	-1.41265900	4.70457100
C	2.24272400	-1.02530800	2.62290500
H	2.47551200	-2.05891000	2.37885800
N	2.31020100	-0.14341300	1.60545900
C	-0.40791500	0.64013700	-1.00741200
N	-0.13449000	1.63527300	-1.87954600
H	-0.95660900	2.20391100	-2.05779000
C	0.79827200	1.62201000	-3.02033200
H	1.57617200	0.88464200	-2.84346900
H	1.25795500	2.61259700	-3.14367400
H	0.26148200	1.35436300	-3.94464700
N	0.42887800	-0.28032800	-0.57536800
H	-2.34051800	1.11707000	-0.50549900
C	-0.22204400	-1.36026100	0.09465000
C	-0.58695200	-2.51631900	-0.64494700
H	-0.25654900	-2.60162900	-1.68125100
C	-1.24635100	-3.59186600	-0.00664300
H	-1.48693200	-4.48848700	-0.57928600
C	-1.57062800	-3.50714200	1.35557000
H	-2.07939700	-4.33474300	1.85015700
C	-1.23495600	-2.34962000	2.07479100
H	-1.50665000	-2.26889600	3.12886300
C	-0.57388100	-1.28228000	1.45209000
H	-0.35423200	-0.36715400	1.99818500
C	4.41084700	-0.75297800	-0.14863700
O	5.56054200	-0.84080100	-0.00430500
C	2.82992900	-0.92401400	-2.13780400
O	2.98305400	-1.18542700	-3.26346300
C	2.48465700	-2.37824100	-0.16654600
O	2.45677500	-3.53163100	-0.00879100
Ag	-4.71976300	1.54411000	0.58672500
O	-3.27656400	0.96483900	-0.82836700
Ag	-2.69792000	-1.16287900	-1.15371000

Transition state TS(5-Products) (B3LYP/LanL2DZ model)

Mn	-2.68441400	0.69010800	-0.54148500
N	-2.87304900	-1.34838500	-0.73708300
C	-3.23462500	-2.00780700	-1.86780400
H	-3.43044000	-1.40194200	-2.74331700
C	-3.36404200	-3.40187900	-1.91954600
H	-3.66757100	-3.87931600	-2.84492100
C	-3.09803200	-4.15003200	-0.75975900

H	-3.17543100	-5.23279500	-0.76787500
C	-2.76397200	-3.47099400	0.42107700
H	-2.59807600	-4.02768900	1.33585300
C	-2.68240300	-2.06700800	0.41469600
C	-2.52342400	-1.24897500	1.63144500
C	-2.38332600	-1.78484100	2.92455700
H	-2.28121400	-2.85385200	3.06975900
C	-2.40109000	-0.92609000	4.03414700
H	-2.30387300	-1.32546100	5.03890700
C	-2.56753400	0.45282000	3.82212500
H	-2.61253200	1.15281000	4.64911100
C	-2.66784800	0.92793300	2.50710400
H	-2.77245900	1.98698600	2.31268600
N	-2.63264200	0.10276500	1.42886200
C	0.43789200	-0.43962900	-0.74008700
N	0.28893400	-1.45068300	-1.65821500
H	1.02774000	-2.14223500	-1.59561500
C	-0.33499900	-1.37359800	-2.99418400
H	-1.04297100	-0.55186600	-3.02889000
H	-0.85301500	-2.30654900	-3.23831100
H	0.43132200	-1.19155100	-3.76134200
N	-0.44872700	0.51788400	-0.49454700
H	1.81178600	-0.46125200	-0.23580100
C	0.11090100	1.65821900	0.22642700
C	0.49523600	2.80032600	-0.50503600
H	0.35936100	2.81040600	-1.58379200
C	1.03578700	3.91829600	0.15261800
H	1.32073700	4.79447600	-0.42440800
C	1.19722900	3.91557300	1.55337100
H	1.58337500	4.79299500	2.06496200
C	0.81430000	2.77204400	2.28464000
H	0.92930500	2.74796100	3.36580200
C	0.28955800	1.64331700	1.62392100
H	0.04551800	0.74461300	2.18100100
C	-4.49102600	0.78694800	-0.50514100
O	-5.66691100	0.81984600	-0.48257400
C	-2.62720500	1.10929800	-2.30142600
O	-2.58762400	1.42012600	-3.43856500
C	-2.61213100	2.47556900	-0.16522100
O	-2.67508500	3.62510400	0.07398500
Ag	4.10431300	-1.75622700	1.08761600
O	2.91860400	-0.44820500	-0.03023800
Ag	4.15759800	0.72364400	-1.25864200

Transition state TS(6-7) (B3LYP/LanL2DZ model)

Mn	-1.73035100	1.00364700	-0.24962900
N	-3.07089700	-0.38151300	-1.02892000
C	-3.40978500	-0.49704200	-2.34675600
H	-2.83917500	0.10648800	-3.04403300
C	-4.43880200	-1.33860900	-2.78574100
H	-4.67221000	-1.39200100	-3.84590300
C	-5.15613200	-2.09285300	-1.83889500
H	-5.96898400	-2.74530600	-2.14908000
C	-4.80958100	-1.98981800	-0.48438800
H	-5.35527200	-2.56065700	0.26149200
C	-3.76048600	-1.13454800	-0.09524700
C	-3.30030300	-0.96260400	1.28919000
C	-3.79326900	-1.70306100	2.38107600
H	-4.57054300	-2.44718800	2.23196800
C	-3.27382700	-1.47858400	3.66327600
H	-3.64839300	-2.04217400	4.51451500

C	-2.26317900	-0.51436400	3.82684100
H	-1.83091800	-0.30824100	4.80255200
C	-1.81160400	0.19529300	2.70760200
H	-1.04031700	0.95187000	2.79664500
N	-2.31353300	-0.01249900	1.45704900
C	-0.15576500	-0.39198300	-0.37805600
N	-0.47047000	-1.70503100	-0.52232900
H	-1.44761500	-1.93280200	-0.61588100
C	0.48959900	-2.83209300	-0.59653200
H	1.17253300	-2.69844600	-1.44781500
H	-0.08165200	-3.75874400	-0.72047600
H	1.10159500	-2.88730700	0.31482000
N	1.16250600	-0.14317900	-0.26049200
H	1.92491700	-0.91501600	-0.20778700
C	1.82326900	1.12708500	-0.17933200
C	2.51953400	1.41669200	1.03607500
H	2.22552300	0.89361000	1.94741700
C	3.43715400	2.50029800	1.07661700
H	3.92535000	2.76068800	2.01420700
C	3.62674600	3.30533000	-0.07592600
H	4.32437700	4.13969300	-0.04344400
C	2.88832700	3.04464100	-1.24036000
H	3.01869600	3.67335500	-2.11903000
C	2.00625700	1.94075200	-1.30238300
H	1.50548300	1.69141600	-2.23339200
C	-3.14269800	2.14222500	-0.13950200
O	-4.06218700	2.87955000	-0.08920400
C	-1.23561100	1.65695300	-1.83114400
O	-0.93026500	2.06674800	-2.90159300
C	-0.76298500	2.22532800	0.62960700
O	-0.23710300	3.08068500	1.26027400
O	3.39579000	-1.51098900	0.02713000
Cu	4.18413900	0.12716000	0.54166100
Cu	4.44245000	-2.87774800	-0.54173700

Transition state TS(7-8) (B3LYP/LanL2DZ model)

Mn	-1.86730200	0.71337700	-0.51784800
N	-2.81649900	-1.10627100	-0.82197700
C	-3.21733800	-1.59099900	-2.03713600
H	-2.86879500	-1.04183700	-2.90554200
C	-4.03515500	-2.71932300	-2.16030200
H	-4.33060100	-3.06289600	-3.14829900
C	-4.46800000	-3.38093700	-0.99459800
H	-5.11523000	-4.25249300	-1.05951300
C	-4.06023500	-2.89416200	0.25529700
H	-4.39639900	-3.38122100	1.16646100
C	-3.23004800	-1.75858500	0.32581100
C	-2.75151800	-1.15264400	1.57259700
C	-2.93333000	-1.72175200	2.84829800
H	-3.45519800	-2.66888800	2.95462500
C	-2.43208600	-1.06304700	3.97890700
H	-2.57057400	-1.48867000	4.97009300
C	-1.74847300	0.15503800	3.80705100
H	-1.34291800	0.69971300	4.65589400
C	-1.59288600	0.67438000	2.51693100
H	-1.06584800	1.60538700	2.34124500
N	-2.08792000	0.04655800	1.41126800
C	0.04856500	-0.22531600	-0.41034500
N	0.02017700	-1.58643100	-0.58191300
H	-0.88090600	-1.97289000	-0.81271800
C	1.13597500	-2.54130700	-0.52019600

H	1.87486800	-2.35520200	-1.31929300
H	0.72480200	-3.55089400	-0.64933300
H	1.64771500	-2.48718200	0.45526400
N	1.23283000	0.34141600	-0.16992800
H	5.56964700	0.10364100	-0.29571000
C	1.39861000	1.76664700	-0.01166800
C	1.58957600	2.30511500	1.27883900
H	1.55015000	1.63670000	2.13881400
C	1.83395500	3.67954500	1.43427500
H	1.96810800	4.09765100	2.43108600
C	1.90445800	4.51802900	0.30405500
H	2.08891500	5.58364700	0.42654400
C	1.73730300	3.97391800	-0.98403500
H	1.79355800	4.61859600	-1.85980200
C	1.49128000	2.59993000	-1.14655500
H	1.36397100	2.16480300	-2.13613500
C	-3.53714100	1.39696700	-0.68200600
O	-4.62963300	1.82386200	-0.82853300
C	-1.43306800	1.01740100	-2.21975500
O	-1.10736700	1.18740400	-3.34817200
C	-1.39523200	2.38234900	-0.04107300
O	-1.31421000	3.51794800	0.28787900
O	4.93050600	-0.63111800	-0.20188100
Cu	3.05777200	-0.24515600	-0.15879000
Cu	5.59124700	-2.36386200	0.04739900

Transition state TS(8-9) (B3LYP/LanL2DZ model)

Mn	1.35856100	0.20914200	1.19932800
N	2.85484400	-0.75634100	0.14449800
C	3.48306600	-1.89686600	0.53959600
H	3.07946700	-2.40150800	1.40867500
C	4.60558000	-2.40917200	-0.12409100
H	5.07495800	-3.31789900	0.23690700
C	5.10230800	-1.72060700	-1.24629100
H	5.97041500	-2.08938600	-1.78387800
C	4.47097900	-0.53298400	-1.64603800
H	4.85834700	0.02773500	-2.48844200
C	3.35549600	-0.06305300	-0.92747200
C	2.68483900	1.22069000	-1.20088400
C	2.98720400	2.04249700	-2.30061900
H	3.71881400	1.73013100	-3.03648300
C	2.33368500	3.27615100	-2.44426000
H	2.55914600	3.92257400	-3.28678500
C	1.39199000	3.66011600	-1.47442900
H	0.87232300	4.61024800	-1.53406400
C	1.12331900	2.79026400	-0.40831200
H	0.40190100	3.06220300	0.35052800
N	1.74557100	1.58972700	-0.26974100
C	-0.14915400	-0.70771000	-1.55788400
N	0.61966100	-1.79983400	-1.82465200
H	0.75047900	-1.95377800	-2.81872100
C	0.90275600	-2.98833600	-0.97885300
H	0.87669000	-2.71833700	0.07369800
H	1.88367100	-3.40448300	-1.22546200
H	0.14436000	-3.76611300	-1.15624800
N	-0.45189400	-0.46947300	-0.21043900
H	-4.58868200	-2.61205400	-1.09265100
C	-1.52627800	0.51204700	-0.04585200
C	-2.29137400	0.46861100	1.14859800
H	-1.98212200	-0.18207700	1.96013500
C	-3.50057000	1.19412800	1.26793100

H	-4.04125900	1.18295800	2.21050500
C	-3.96973400	1.98982600	0.17669600
H	-4.85613600	2.60836600	0.28719300
C	-3.13278400	2.12308200	-0.97124700
H	-3.44200300	2.77772900	-1.78149800
C	-1.93013000	1.41237800	-1.07598100
H	-1.33625300	1.45050100	-1.98146400
C	2.71083700	0.74918800	2.26998000
O	3.60737200	1.08532800	2.95296100
C	0.89819900	-1.19823600	2.25120500
O	0.55404000	-2.11981900	2.89941300
C	0.29825600	1.32037000	2.17449100
O	-0.25999700	2.08515300	2.87641800
O	-4.05118000	-1.89567300	-0.71716900
Cu	-2.15439700	-1.74222900	-0.64151100
Cu	-4.57588600	-0.14736100	-0.18102300

Transition state TS(9-10) (B3LYP/LanL2DZ model)

Mn	-2.20911800	-0.06154800	-0.90046300
N	-2.34506500	-1.26609400	0.77060600
C	-2.70223700	-2.57637000	0.78394500
H	-2.99026100	-3.01307600	-0.16331900
C	-2.73574000	-3.34070100	1.95875900
H	-3.03099300	-4.38337900	1.91347800
C	-2.38757700	-2.72767800	3.17504500
H	-2.39034300	-3.29167300	4.10255700
C	-2.06182100	-1.36280600	3.17463700
H	-1.82364900	-0.86569400	4.10761500
C	-2.06437700	-0.64737200	1.96248900
C	-1.85941500	0.81224800	1.87145900
C	-1.67415200	1.64822200	2.98922300
H	-1.61866000	1.23046900	3.98757000
C	-1.58552100	3.03707300	2.80691400
H	-1.45355700	3.69482400	3.66054000
C	-1.68659900	3.55999900	1.50563300
H	-1.63766000	4.62699400	1.31791500
C	-1.85439700	2.67389300	0.43254200
H	-1.92627500	3.04609100	-0.58138700
N	-1.93489200	1.32790500	0.60363500
C	0.84601000	-1.33970400	-0.68983200
N	0.71507700	-2.67301400	-0.88043400
H	1.56288700	-3.16931100	-0.62203900
C	-0.25896600	-3.44858100	-1.65856400
H	-0.43801400	-2.98745100	-2.63386100
H	-1.21108600	-3.56948700	-1.13888500
H	0.15794700	-4.44573900	-1.82739300
N	-0.01482100	-0.33101200	-0.86089900
H	1.87756400	-1.12206200	-0.35826200
C	0.71359200	0.93759000	-0.78962300
C	1.01061300	1.64915500	-1.96339900
H	0.64384700	1.28179700	-2.91678700
C	1.82955100	2.80161000	-1.92725800
H	2.05549200	3.32240300	-2.85317700
C	2.37298800	3.25619800	-0.71506600
H	3.00796200	4.13697900	-0.69042500
C	2.07391000	2.56425900	0.49094400
H	2.37417100	2.98357500	1.44930900
C	1.23772700	1.40726000	0.45075900
H	0.93277800	0.92264600	1.37410400
C	-3.99632300	0.24687500	-0.77967200
O	-5.15040200	0.44240200	-0.67379100

C	-2.53610100	-1.34074300	-2.14683800
O	-2.82855400	-2.11337900	-2.98726800
C	-2.07400000	1.13193800	-2.26806100
O	-2.03892900	1.89524900	-3.16352500
O	3.67299000	-1.05438700	0.18200800
Cu	3.32978000	0.73610400	0.41162800
Cu	5.40504300	-1.53875800	0.21285600

Transition state TS(10-Products) (B3LYP/LanL2DZ model)

Mn	-2.25061400	-0.41022800	-0.88264000
N	-1.94520800	-1.59705000	0.79446300
C	-1.87130400	-2.95307300	0.81012100
H	-2.01649400	-3.45966800	-0.13503200
C	-1.64747000	-3.68764400	1.98263200
H	-1.60003300	-4.77018800	1.93796800
C	-1.49484000	-2.99368900	3.19590000
H	-1.31144700	-3.52830700	4.12266400
C	-1.60833500	-1.59504400	3.19341100
H	-1.52386400	-1.04629000	4.12376200
C	-1.84881000	-0.91784000	1.98298100
C	-2.08830700	0.53772900	1.89319400
C	-2.12013400	1.39375300	3.00996100
H	-1.91999500	1.01167400	4.00390900
C	-2.43320400	2.75038500	2.83321700
H	-2.46880500	3.42071600	3.68646000
C	-2.71088200	3.22034900	1.53763800
H	-2.96739000	4.25749300	1.35255600
C	-2.65086100	2.31967100	0.46560100
H	-2.85020700	2.65420200	-0.54415200
N	-2.34273600	1.00690900	0.63048200
C	1.02120600	-0.73545000	-0.79445000
N	1.32220000	-2.03028700	-1.02759800
H	2.29521100	-2.21291000	-0.77957100
C	0.62158700	-3.05711500	-1.80463900
H	-0.21835800	-3.49554700	-1.26042400
H	1.33108800	-3.85959300	-2.02613100
H	0.25821200	-2.65342600	-2.75429200
N	-0.08877000	-0.00892400	-0.91453600
H	1.92231400	-0.22868500	-0.45231600
C	0.26273500	1.40864800	-0.74665300
C	0.17932900	2.29163900	-1.84028400
H	-0.17290400	1.92984700	-2.80070600
C	0.59755300	3.63059500	-1.70692500
H	0.53347000	4.29676100	-2.56321600
C	1.11791200	4.09975800	-0.48541400
H	1.44389300	5.13160100	-0.38788500
C	1.21590700	3.21519500	0.60892100
H	1.59938900	3.56875100	1.56397700
C	0.78959700	1.87839200	0.48017600
H	0.85502200	1.20145000	1.32935800
C	-4.03770500	-0.65727900	-0.67794800
O	-5.19258000	-0.82041400	-0.52754500
C	-2.23287200	-1.73786000	-2.11630000
O	-2.33101400	-2.57089600	-2.94542300
C	-2.55268700	0.75453300	-2.24443500
O	-2.80860400	1.47696700	-3.13874200
O	3.87428300	-0.77395900	0.08101300
Cu	3.95839300	1.01436800	0.32232300
Cu	5.59576800	-1.25727300	0.09335800

Transition state TS(11-12) (B3LYP/LanL2DZ model)

Mn	-1.82922400	1.04328400	0.32472900
N	-0.72004000	0.32749000	1.90532100
C	-1.17035600	-0.51817900	2.86976800
H	-2.18527100	-0.88039900	2.76789200
C	-0.38034800	-0.90402200	3.96107100
H	-0.79077600	-1.57267300	4.71014900
C	0.92968200	-0.40101400	4.06414800
H	1.56376900	-0.67322700	4.90255800
C	1.40401600	0.45958600	3.06397500
H	2.41519900	0.84548600	3.11191500
C	0.56071100	0.81105200	1.99376800
C	0.96513400	1.69869200	0.88809200
C	2.16984700	2.42455700	0.86217300
H	2.85961500	2.37006000	1.69566700
C	2.46829400	3.22908000	-0.24702500
H	3.37885900	3.82100000	-0.26785300
C	1.55719600	3.27171200	-1.32132700
H	1.74267000	3.88115400	-2.19971500
C	0.36220800	2.54469600	-1.22286900
H	-0.37291800	2.58308200	-2.01675200
N	0.05333200	1.79284400	-0.13263400
C	-1.47541500	-0.74615200	-0.71822600
N	-0.21010800	-1.14575100	-0.98605300
H	0.61778400	-0.65623800	-0.61483300
C	0.16915100	-2.34094800	-1.76779600
H	-0.16877500	-3.27403400	-1.29129500
H	1.25876900	-2.35111300	-1.81517700
H	-0.22721700	-2.30120600	-2.79273000
N	-2.42409200	-1.59323600	-1.23609200
H	-2.09912600	-2.41653400	-1.73999600
C	-3.86777100	-1.55967100	-1.10706900
C	-4.65433200	-0.77951100	-1.97594700
H	-4.17894200	-0.14860000	-2.72000100
C	-6.05772200	-0.83493300	-1.88523200
H	-6.66291400	-0.22554600	-2.55091400
C	-6.67541900	-1.68378600	-0.94723900
H	-7.75924700	-1.72616800	-0.88197100
C	-5.88540200	-2.48528800	-0.09998200
H	-6.35813600	-3.14745300	0.62034500
C	-4.48347500	-2.42616400	-0.18150000
H	-3.86689600	-3.04080500	0.47027900
C	-2.10452400	2.63531500	1.19391600
O	-2.29244600	3.66294100	1.73556900
C	-3.41961500	0.50191100	1.01839100
O	-4.43000800	0.26282600	1.57500600
C	-2.59519000	1.67878500	-1.17779600
O	-3.04286100	2.10775500	-2.18279900
Ag	3.59321300	-2.17737600	0.48140200
O	2.44333200	-0.66702100	-0.34631500
Ag	3.66947500	0.40663900	-1.63440300

Table S3. Selected bond lengths (Å) of theoretically optimized structures (B3P86-D3/6-31+G(d),LanL2DZ(Ag) level of theory) in mechanism *M1*. See Figure 1 for the atomic numbering scheme.

Compound	Mn1-C1	C1-N1	C1-N2	N1-H1	C1-H1	H1-O1	N1-Ag1	C1-Ag1	O1-Ag1	O1-Ag2	Mn1-N1
**1+Ag$_2$O**	2.083	1.341	1.341	1.015	-----	-----	-----	-----	2.027	2.027	-----
**I1**	2.102	1.335	1.338	1.080	-----	1.582	------	------	2.083	2.025	-----
**TS(1-2)**	2.138	1.346	1.369	1.259	-----	1.260	------	-----	2.133	2.060	-----
**I2**	2.116	1.307	1.359	2.192	-----	0.983	------	-----	2.184	2.077	-----
**TS(2-3)**	2.125	1.314	1.357	-----	-----	0.968	2.137	-----	2.129	2.092	-----
**I3**	2.129	1.316	1.356	-----	-----	0.967	2.134	-----	2.120	2.090	-----
**TS(3-4)**	3.341	1.378	1.365	-----	-----	0.978	2.440	2.603	2.152	2.103	2.330
**I4**	-----	1.323	1.360	-----	-----	0.968	-----	2.120	2.135	2.142	2.215
**TS(4-5)**	-----	1.346	1.365	-----	1.856	1.024	-----	-----	2.200	2.077	2.278
**I5**	-----	1.314	1.333	-----	1.106	1.806	-----	-----	2.067	2.018	2.164
**TS(5-Products)**	-----	1.310	1.343	-----	1.095	1.807	-----	-----	2.094	2.068	2.629
**2+Ag$_2$O**	-----	1.304	1.335	-----	1.087	-----	-----	-----	2.027	2.027	2.111
**2′+Ag$_2$O**	-----	1.303	1.336	-----	1.092	-----	-----	-----	2.027	2.027	2.127
**2″+Ag$_2$O**	-----	1.305	1.341	-----	1.093	-----	-----	-----	2.027	2.027	2.195

Table S4. Selected bond angles (°) of theoretically optimized structures (B3P86-D3/6-31+G(d),LanL2DZ(Ag) level of theory) in mechanism *M1*. See Figure 1 for the atomic numbering scheme.

Compound	Mn1-C1-N1	Mn1-C1-N2	Mn1-N1-C1	Mn1-N1-C2	N1-C1-N2	N1-H1-O1	C1-H1-O1	N1-Ag1-O1	C1-Ag1-O1	Ag1-O1-Ag2
**1+Ag$_2$O**	128.24	118.35	-----	-----	113.39	-----	-----	-----	-----	94.36
**I1**	128.78	116.95	------	------	114.05	163.57	-----	-----	------	105.20
**TS(1-2)**	130.03	114.56	------	------	115.37	164.97	-----	-----	-----	113.43
**I2**	130.32	115.79	------	------	113.75	152.26	------	------	-----	143.09
**TS(2-3)**	129.44	114.04	------	------	116.51	-----	------	171.62	-----	141.32
**I3**	129.41	113.94	------	------	116.62	-----	------	173.27	-----	127.99
**TS(3-4)**	33.99	98.71	126.70	108.42	122.55	-----	------	171.64	156.65	127.98
**I4**	------	------	137.71	109.39	121.88	-----	------	------	167.30	88.10
**TS(4-5)**	-----	------	140.76	107.63	125.83	-----	150.32	-----	-----	141.43
**I5**	-----	-----	136.95	113.21	131.33	-----	157.73	-----	-----	111.98
**TS(5-Products)**	-----	------	143.10	104.24	127.48	-----	162.45	-----	-----	122.37
**2+Ag$_2$O**	-----	-----	123.29	120.98	127.13	-----	-----	-----	-----	94.36
**2′+Ag$_2$O**	-----	-----	129.66	118.25	127.53	-----	-----	-----	-----	94.36
**2″+Ag$_2$O**	-----	-----	135.79	112.70	130.71	-----	-----	-----	-----	94.36

Table S5. Selected bond lengths (Å) of theoretically optimized structures (B3P86-D3/6-31+G(d) level of theory) in mechanism *M2*. Labels as in Table S3.

Compound	Mn1-C1	C1-N1	C1-N2	N1-H1	C1-H1	H1-O1	N1-Cu1	C1-Cu1	O1-Cu1	O1-Cu2	Mn1-N1
**1+Cu_2O**	2.083	1.341	1.341	1.015	-----	-----	-----	-----	1.778	1.778	-----
**I6**	2.087	1.334	1.337	1.070	-----	1.678	------	------	1.848	1.776	-----
**TS(6-7)**	2.084	1.342	1.349	1.080	-----	1.602	------	-----	1.865	1.796	-----
**I7**	2.100	1.317	1.367	2.067	-----	0.996	-----	-----	1.916	1.833	-----
**TS(7-8)**	2.143	1.342	1.379	------	-----	0.976	1.912	-----	1.898	1.854	-----
**I8**	2.101	1.332	1.360	------	-----	0.966	1.944	-----	1.889	1.888	-----
**TS(8-9)**	3.274	1.401	1.362	------	-----	0.971	2.169	2.435	1.904	1.902	2.393
**I9**	------	1.348	1.360	-----	-----	0.966	-----	1.932	1.884	1.873	2.182
**TS(9-10)**	-----	1.356	1.373	-----	2.297	0.969	-----	-----	1.963	1.898	2.436
**I10**	-----	1.327	1.340	-----	1.108	1.822	-----	-----	1.833	1.796	2.160
**TS(10-Products)**	-----	1.332	1.350	-----	1.089	2.096	-----	-----	1.806	1.788	2.199
**2+Cu_2O**	-----	1.304	1.335	-----	1.087	-----	-----	-----	1.778	1.778	2.111
**2′+Cu_2O**	-----	1.303	1.336	-----	1.092	-----	-----	-----	1.778	1.778	2.127
**2′′+Cu_2O**	-----	1.305	1.341	-----	1.093	-----	-----	-----	1.778	1.778	2.195

Table S6. Selected bond angles (°) of theoretically optimized structures (B3P86-D3/6-31+G(d) level of theory) in mechanism *M2*. Labels as in Table S4.

Compound	Mn1-C1-N1	Mn1-C1-N2	Mn1-N1-C1	Mn1-N1-C2	N1-C1-N2	N1-H1-O1	C1-H1-O1	N1-Cu1-O1	C1-Cu1-O1	Cu1-O1-Cu2
**1+Cu_2O**	128.24	118.35	-----	-----	113.39	-----	-----	-----	-----	93.67
**I6**	128.33	118.15	-----	-----	113.45	156.65	-----	------	------	100.91
**TS(6-7)**	128.57	117.65	-----	-----	113.72	156.40	-----	-----	-----	110.36
**I7**	129.88	116.08	-----	------	113.99	148.28	------	-----	-----	136.95
**TS(7-8)**	130.43	113.39	-----	------	116.18	-----	------	171.83	-----	128.33
**I8**	129.80	115.72	-----	------	114.39	-----	------	140.54	-----	92.86
**TS(8-9)**	40.72	97.44	116.82	107.38	116.52	-----	------	146.82	147.07	100.92
**I9**	------	-----	139.79	109.09	123.33	-----	------	-----	164.70	96.13
**TS(9-10)**	-----	-----	131.28	107.31	117.29	-----	158.48	-----	-----	135.70
**I10**	-----	-----	136.34	113.04	130.04	-----	171.56	-----	-----	110.99
**TS(10-Products)**	-----	-----	135.92	114.17	133.91	-----	136.85	-----	-----	102.82
**2+Cu_2O**	-----	-----	123.29	120.98	127.13	-----	-----	-----	-----	93.67
**2′+Cu_2O**	-----	-----	129.66	118.25	127.53	-----	-----	-----	-----	93.67
**2′′+Cu_2O**	-----	-----	135.79	112.70	130.71	-----	-----	-----	-----	93.67

Table S7. Selected bond lengths (Å) of theoretically optimized structures (B3P86-D3/6-31+G(d),LanL2DZ(Ag) level of theory) and X-ray experimental structure in (partial) mechanism *M3*. Labels as in Table S3.

Compound	Mn1-C1	C1-N1	C1-N2	N2-H2	C1-H2	H2-O1	N2-Ag1	O1-Ag1	O1-Ag2	Mn1-N2
**1+Ag₂O**	2.083	1.341	1.341	1.006	-----	-----	-----	2.027	2.027	-----
**I11**	2.088	1.350	1.331	1.039	-----	1.753	-----	2.060	2.039	-----
**TS(11-12)**	2.101	1.373	1.354	1.031	-----	1.845		2.077	2.071	-----
**I12**	2.099	1.352	1.329	1.043	-----	1.702	-----	2.068	2.038	-----
**3+Ag₂O**	------	1.345	1.297	-----	1.086	-----	-----	2.027	2.027	2.098
**3 (*exp*)**	-----	1.356(4)	1.288(4)	-----	1.03(3)	-----	-----	-----	-----	2.084(3)

Table S8. Selected bond angles (°) of theoretically optimized structures (B3P86-D3/6-31+G(d),LanL2DZ(Ag) level of theory) and X-ray experimental structure in (partial) mechanism *M3*. Labels as in Table S4.

Compound	Mn1-C1-N1	Mn1-C1-N2	Mn1-N2-C1	Mn1-N2-C3	N1-C1-N2	N2-H2-O1	N2-Ag1-O1	Ag1-O1-Ag2
**1+Ag$_2$O**	128.24	118.35	-----	-----	113.39	------	-----	94.36
**I11**	125.96	120.64	-----	------	113.28	156.20	-----	104.90
**TS(11-12)**	126.60	120.46	-----	------	112.89	147.58	-----	107.28
**I12**	128.06	118.64	-----	------	113.02	163.09	-----	100.17
**3+Ag$_2$O**	------	-----	121.66	122.34	126.97	-----	-----	94.36
**3 (*exp*)**	-----	-----	123.0(2)	119.6(2)	125.6(3)	-----	-----	-----

Table S9. Standard Gibbs energies at room temperature (in kcal mol-1) for the *M1* mechanism calculated at the M06-D3(BJ)/6-311++G(2df,2pd),AQZP(Ag) level of theory. See Figure S1 for a graphic representation of the mechanism.

G^* (**1+Ag$_2$O**)	G^* (**I1**)	G^* (**TS1-2**)	G^* (**I2**)
0.0	–1.2	+7.9	–23.3
G^* (**TS2-3**)	G^* (**I3**)	G^* (**TS3-4**)	G^* (**I4**)
−11.1	–35.7	+11.5	–25.3
G^* (**TS4-5**)	G^* (**I5**)	G^* (**TS5-Prod**)	G^* (**2**+Ag$_2$O)
+15.2	+5.5	+16.7	–12.4

$G^0_{298}(\mathbf{x}) \equiv G^*(\mathbf{x}) - 8087350\ .5\, kcal\, mol^{-1}$.

Table S10. Standard Gibbs energies at room temperature (in kcal mol^{-1}) for the *M2* mechanism calculated at the M06-D3(BJ)/6-311++G(2df,2pd) level of theory. See Figure S2 for a graphic representation of the mechanism.

G^* **(1+Cu$_2$O)**	G^* **(I6)**	G^* **(TS6-7)**	G^* **(I7)**
0.0	–4.2	+1.2	–10.9
G^* **(TS7-8)**	G^* **(I8)**	G^* **(TS8-9)**	G^* **(I9)**
–3.0	–40.4	+11.7	–36.9
G^* **(TS9-10)**	G^* **(I10)**	G^* **(TS10-Prod)**	G^* (**2**+Cu$_2$O)
+7.2	–0.5	+16.5	–12.4

$G^0_{298}(\mathbf{x}) \equiv G^*(\mathbf{x}) - 3619796.4\, kcal\, mol^{-1}$.

Table S11. Standard Gibbs energies at room temperature (in kcal mol^{-1}) for the *M3* mechanism calculated at the M06-D3(BJ)/6-311++G(2df,2pd),AQZP(Ag) level of theory. See Figure S3 for a graphic representation of the mechanism.

G^* **(1+Ag$_2$O)**	G^* **(I11)**	G^* **(TS11-12)**	G^* **(I12)**	G^* **(3+Ag$_2$O)**
0.0	–0.8	+12.8	–1.7	–11.9

$G^0_{298}(\mathbf{x}) \equiv G^*(\mathbf{x}) - 8087350\ .5\, kcal\, mol^{-1}$.

Figure S1. Graphic representations of compounds in mechanism M1.

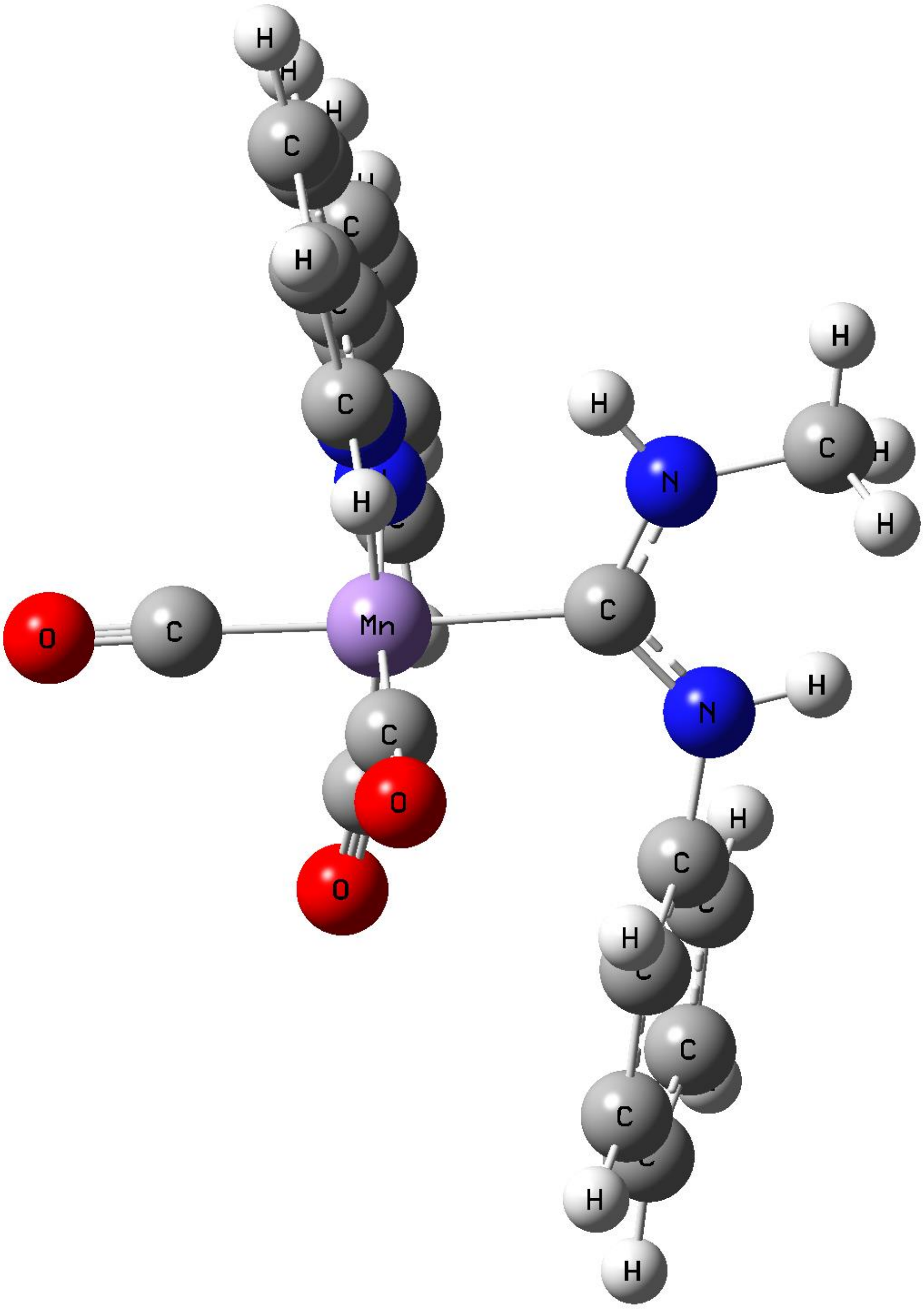

(a) Reactant **1**.

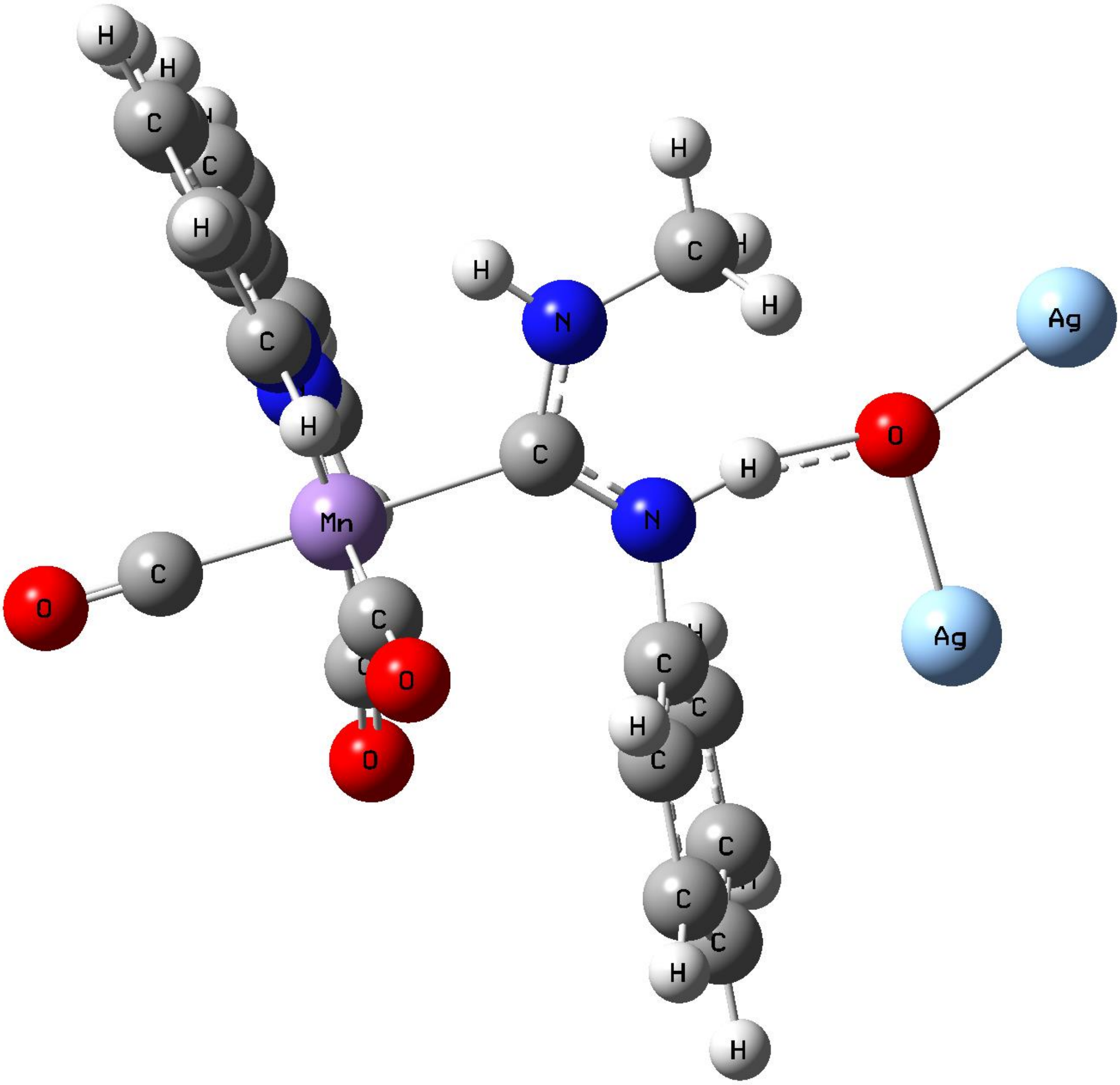

(b) Intermediate **I1**.

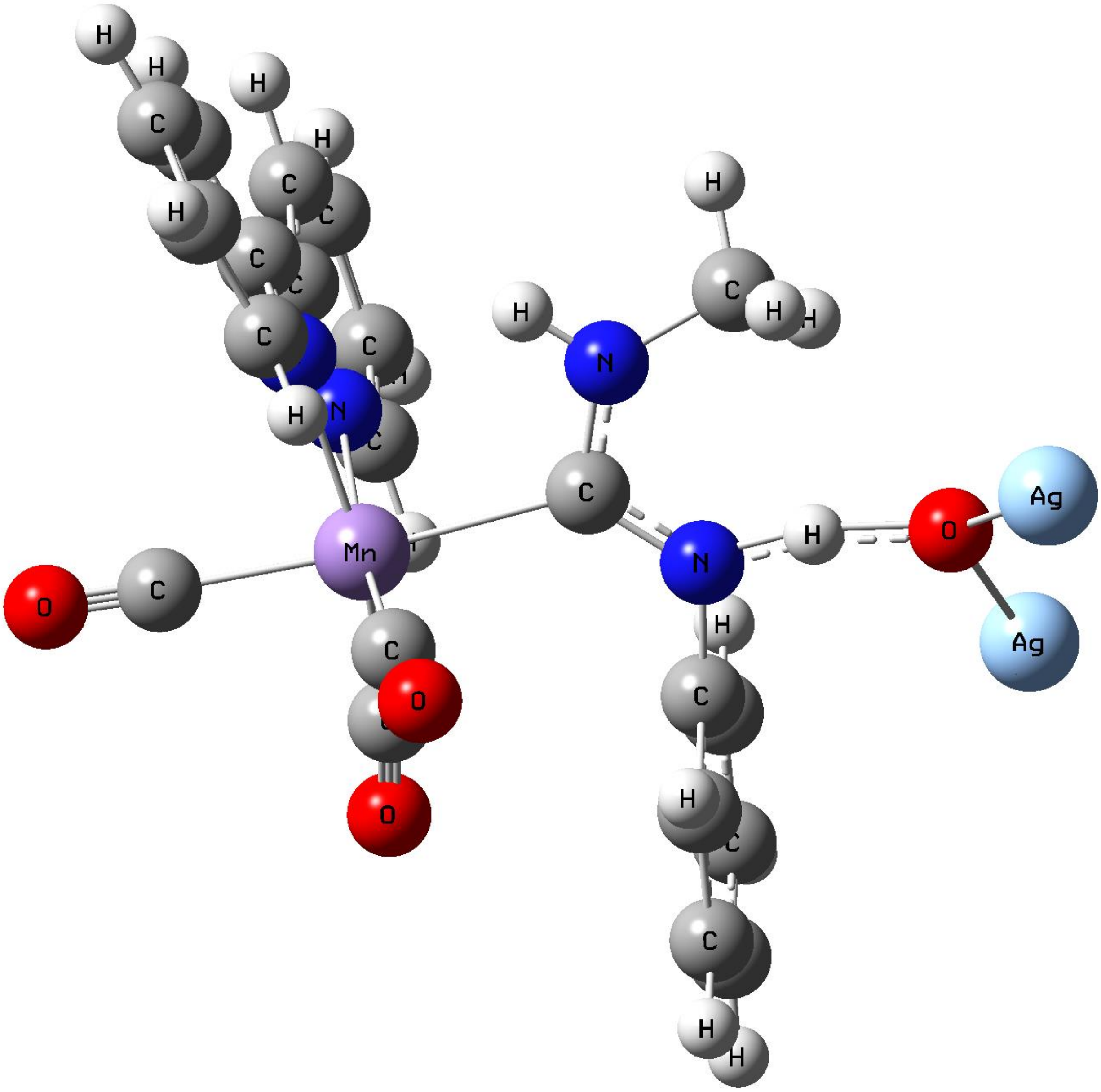

(c) Transition State **TS(1-2)**.

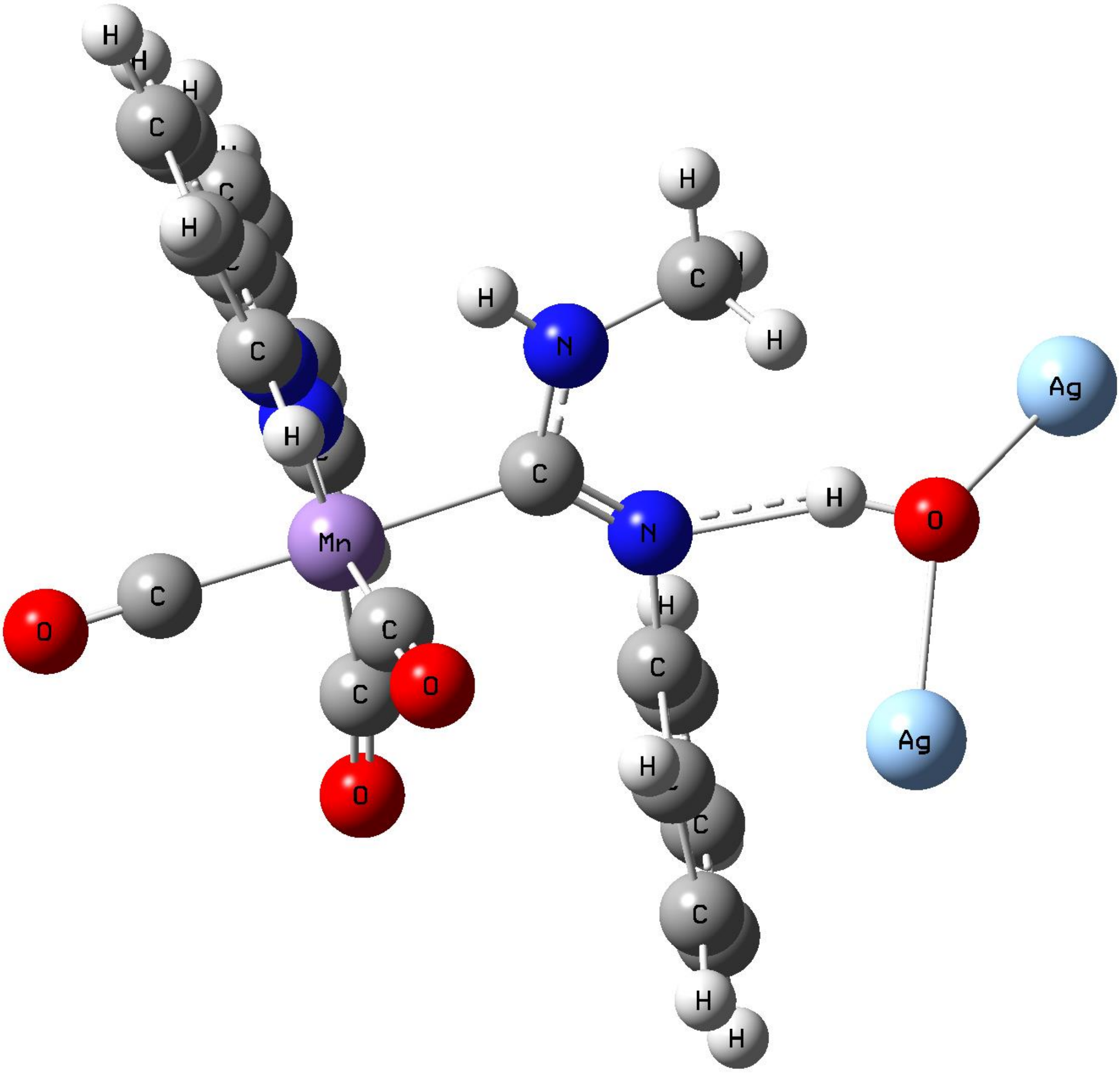

(d) Intermediate **I2**.

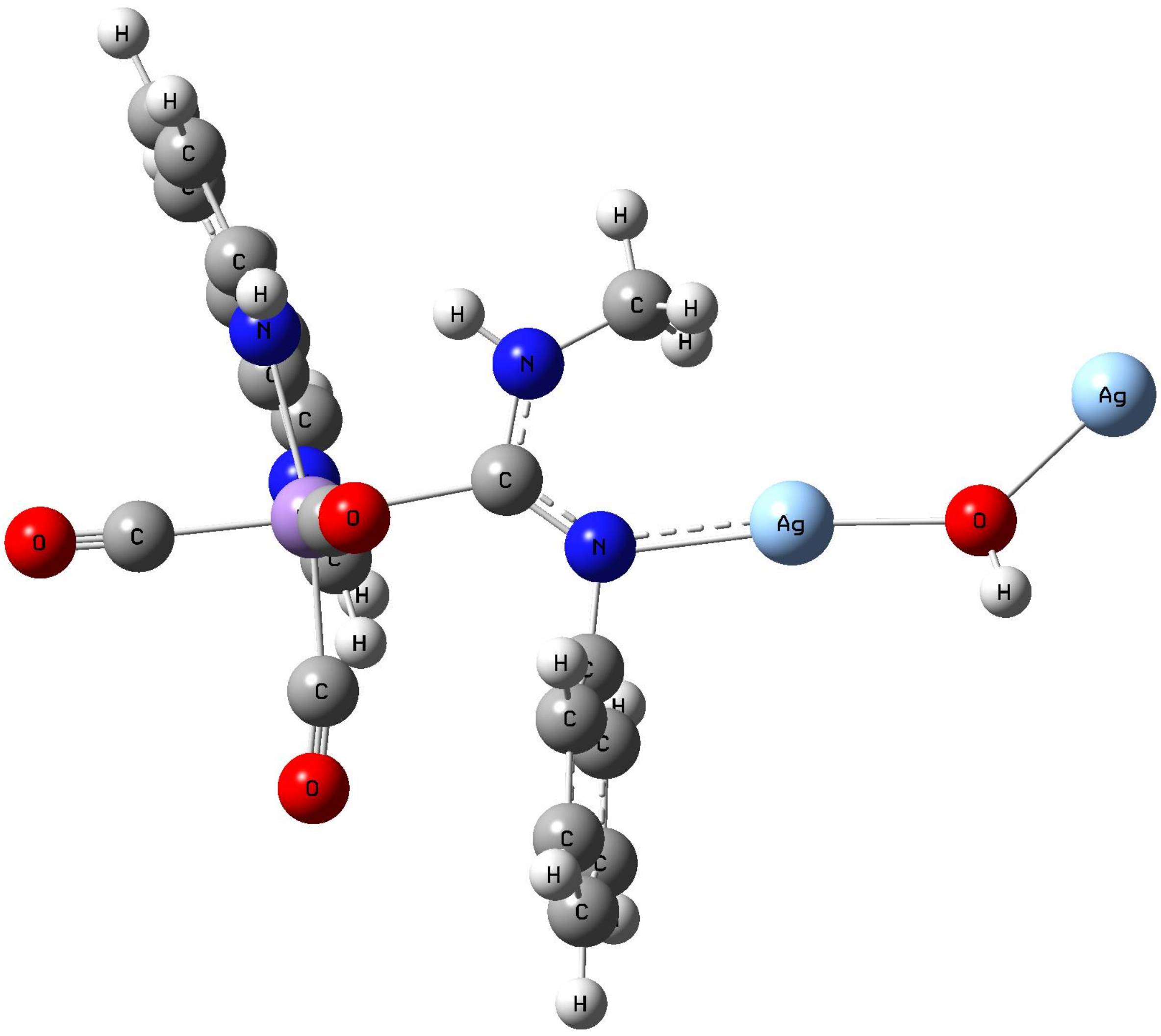

(e) Transition State **TS(2-3)**.

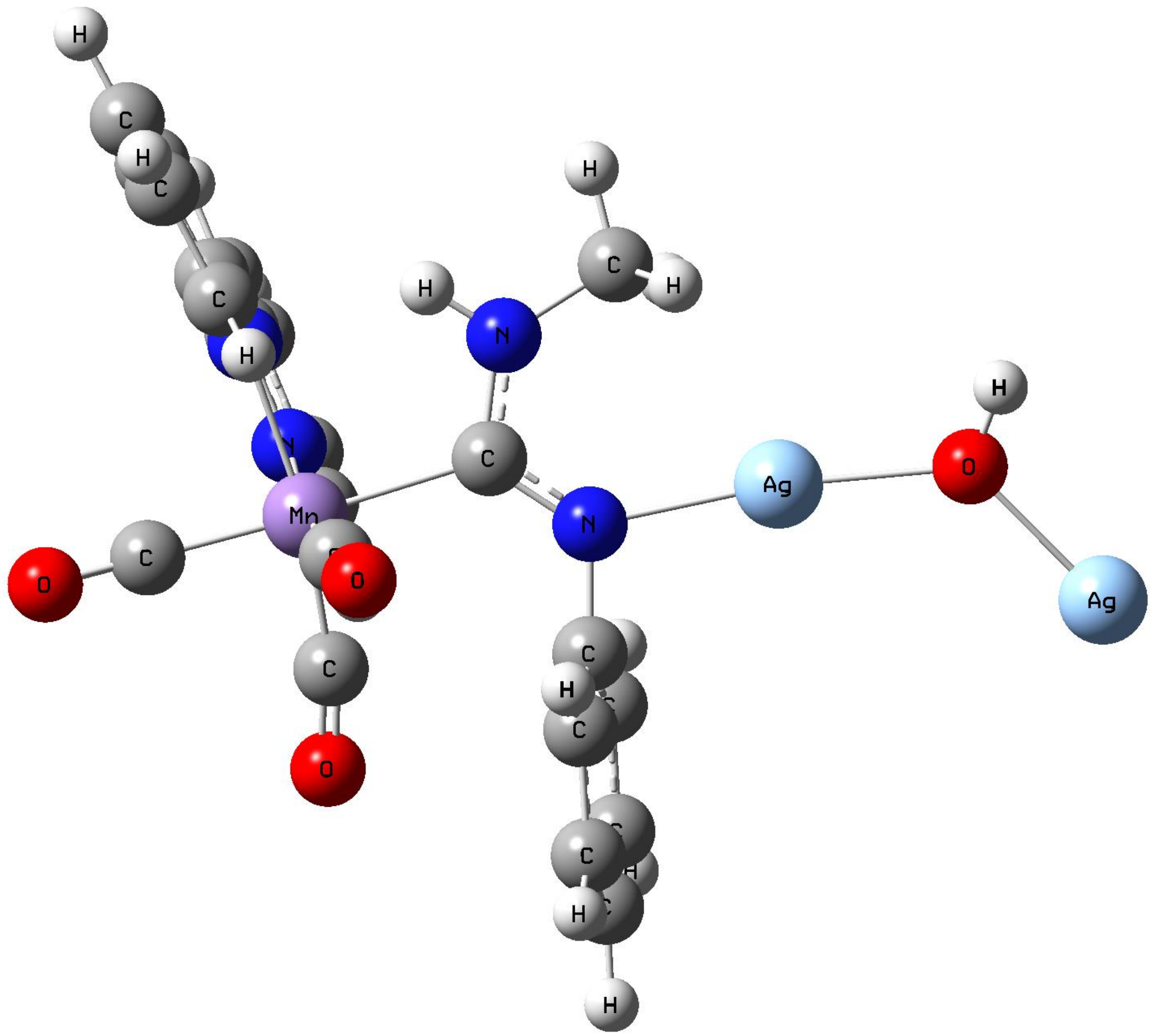

(f) Intermediate **I3**.

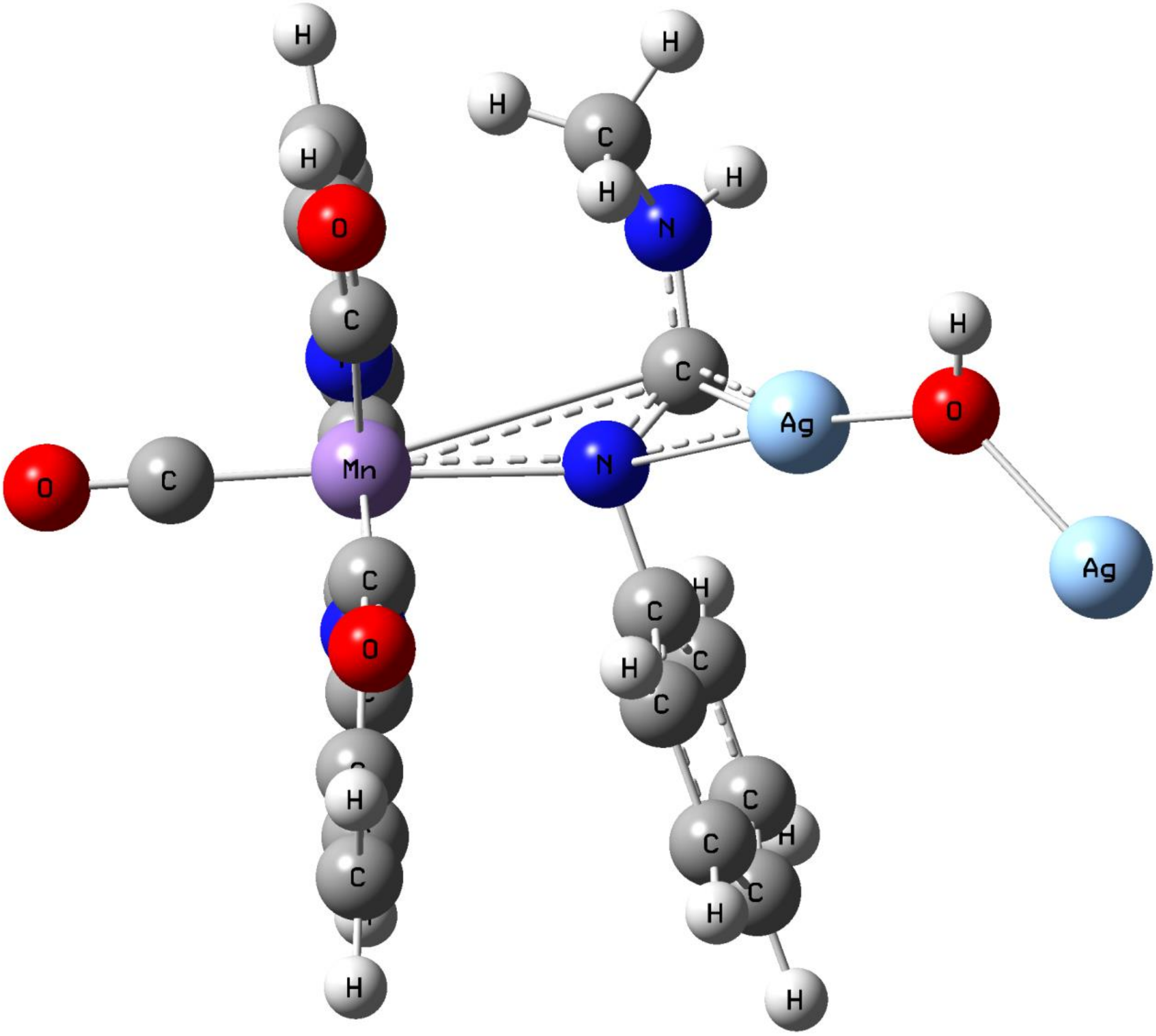

(g) Transition State **TS(3-4)**.

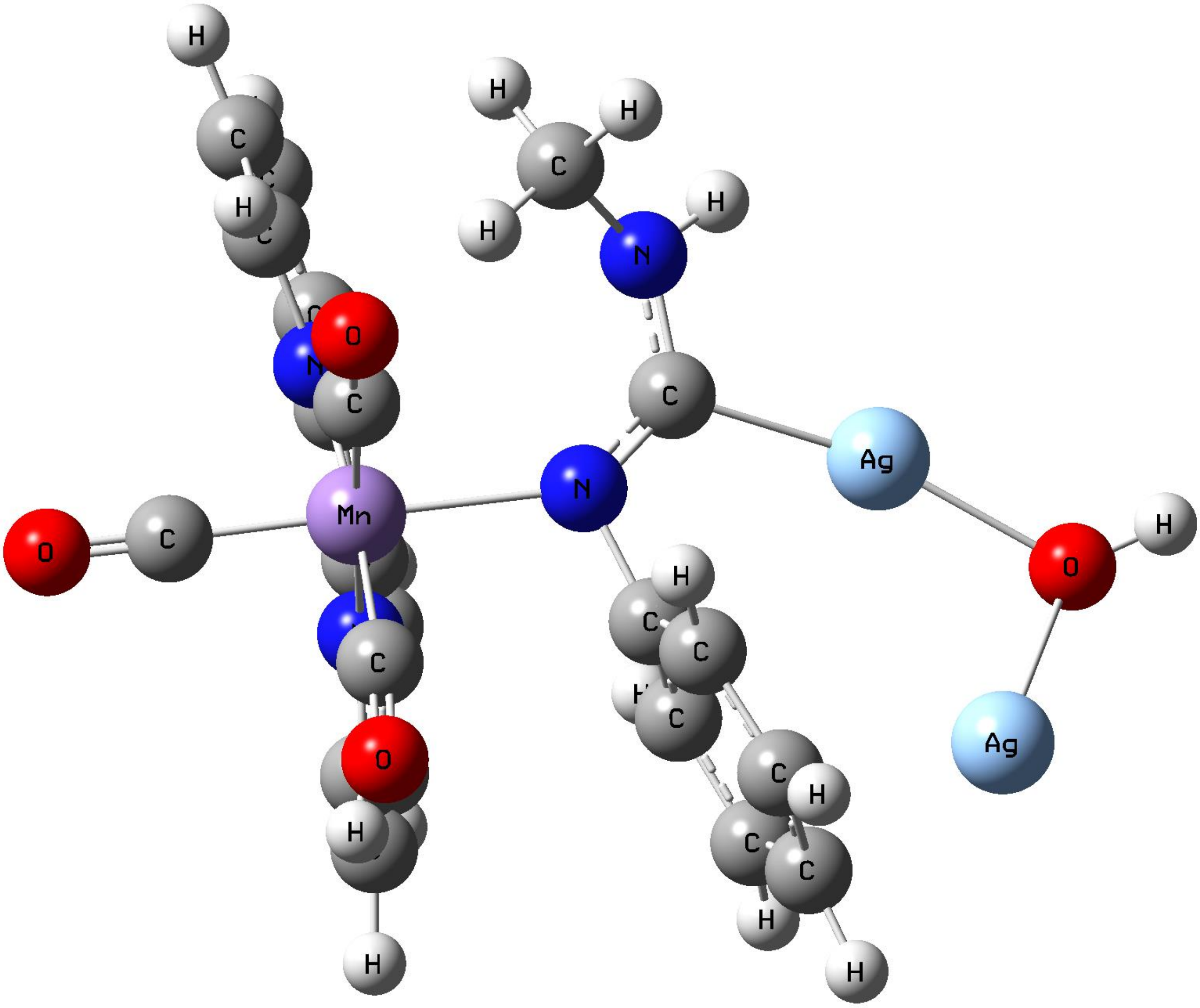

(h) Intermediate **I4**.

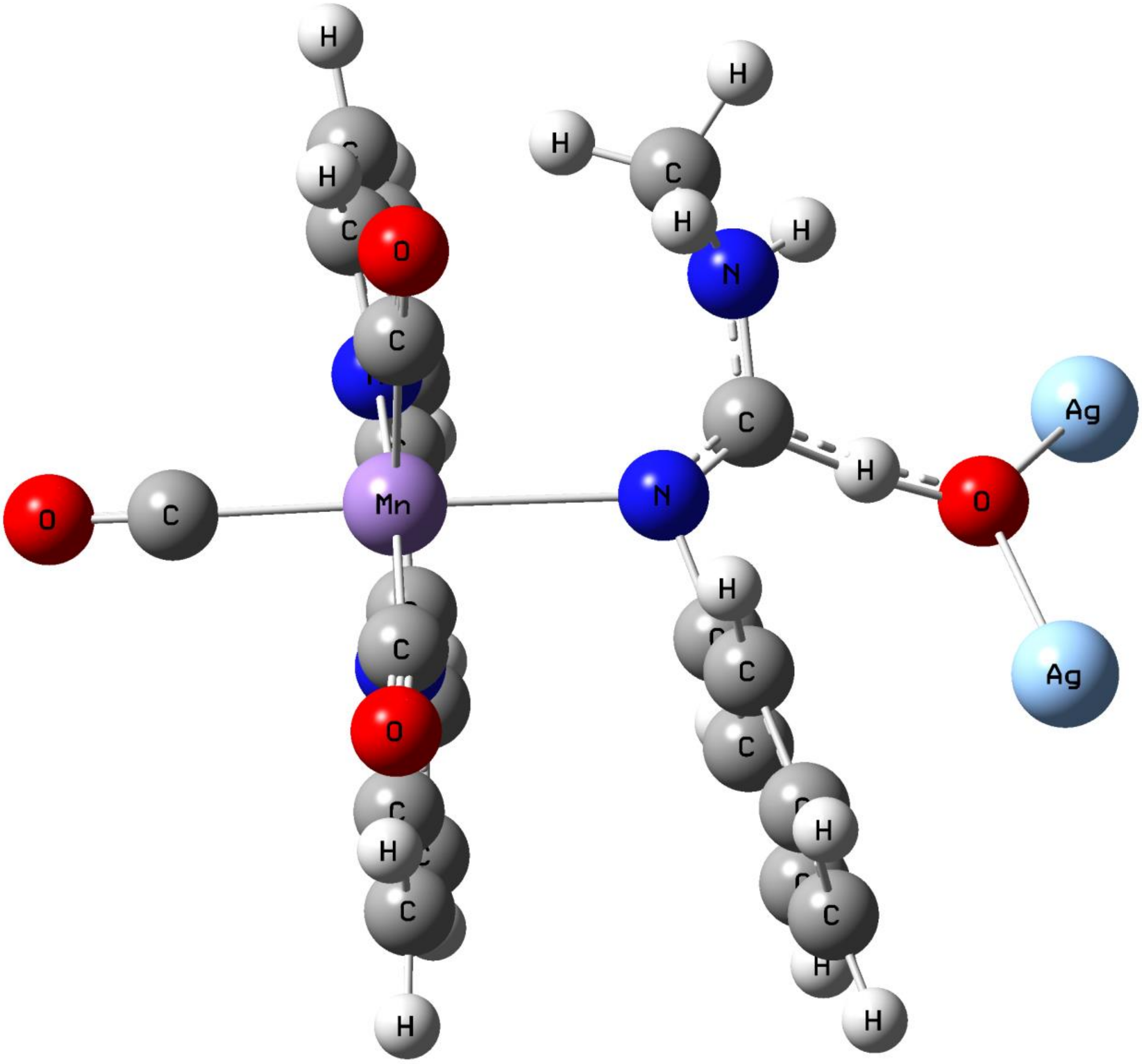

(i) Transition State **TS(4-5)**.

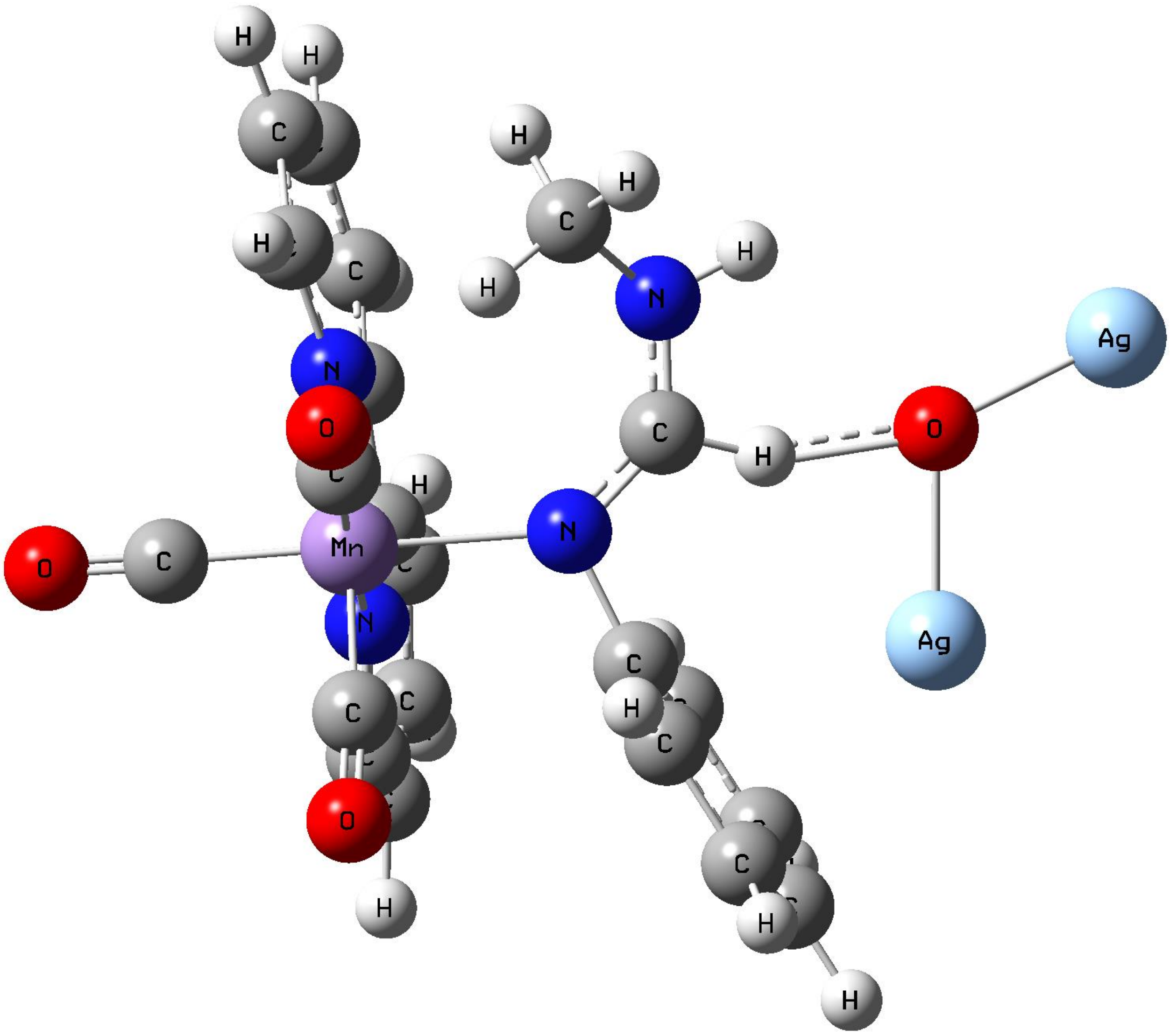

(j) Intermediate **I5**.

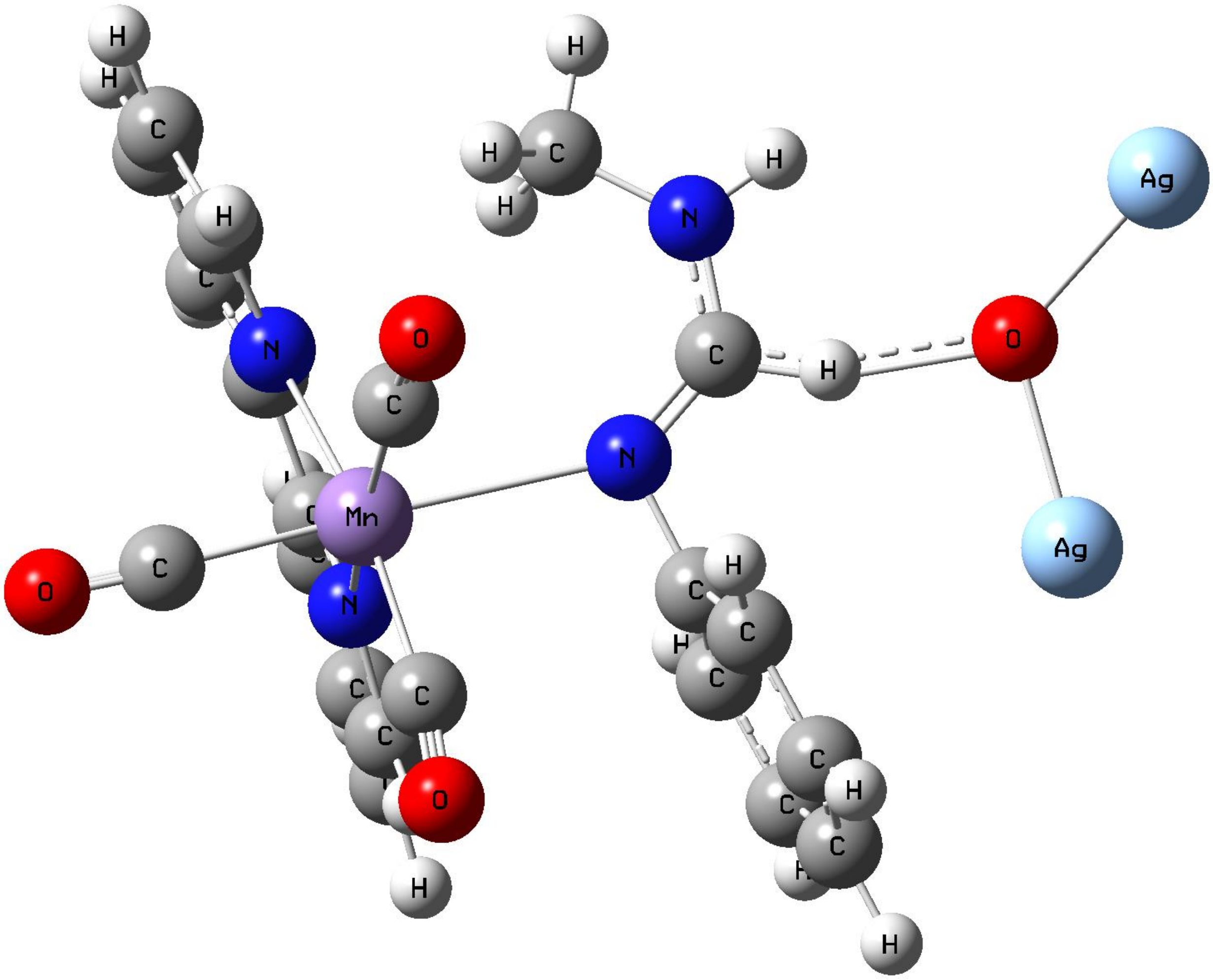

(k) Transition State **TS(5-Products)**.

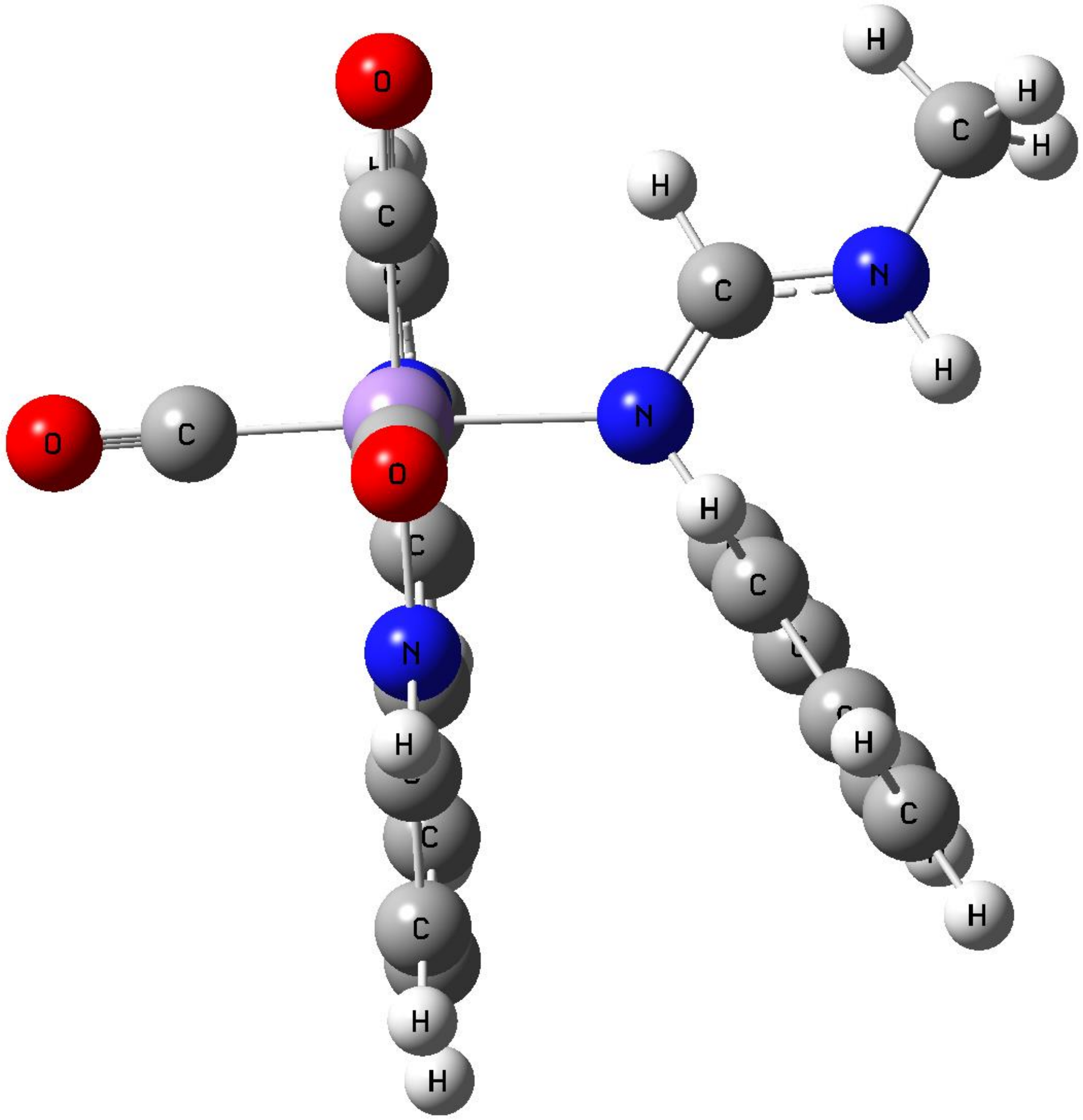

(l) Product **2**.

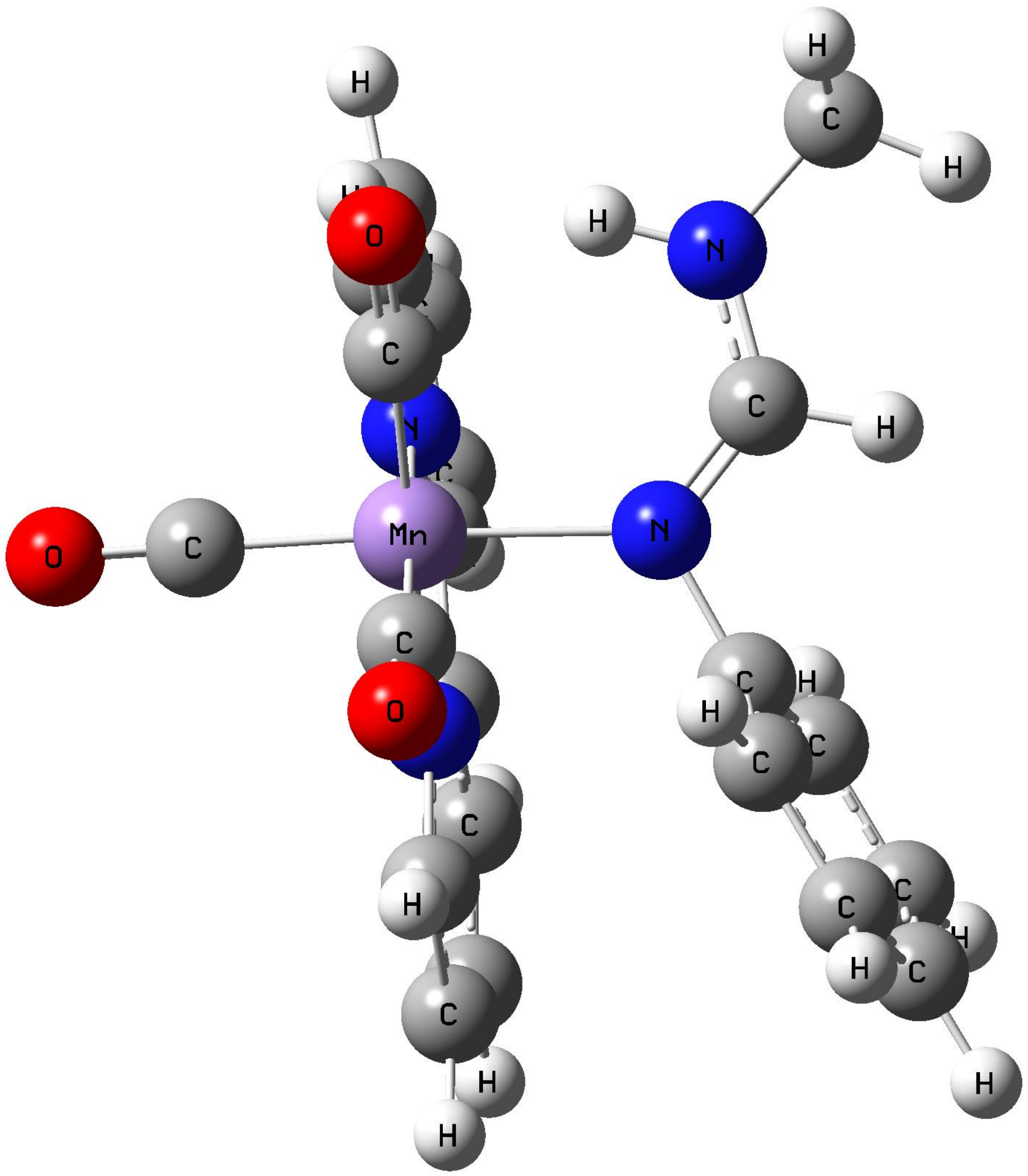

(m) Alternative Product **2′**.

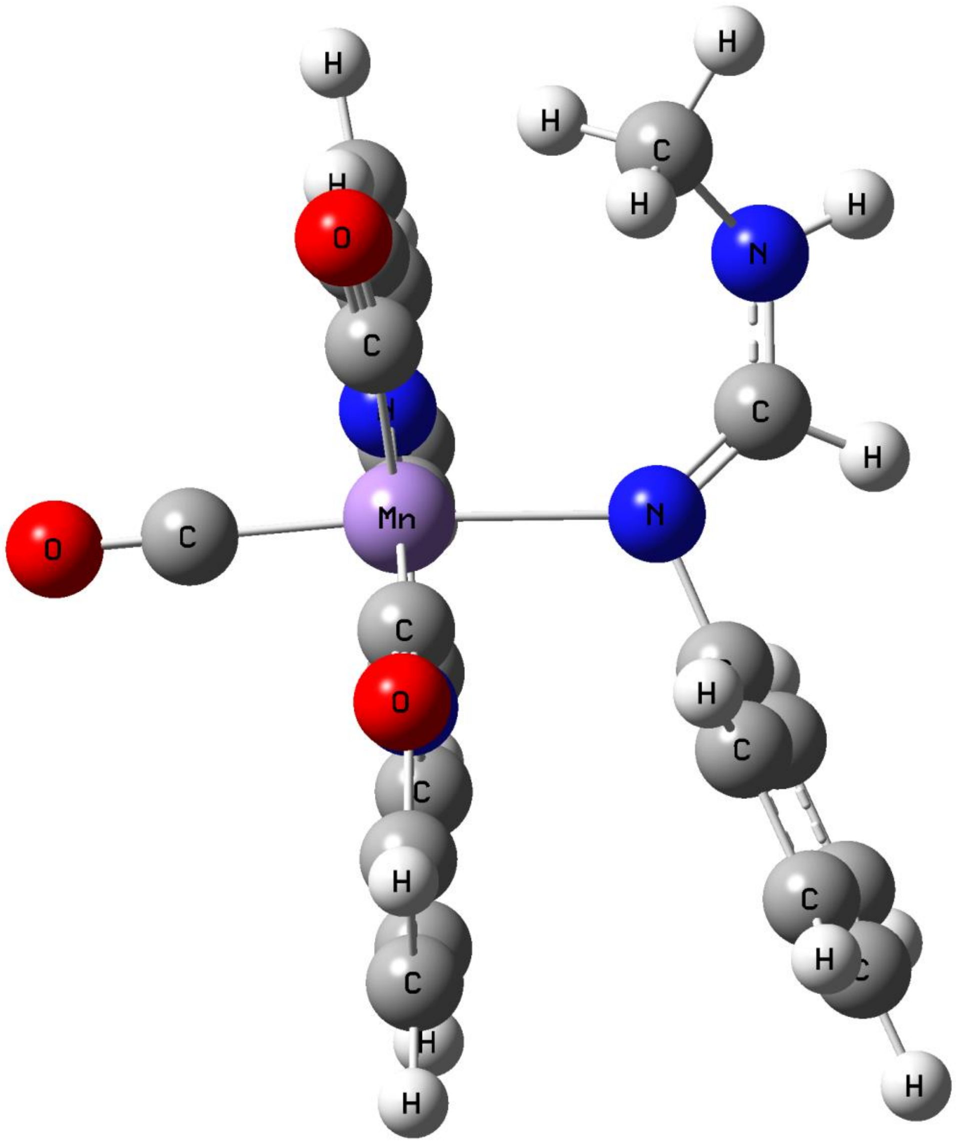

(n) Alternative Product **2′′**.

Figure S2. Graphic representations of compounds in mechanism M2.

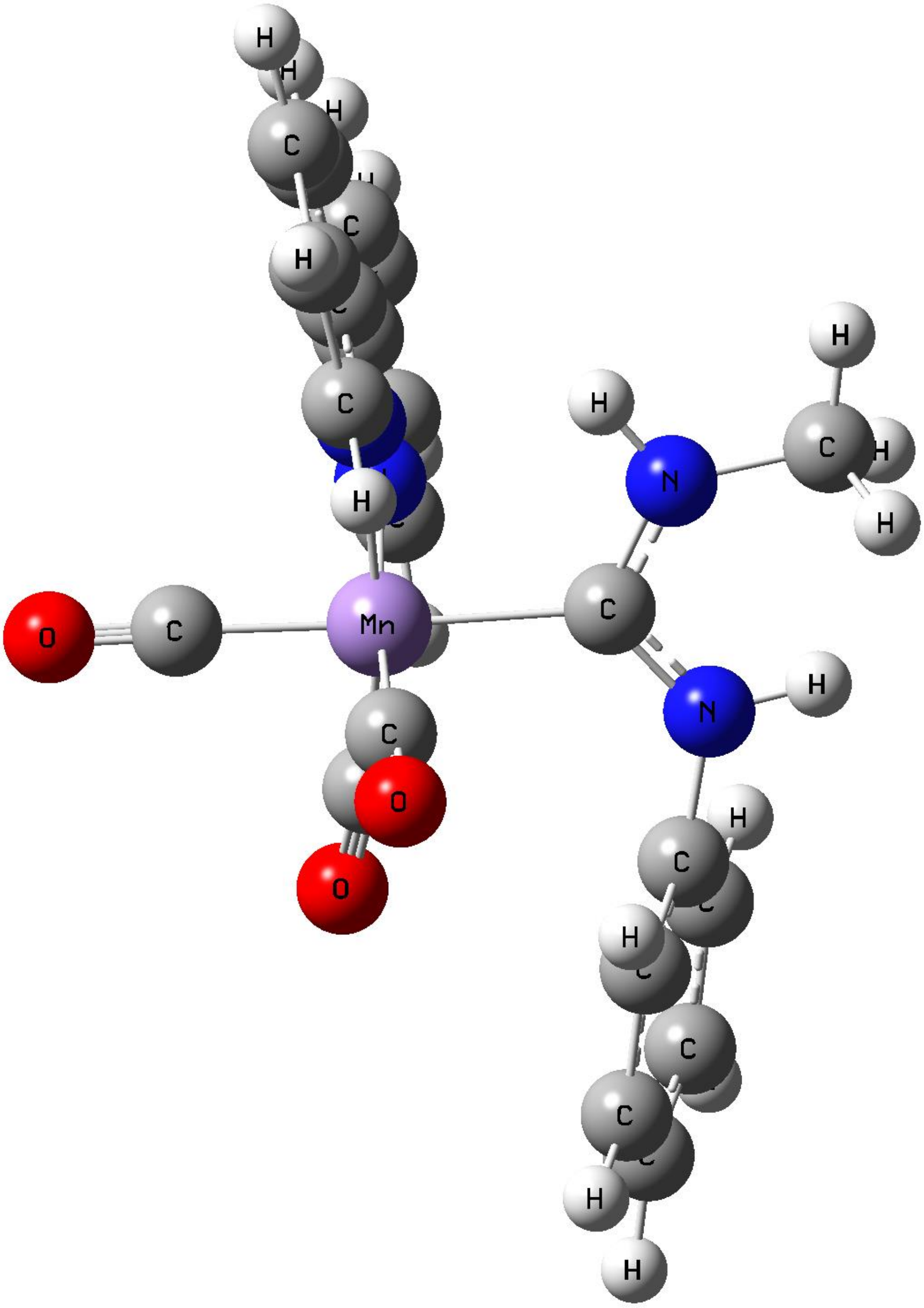

(a) Reactant **1**.

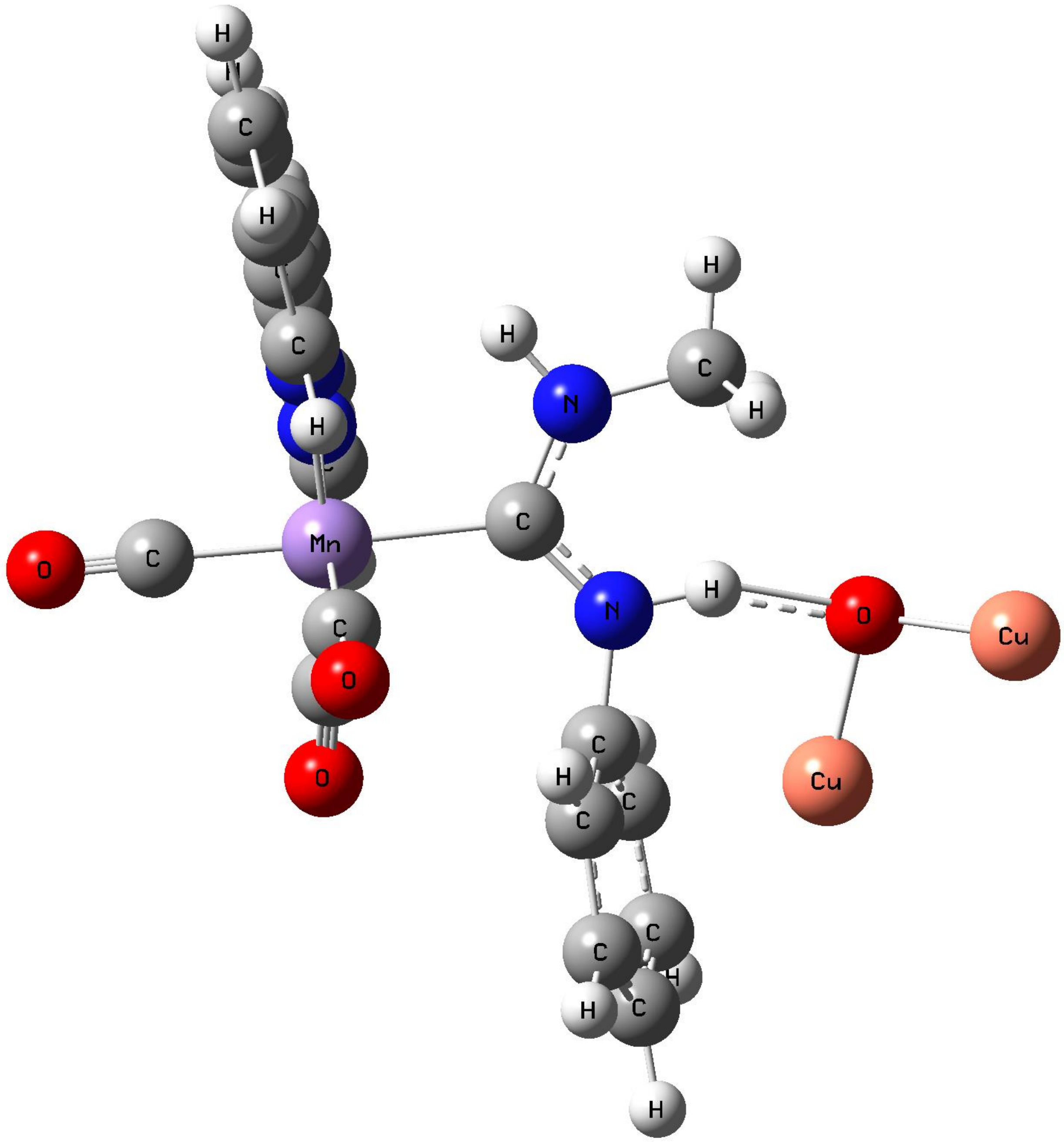

(b) Intermediate **I6**.

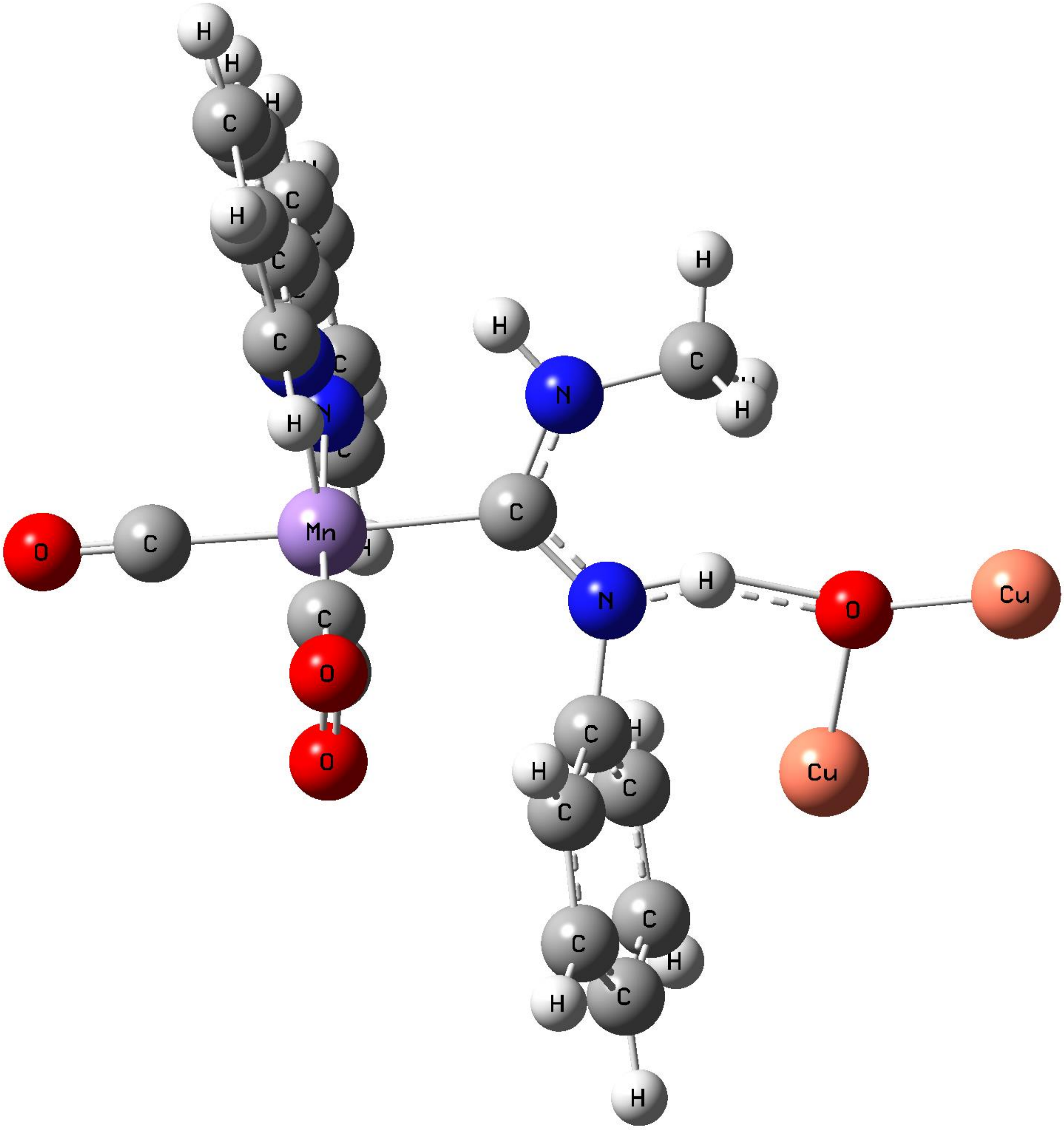

(c) Transition State **TS(6-7)**.

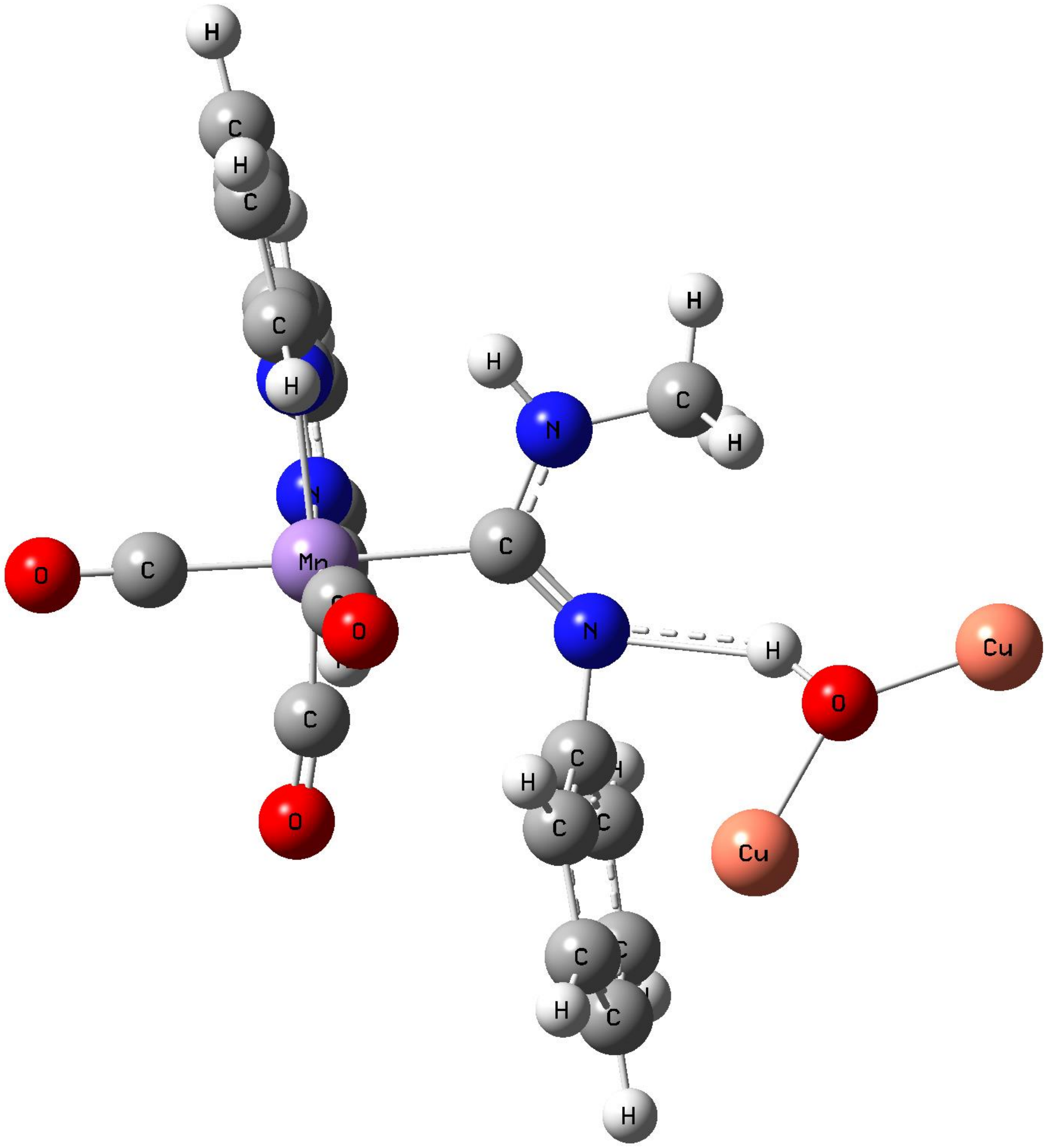

(d) Intermediate **I7**.

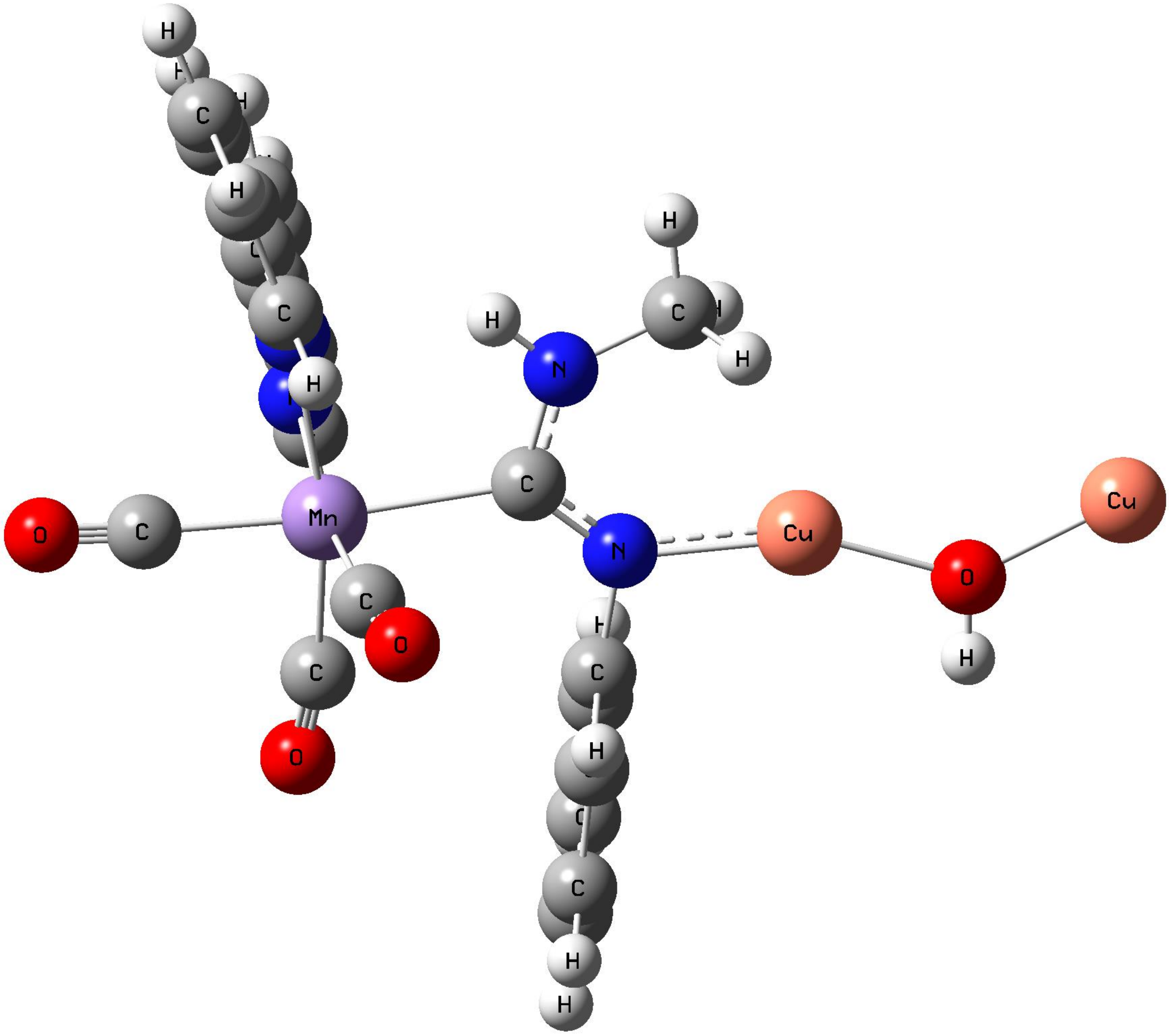

(e) Transition State **TS(7-8)**.

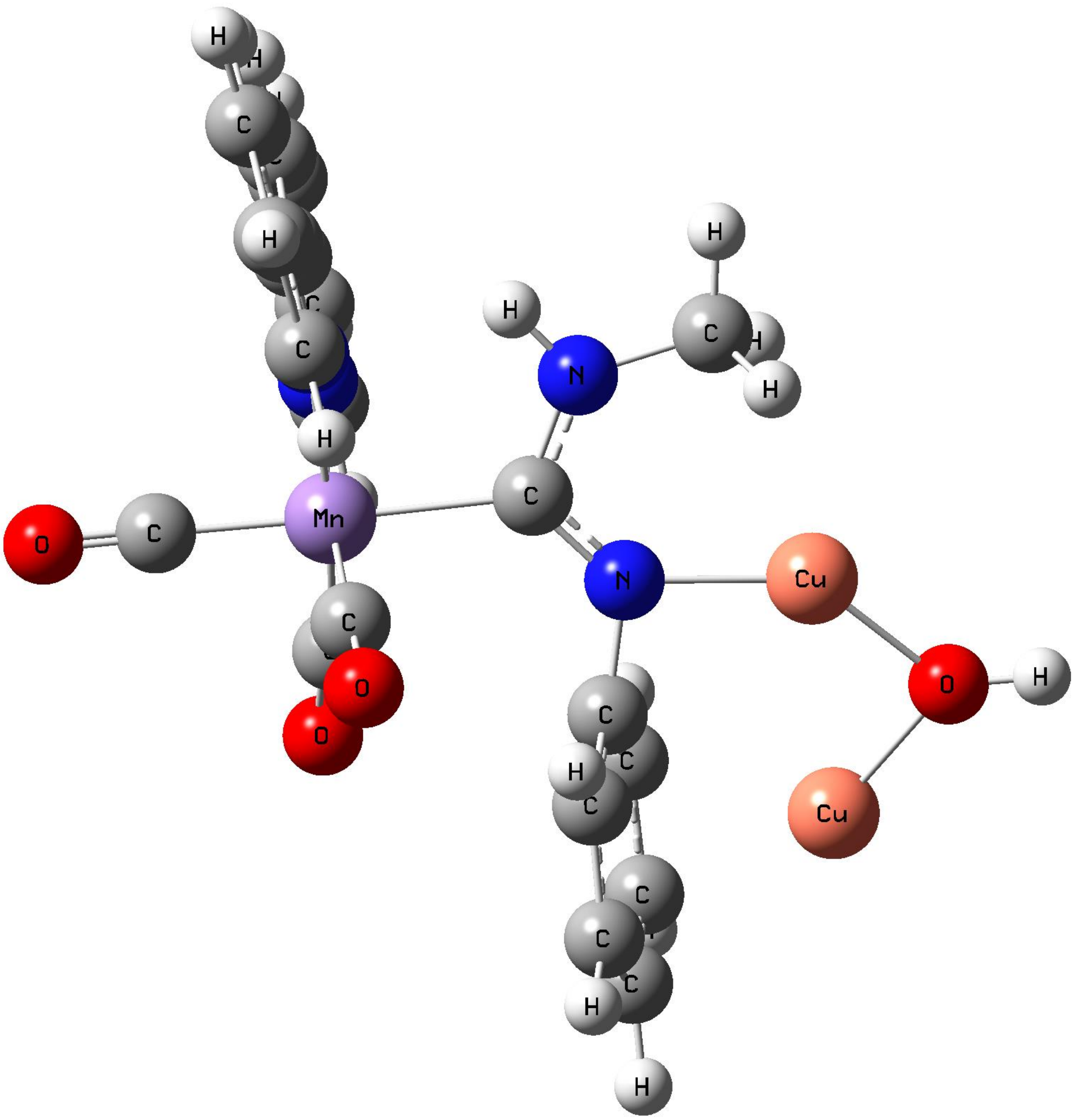

(f) Intermediate **I8**.

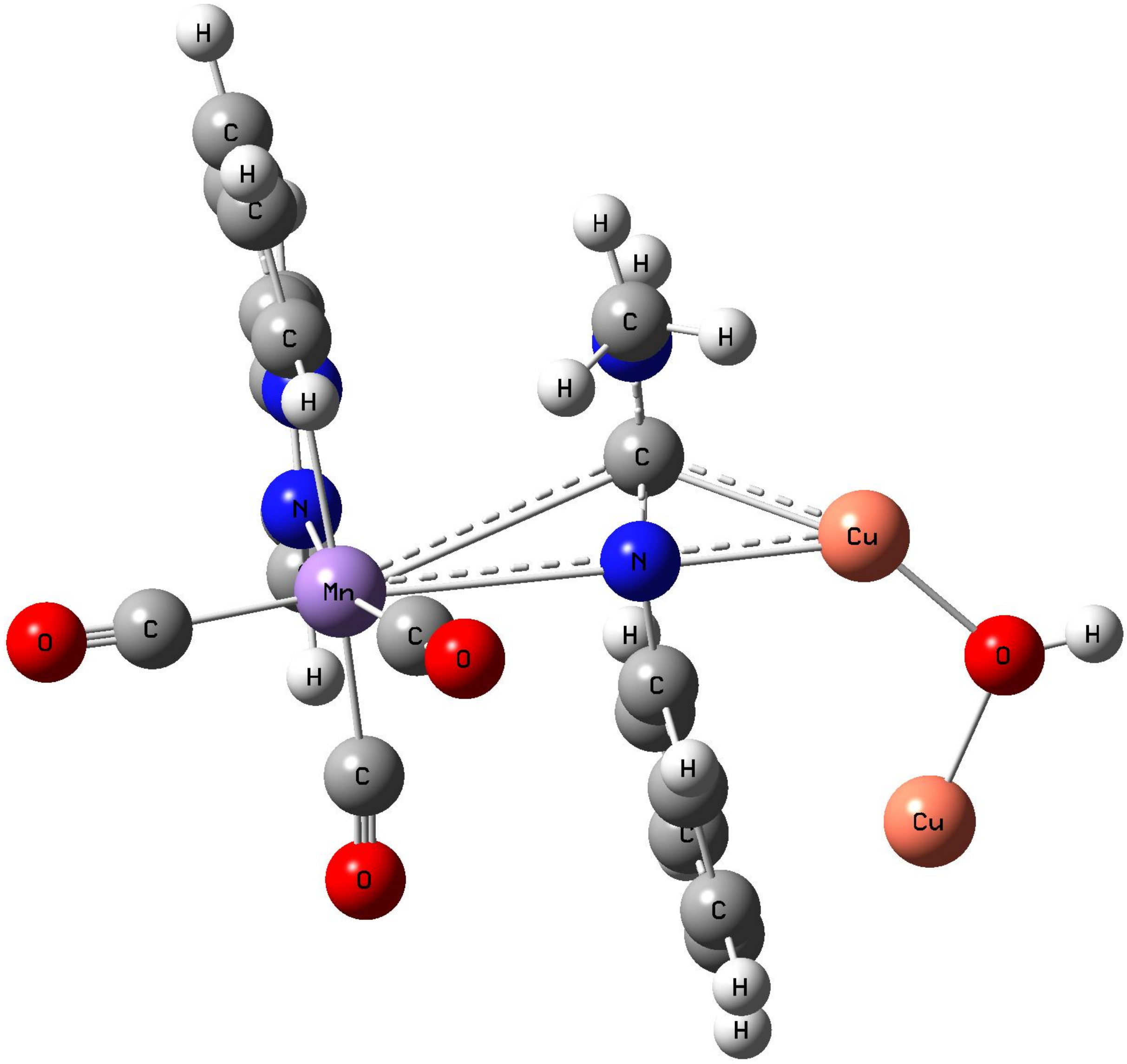

(g) Transition State **TS(8-9)**.

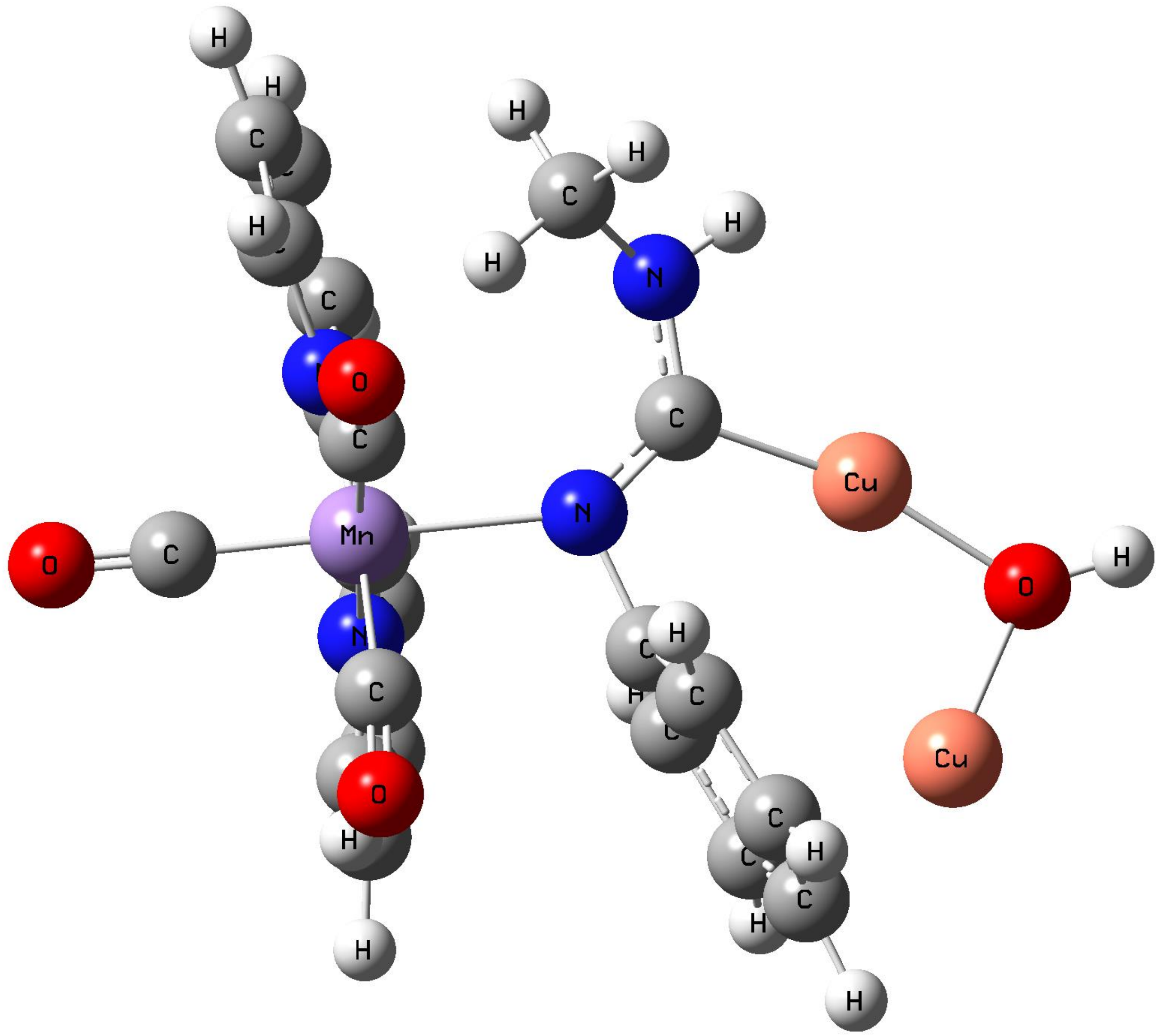

(h) Intermediate **I9**.

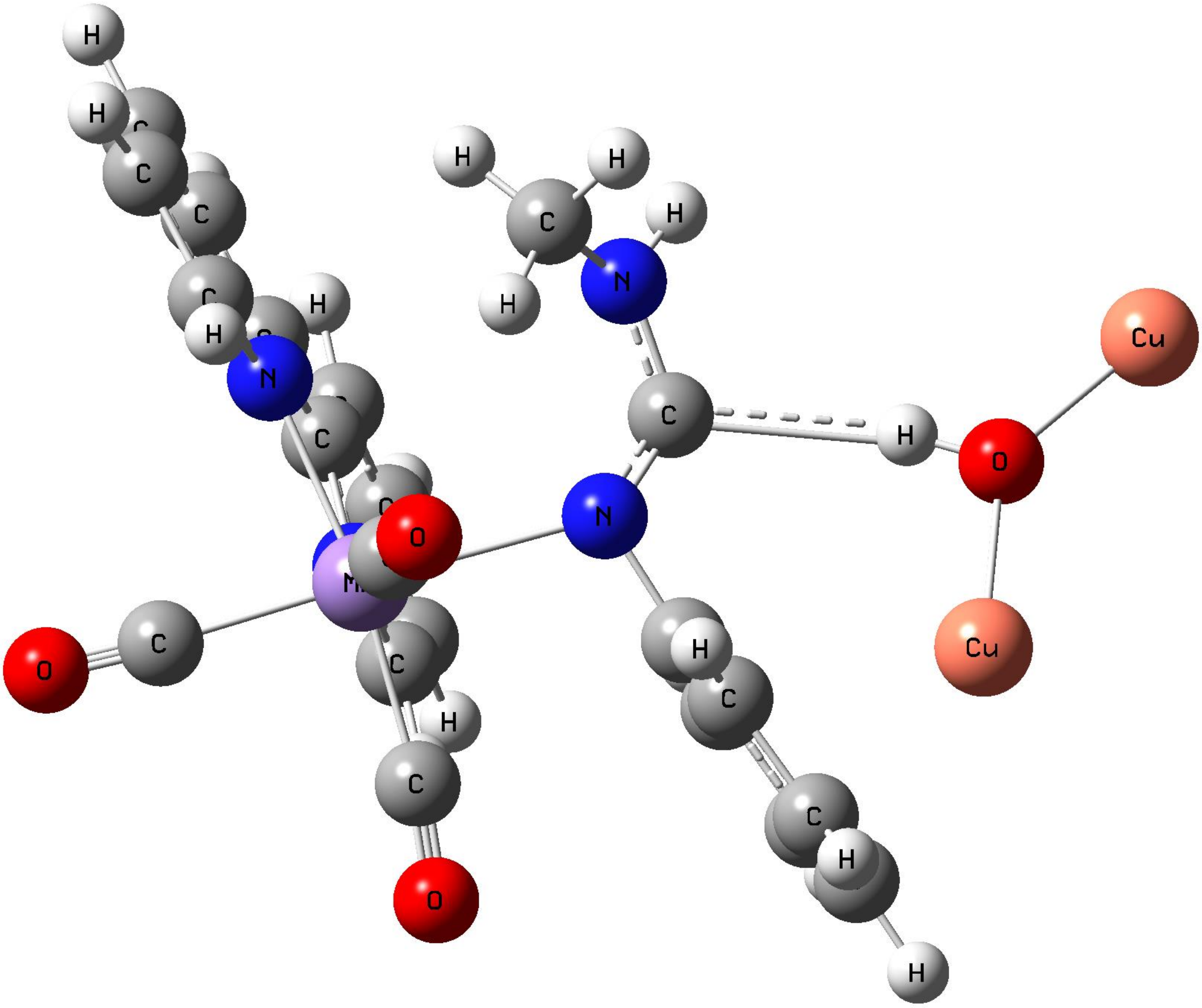

(i) Transition State **TS(9-10)**.

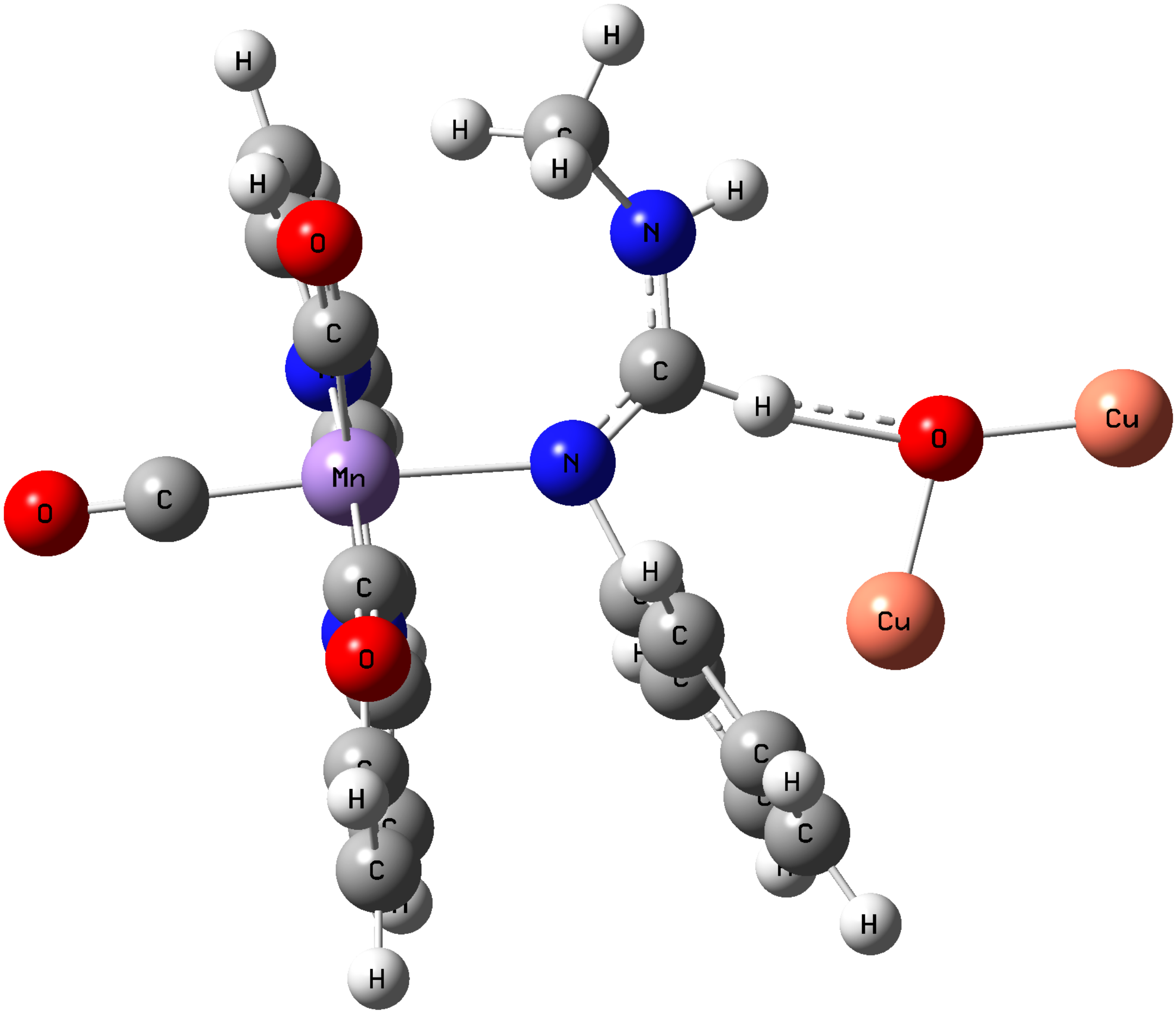

(j) Intermediate **I10**.

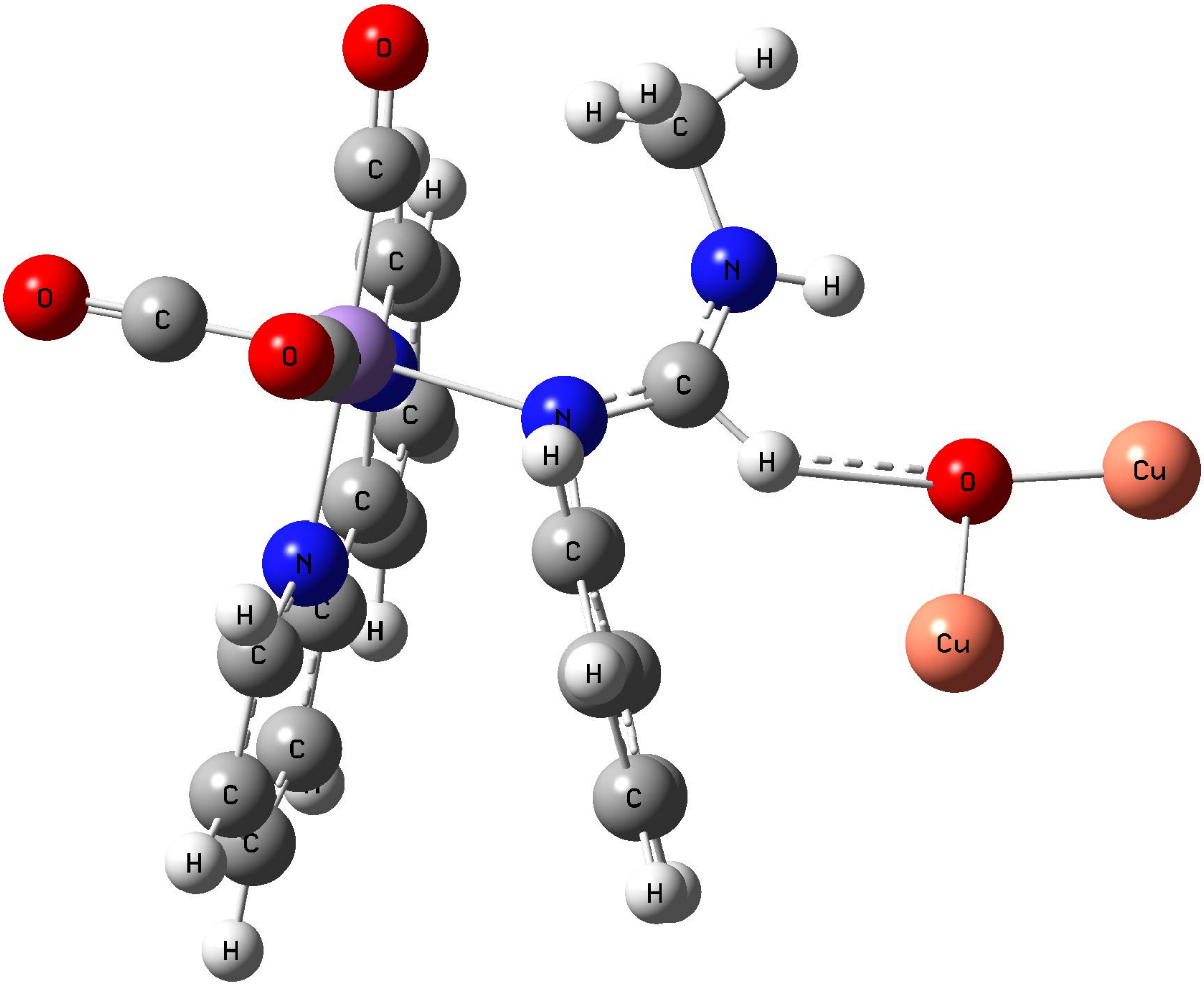

(k) Transition State **TS(10-Products)**.

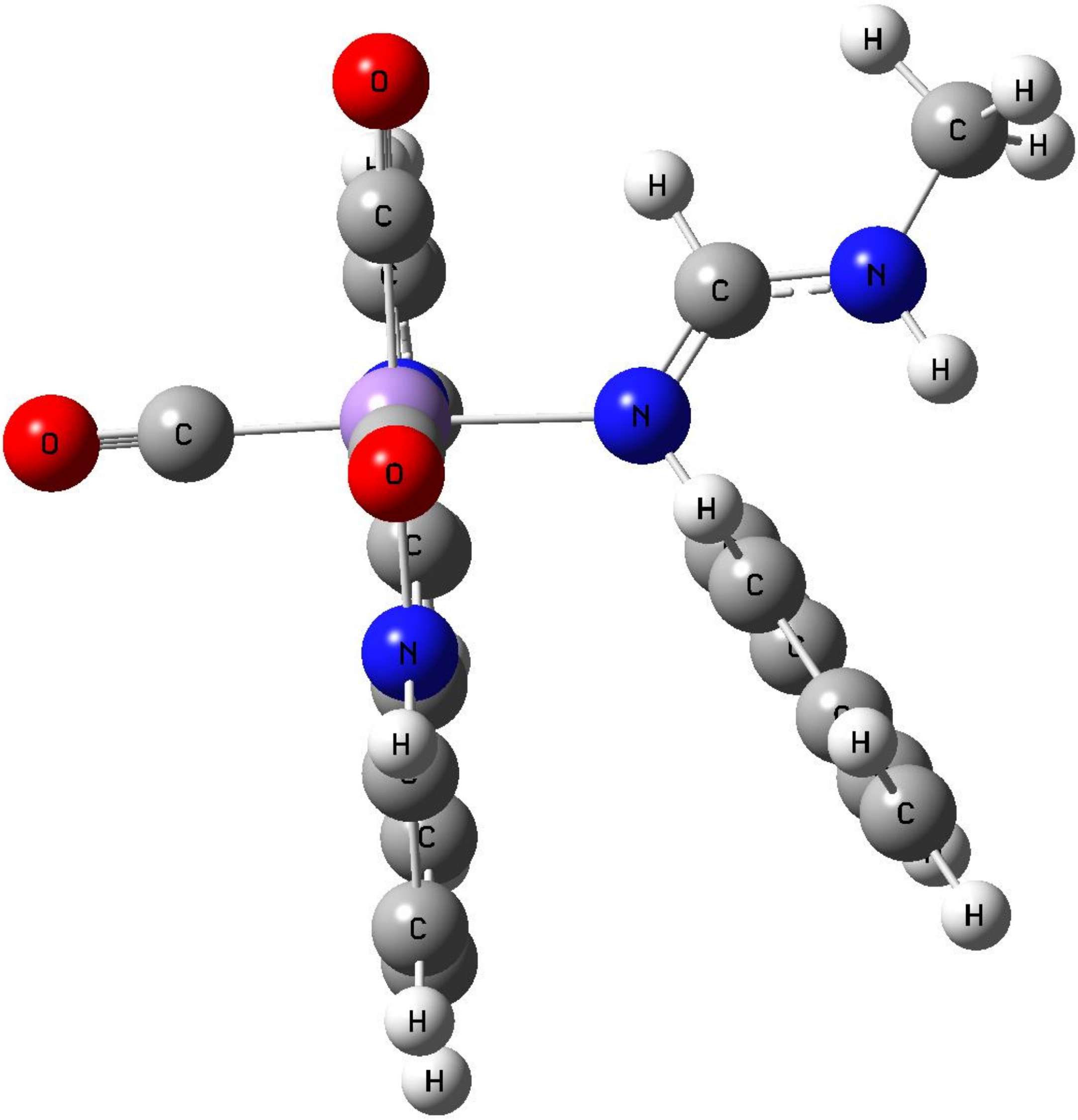

(l) Product **2**.

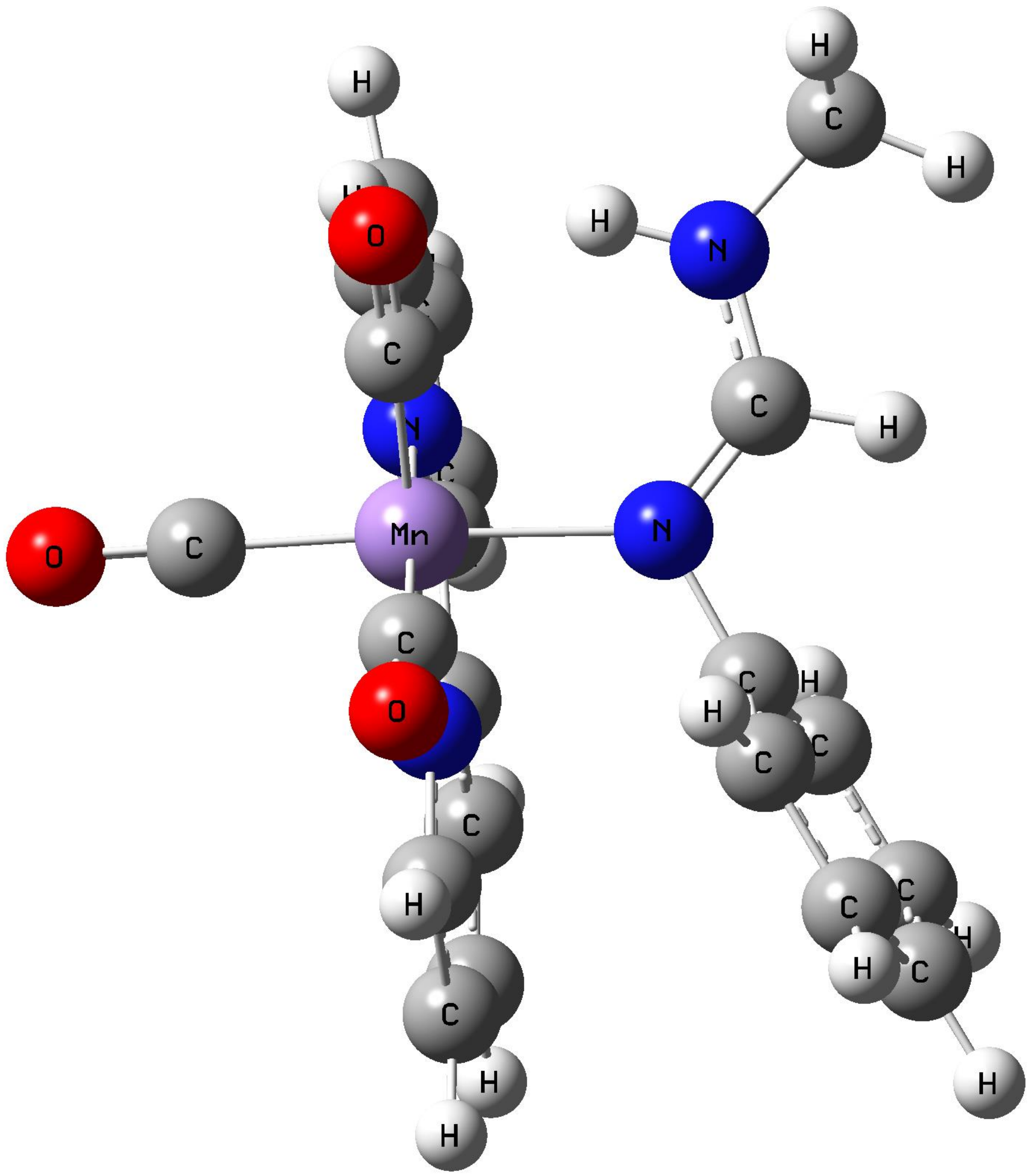

(m) Alternative Product **2′**.

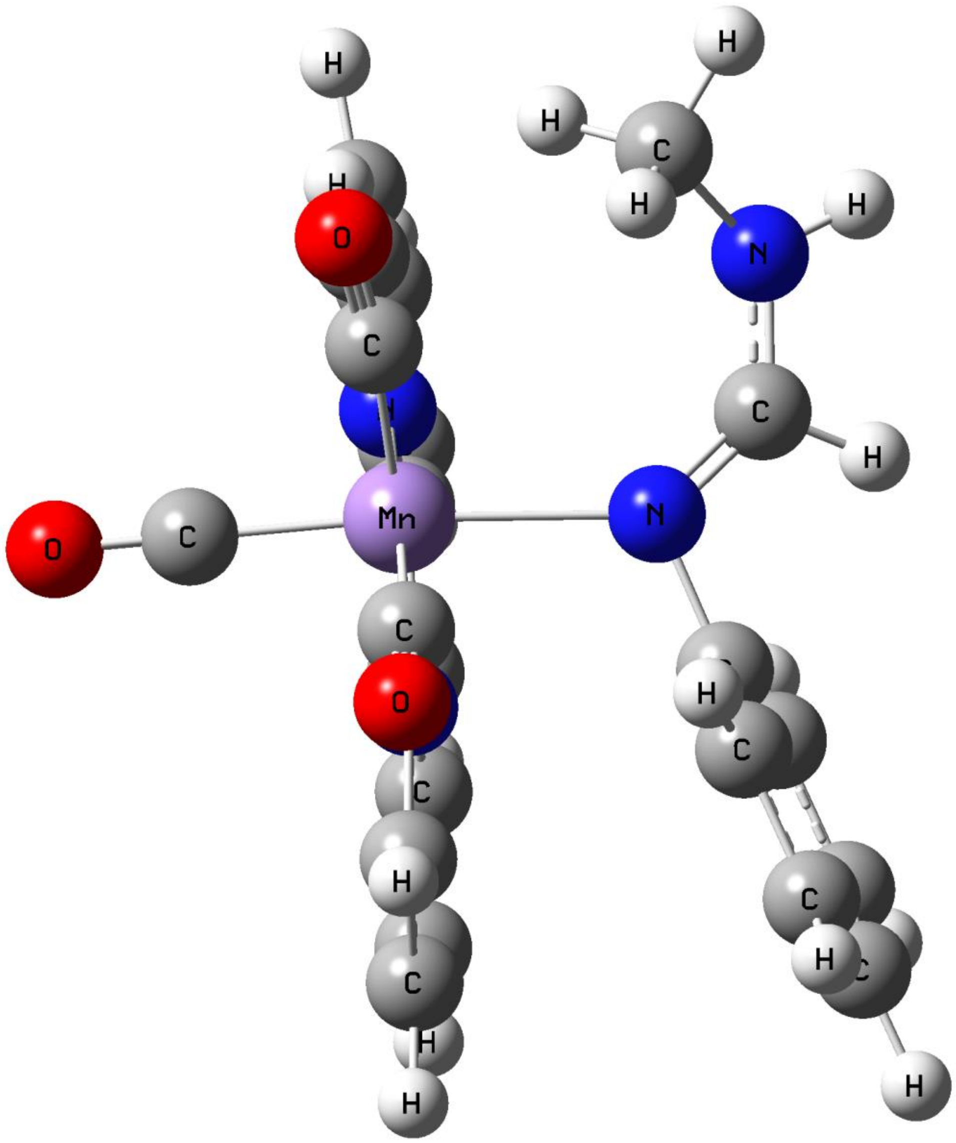

(n) Alternative Product **2′′**.

Figure S3. Graphic representations of compounds in (partial) mechanism M3.

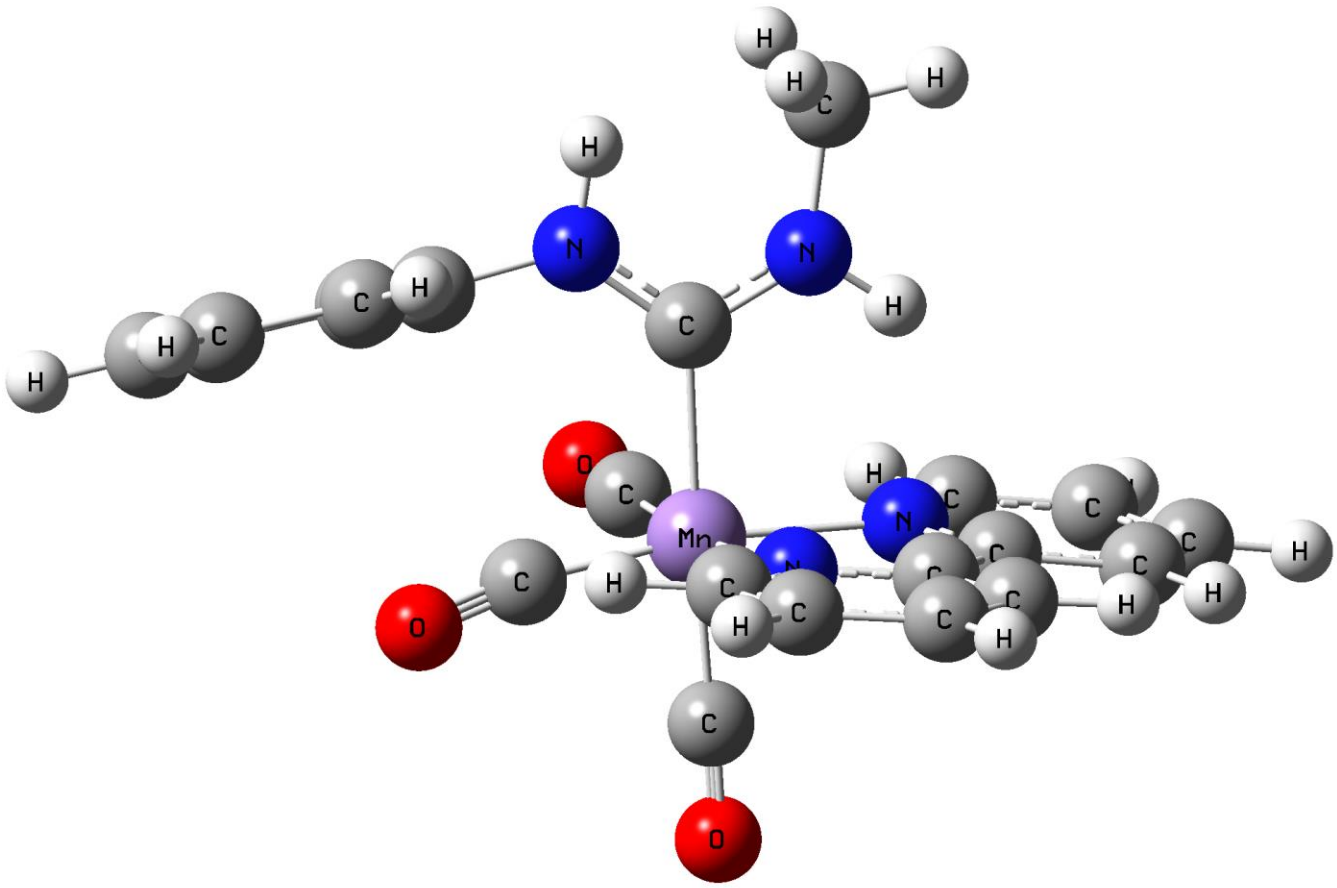

(a) Reactant **1**.

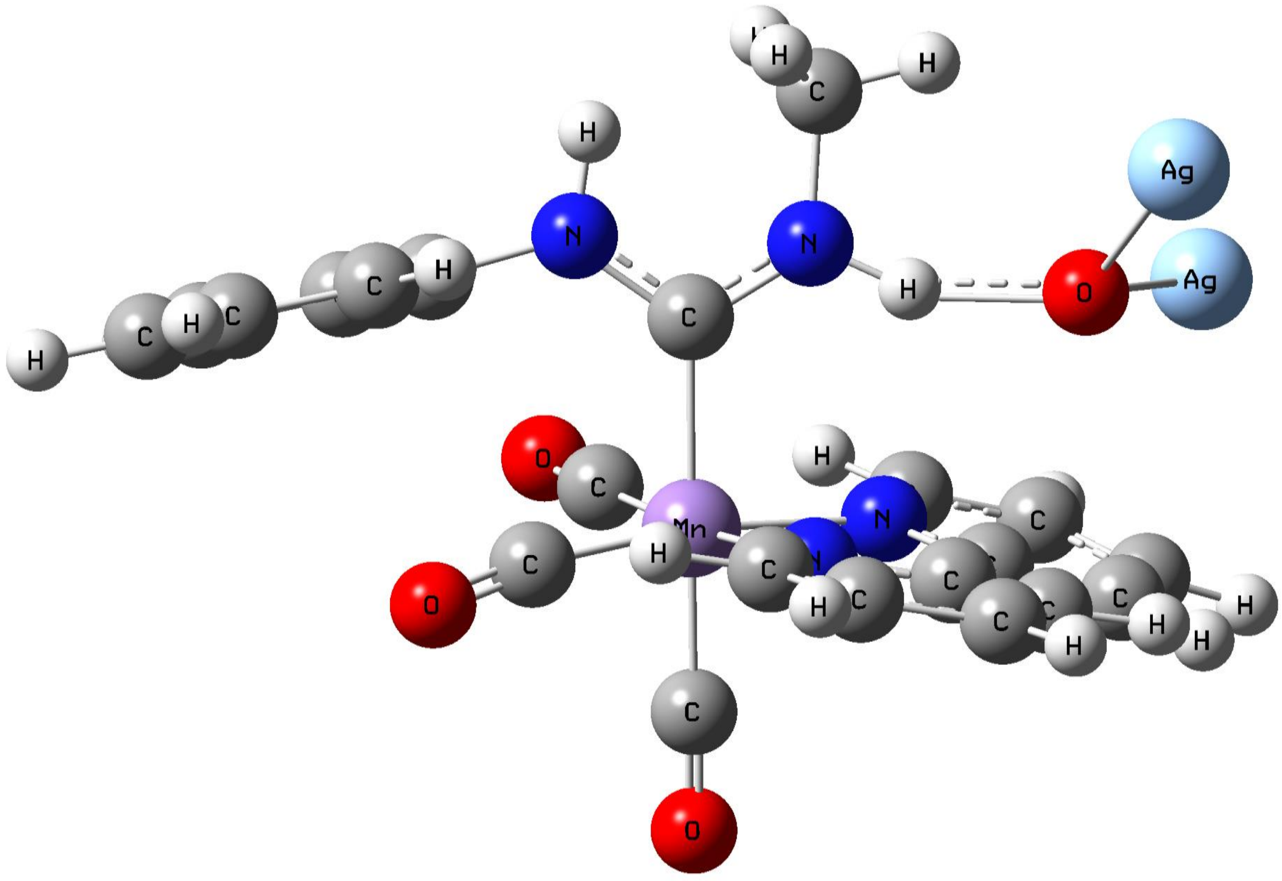

(b) Intermediate **I11**.

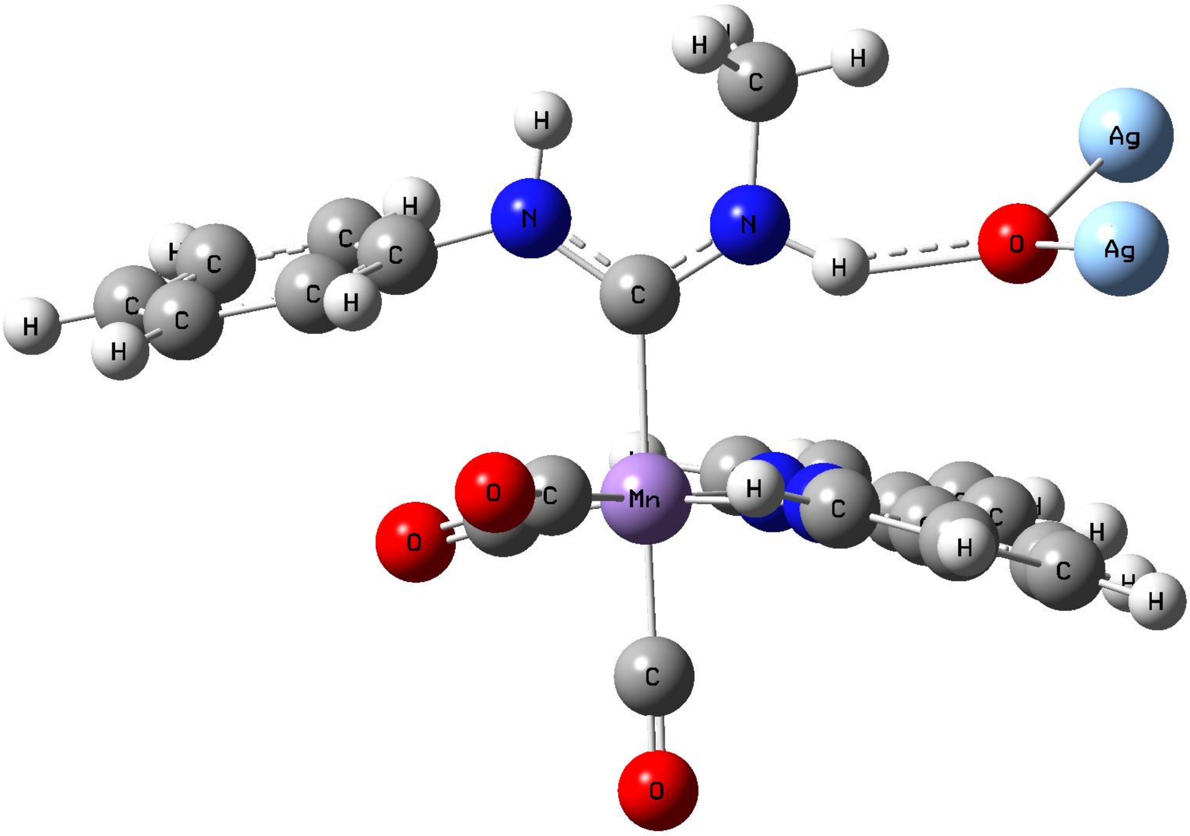

(**c**) Transition State **TS(11-12)**.

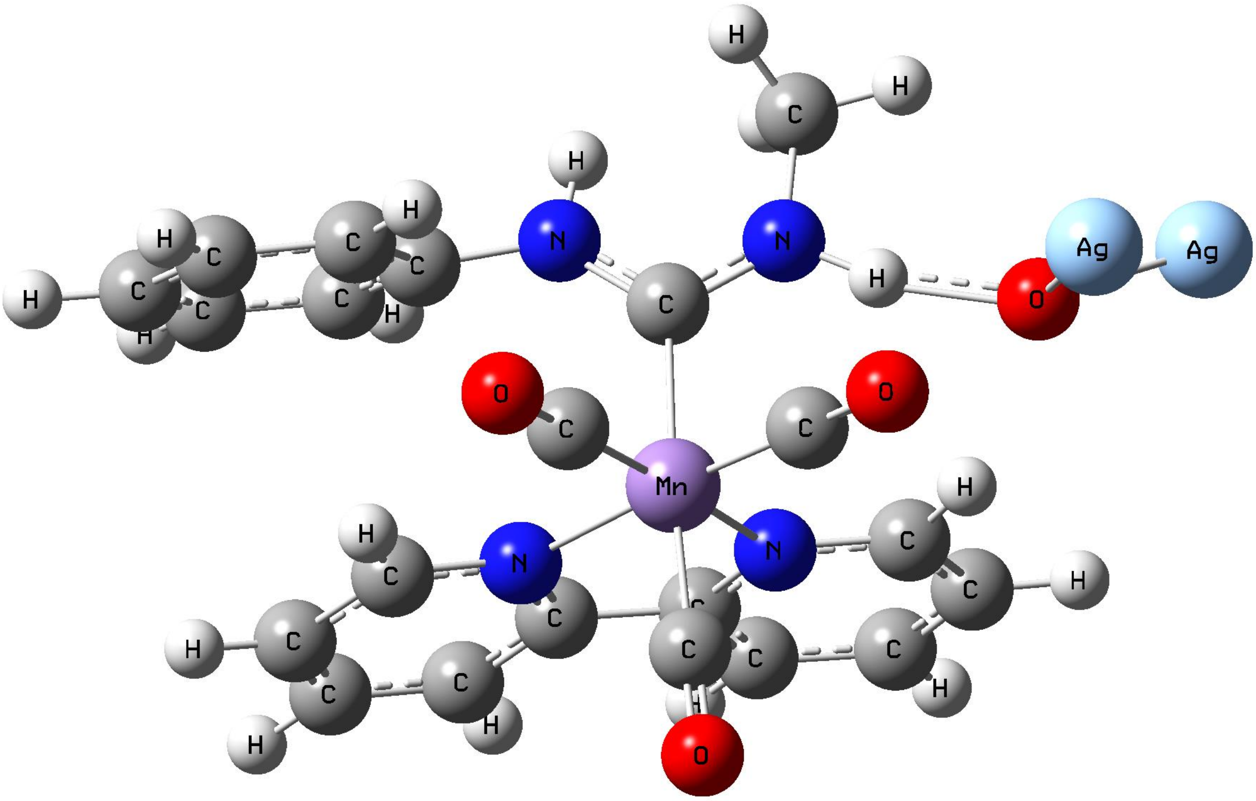

(d) Intermediate **I12**.

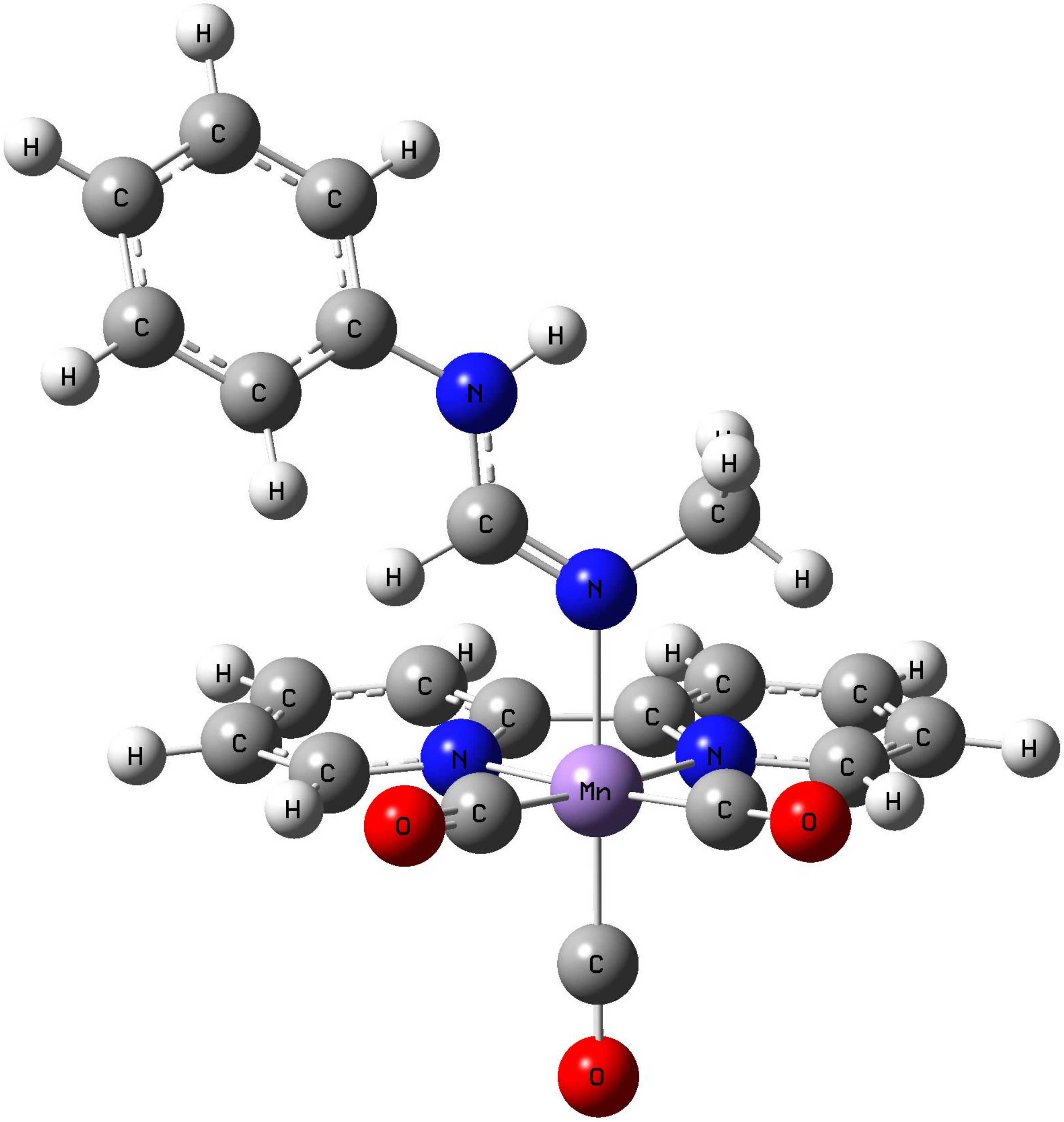

(e) Product **3**.
